# Extending colonic mucosal microbiome analysis—assessment of colonic lavage as a proxy for endoscopic colonic biopsies

**DOI:** 10.1186/s40168-016-0207-9

**Published:** 2016-11-25

**Authors:** Euan Watt, Matthew R. Gemmell, Susan Berry, Mark Glaire, Freda Farquharson, Petra Louis, Graeme I. Murray, Emad El-Omar, Georgina L. Hold

**Affiliations:** 1 Gastrointestinal Research Group, School of Medicine, Medical Sciences and Nutrition, University of Aberdeen, Aberdeen, AB25 2ZD UK; 2 Centre for Genome Enabled Biology and Medicine, School of Medicine, Medical Sciences and Nutrition, University of Aberdeen, Aberdeen, AB25 2ZD UK; 3 Rowett Institute of Nutrition and Health, School of Medicine, Medical Sciences and Nutrition, University of Aberdeen, Aberdeen, AB25 2ZD UK; 4 Department of Pathology, School of Medicine, Medical Sciences and Nutrition, University of Aberdeen, Aberdeen, AB25 2ZD UK; 5 St George and Sutherland Clinical School, University of New South Wales, St George Hospital, Short Street, Kogarah, Sydney, NSW 2217 Australia

**Keywords:** Colonic lavage, Colonic biopsies, Microbiome analysis, Next-generation sequencing

## Abstract

**Background:**

Sequencing-based analysis has become a well-established approach to deciphering the composition of the gut microbiota. However, due to the complexity of accessing sufficient material from colonoscopic biopsy samples, most studies have focused on faecal microbiota analysis, even though it is recognised that differences exist between the microbial composition of colonic biopsies and faecal samples. We determined the suitability of colonic lavage samples to see if it had comparable microbial diversity composition to colonic biopsies as they are without the limitations associated with sample size. We collected paired colonic biopsies and lavage samples from subjects who were attending for colorectal cancer screening colonoscopy.

**Results:**

Next-generation sequencing and qPCR validation were performed with multiple bioinformatics analyses to determine the composition and predict function of the microbiota. Colonic lavage samples contained significantly higher numbers of operational taxonomic units (OTUs) compared to corresponding biopsy samples, however, diversity and evenness between lavage and biopsy samples were similar. The differences seen were driven by the presence of 12 OTUs which were in higher relative abundance in biopsies and were either not present or in low relative abundance in lavage samples, whilst a further 3 OTUs were present in higher amounts in the lavage samples compared to biopsy samples. However, predicted functional community profiling based on 16S ribosomal ribonucleic acid (rRNA) data indicated minimal differences between sample types.

**Conclusions:**

We propose that colonic lavage samples provide a relatively accurate representation of biopsy microbiota composition and should be considered where biopsy size is an issue.

**Electronic supplementary material:**

The online version of this article (doi:10.1186/s40168-016-0207-9) contains supplementary material, which is available to authorized users.

## Background

The importance of the gut microbiome in human health and disease is unequivocal. Humans have evolved over millennia to develop this complex ecosystem of microorganisms, which provides critical health benefits including regulation of the immune system, metabolic processes and homeostatic control [1–3]. Disruption of this stable microbial balance has been associated with a wide range of disease states including inflammatory bowel disease [4–6] and colorectal cancer [7–9] as well as a number of extra-intestinal conditions including obesity [10–12], diabetes [13, 14], liver disease [15] and autoimmune conditions [16, 17].

Despite major advances in microbiological research over the past two decades, there are still fundamental imperfections with sampling techniques that must be addressed and refined [18–20]. Although the majority of studies have assessed gut microbial diversity through analysis of faecal samples, their ability to reflect microbial diversity at the mucosal surface is inadequate [21–23]. Mucosal biopsies display greater microbial diversity than faecal samples [24] as well as taxonomic and phylogenetic differences [25] and contrasting dominant bacterial populations [22]. Colonic mucosal biopsies have always been regarded as the sampling *gold standard,* but there is a limit to how this approach could cope with the competing demands for different starting material (DNA, RNA and proteins) required for modern multi-omic technologies.

Given the limitations of available biopsy material and the known differences between faecal and biopsy microbial communities, we set out to investigate whether colonic lavage was an alternative approach that could provide a comparable assessment of microbial diversity without the limitations associated with biopsy collection. We collected paired sigmoid colonic mucosal biopsies and lavage samples from patients attending for colonoscopy and performed a comprehensive assessment of microbial diversity using 16S ribosomal ribonucleic acid (rRNA) gene sequencing. Our data indicate that colonic lavage samples yield significantly higher amounts of DNA whilst also containing higher microbial counts compared to corresponding biopsy samples although diversity and evenness are similar. Differences between sample types were driven by a limited number of OTUs, which were generally over-represented in biopsies and absent or in lower relative abundance in colonic lavage samples. This difference in diversity corresponded with limited differences in the predicted functional profile highlighting that colonic lavage could be considered for colonic microbial diversity studies where multiple omic analyses are required as colonic lavage samples are not as limited as mucosal biopsies.

## Results

Paired samples (biopsy (Bx) and colonic lavage (CL)) were collected from 23 participants attending for colorectal cancer screening colonoscopy, but with no evidence of pathology seen during colonoscopy examination and subsequent histological analysis which was performed on all participants. Subjects were attending for colonoscopy due to a positive faecal occult blood test but were otherwise asymptomatic and had not taken antibiotics in 6 months prior to colonoscopy. Average age of the subjects was 59.9 years (range 50–75 years), and there was an even split of males and females (Table 1). All participants were from the North East of Scotland and were Caucasian. No information was recorded relating to pro- or prebiotic usage. All participants underwent a similar bowel cleansing procedure. Bowel preparation was good in all subjects in the study. DNA yields were significantly higher from colonic lavage samples compared to that of biopsies, with median DNA yields of 48.5 μg (interquartile range [IQR] 34.5–66.8) for colonic lavage samples and 1.95 μg ([IQR] 1.53–4.3) for biopsies (*p* < 0.0001; Additional file 1: Fig. S1).

**Table 1:** Study cohort information

Cohort metadata	
Number of patients (*N*)	23
Gender (% M:F)	48:52
Average age, in years, at procedure ± SD (range)	59.9 ± 7.9 (50, 75)
Antibiotic use within the previous 6 months	0
Sample location (%)	
Sigmoid colon	100

A total of 3,196,431 16S rRNA sequence reads was obtained following quality filtering, equating to 69487 ± 4907 (mean ± SEM) reads per sample. After the removal of chimeras (12.37%) and non-bacterial sequences (12.38%), the number of mapped sequence reads, per sample, ranged from 11879 to 94103 (mean ± SEM of 60886 ± 4034) (Additional file 2: Table S1). Following the removal of rare OTUs, defined as OTUs with 2 or less sequences across all the samples, which reduced the initial 131669 OTUs down to 17524, rarefaction analysis demonstrated sufficient sequencing depth (Additional file 3: Fig. S2). After removal of rare OTUs, a total of 2,672,954 sequences, equating to 58107 ± 3831 (mean ± SEM) reads per sample remained. A total of 127803 rare sequences was removed, equating to 2778 ± 217 (mean ± SEM) reads per sample.

### Alpha diversity metrics

**Fig. 1:**
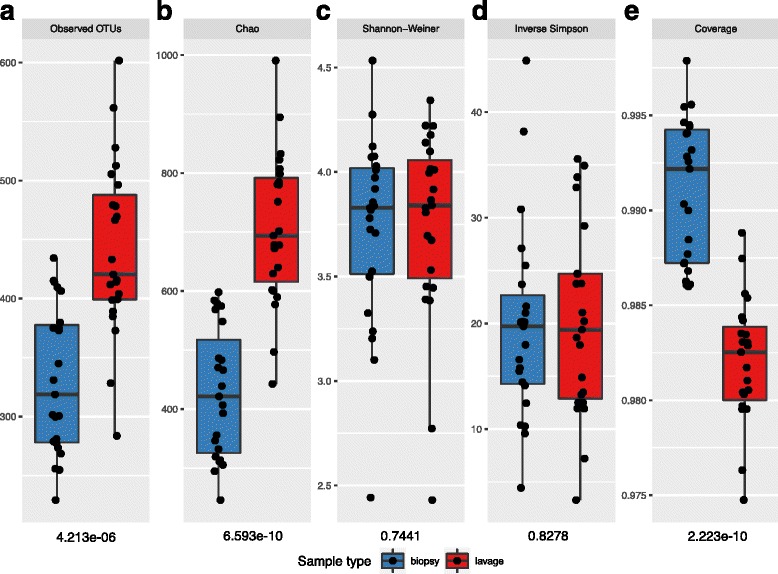
Species diversity comparison between colonic biopsy and lavage sample. The extent of microbiota structural and composition diversities were measured using (**a**) Observed OTUs and (**b**) Chao (species richness), (**c**) Shannon-Weiner diversity index, (**d**) inverse Simpson’s evenness index and (**e**) Good’s coverage (species richness). Alpha diversity scores calculated by subsampling samples to 11000 reads with 1000 iterations. Each point represents the diversity score for a patient sample. *Error bars* represent SEM. Between-group variations were measured using Mann-Whitney *U* test. *P* values of Mann-Whitney *U* test for each alpha diversity measure are below the relevant facet

The effect of sample type (colonic lavage vs biopsy) on alpha diversity was assessed based on OTU richness (measured based on the absolute number of taxa), diversity and evenness (Fig. 1). OTU richness differed significantly between biopsy and lavage sample sets with increased richness present in the lavage samples depicted through Observed OTUs, Chao and Good’s coverage analysis (*p* < 0.0001, all analyses, Mann-Whitney *U* test; Fig. 1a, b and e). However, even with differing numbers of OTUs, the diversity and evenness between colonic lavage and biopsy samples were similar, which was demonstrated by Shannon and inverse Simpson’s scores (Fig. 1c, d).

### Relative abundance analysis

**Fig. 2:**
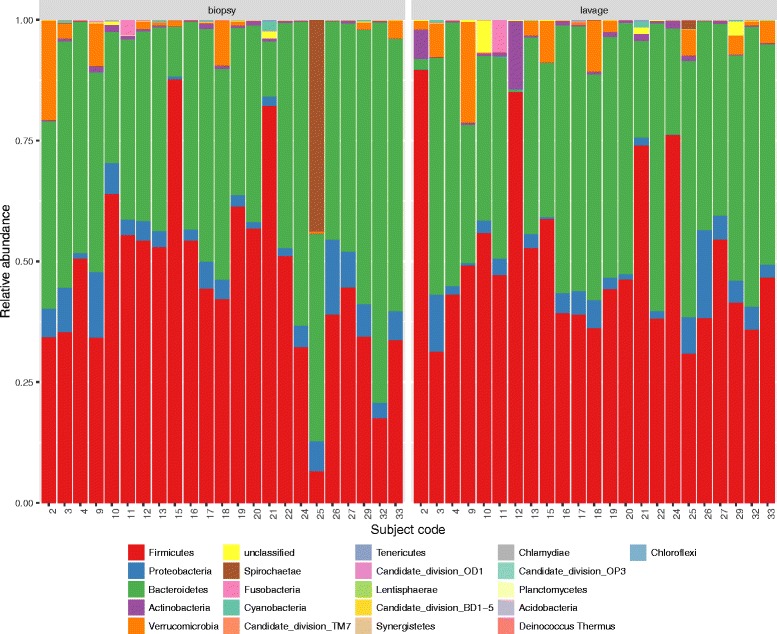
Relative abundance at phylum level for colonic biopsy and lavage samples

**Fig. 3:**
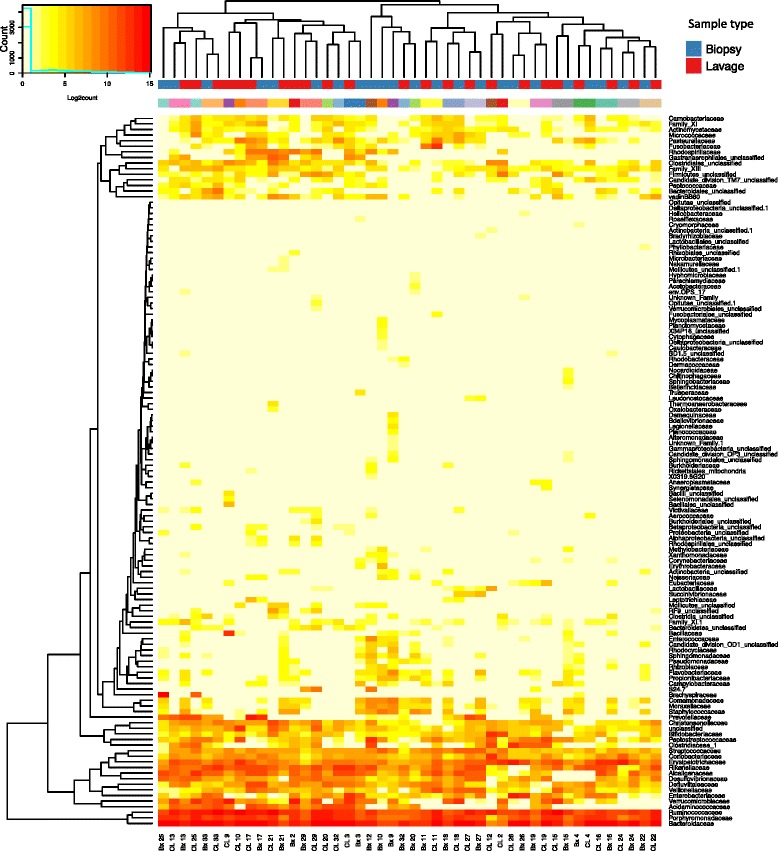
The distribution of bacteria in colonic biopsy and lavage samples at family level. Heat maps show Log2count of sequences within each classification level. Two sets of colours on the column caps: *red*/*blue* depicts sample type (*red* = lavage (CL), *blue* = biopsy (Bx)). *Multicolour panel* reflects individual subjects

The dominant phyla across all samples were *Firmicutes* (median relative abundance, 45.46%; IQR 36.67%, 55.20%), *Bacteroidetes* (43.43%; IQR 37.57%, 50.71%), *Proteobacteria* (3.45%; IQR 1.65%, 5.92%) and *Actinobacteria* (0.47%; IQR 0.24%, 0.79%; Fig. 2). The relative abundance of these four phyla did not differ significantly based on sample type (*p* > 0.05, Wilcoxon rank-sum test; Table 2). qPCR analysis of samples was performed to validate the sequencing approach. Correlation between Illumina sequence data and qPCR data generated R^2^ values of 0.86, 0.88, 0.81 and 0.95 for *Bacteroidaceae, Lachnispiraceae, Ruminococcaceae* and *Enterobacteriaceae,* respectively (Additional file 4: Fig. S3 and Additional file 5). Biopsy and lavage samples did not form discrete clusters by family. Paired subject samples (Bx and CL) of 16 subjects formed distinct clusters at the family level (Fig. 3). Four of the 16 paired subject samples clusters were highly supported by the data with approximately unbiased (AU) *p* values > 95 (Additional file 6: Fig. S4a). The AU *p* values of the other paired subject samples clusters ranged from 58 to 94 with only one below 71.

**Table 2:** Mean difference in the relative abundance of the phyla *Firmicutes*, *Bacteroidetes*, *Proteobacteria* and *Actinobacteria. P* value based on Wilcoxon rank-sum test

Phylum	Mean relative abundance ± SEM		Difference in mean relative abundance ± SEM	
	Biopsy	Lavage		*p* value
*Firmicutes*	46.51 ± 3.81	50.19 ± 3.45	3.68 ± 0.36	0.65
*Bacteroidetes*	43.18 ± 3.04	40.43 ± 3.43	2.75 ± 0.39	1.00
*Proteobacteria*	5.06 ± 0.78	3.83 ± 0.87	1.23 ± 0.09	0.11
*Actinobacteria*	0.52 ± 0.08	1.43 ± 0.63	0.91 ± 0.55	0.23

### Microbiota ordination and taxon distribution

**Fig. 4:**
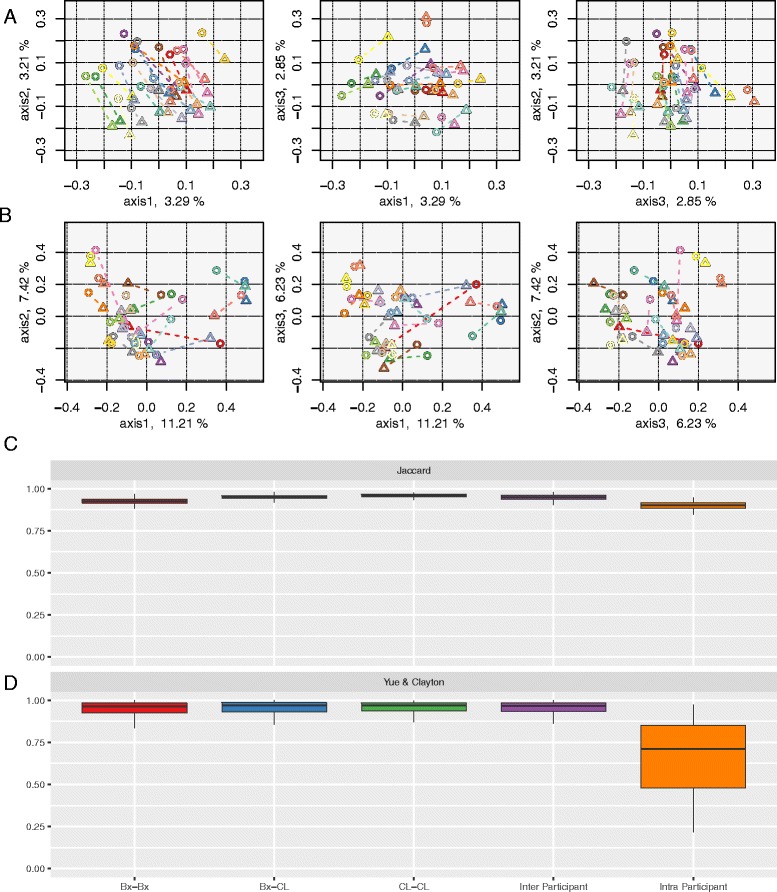
Beta diversity comparisons. Clustering of samples according to sample type (colonic biopsy and lavage) by PCoA, based on (**a**) Jaccard and (**b**) Yue & Clayton similarity distances, and box plots of paired samples’ similarity distances, based on (**c**) Jaccard and (**d**) Yue & Clayton. Within the PCoA plots, loadings of the three axes sum up to 9.35 and 24.86%. Colonic biopsy and lavage samples from the same individual are connected together. Each subject is depicted in a unique colour, and colonic biopsy points are depicted as *triangles* whilst lavage samples are *circles*. Within the *box plots*, *Bx-Bx* refers to all pairs of biopsy samples, *Bx-CL* refers to all biopsy and lavage sample pairs, *CL-CL* refers to all pairs of lavage samples, *inter-participant* refers to all pairs of samples between participants, and *intra-participant* refers to all pairs of samples within participants

Principal coordinate analysis (PCoA) of Jaccard and Yue & Clayton distance measures revealed that samples did not cluster strongly by subject. Some paired subject samples (Bx and CL) did cluster together with regards to OTU richness and OTU community structure (Fig. 4a, b). However, the distances between biopsy and lavage samples were still quite large especially for the Jaccard distances (Fig. 4a). The PCoA plot for Jaccard distances does however demonstrate that the relative direction between the lavage and biopsy sample from the same subject was similar. This may indicate that specific OTUs are shared by the biopsy samples that are not present in lavage samples and vice versa. The picture was less clear with the PCoA plot for Yue & Clayton with no strong clustering by individuals or sample type (Fig. 4b). There appears to be no pattern of shift from the colonic lavage and biopsy samples from the same individual. Consistent with these findings, an AMOVA (analysis of molecular variance) test on the Jaccard dissimilarity matrices indicated that sample type contributed significantly to the differences in microbial composition of the samples (AMOVA *p* = 0.001).

It was found that the Jaccard and Yue & Clayton paired distances of biopsy and lavage samples within participants (e.g. Bx1-CL1, Bx2-CL2, Bx3-CL3) were significantly smaller than all the paired distances of all the samples between participants (e.g. Bx1-Bx2, Bx1-CL2, Bx1-Bx3, Bx1-CL3), the paired distances between all the biopsy samples (e.g. Bx1-Bx2, Bx1-Bx3, Bx1-Bx4), the paired distances between all the biopsy and all the lavage samples (e.g. Bx1-CL1, Bx1-CL2, Bx1-CL3) and the paired distances between all the lavage samples (e.g. CL1-CL2, CL1-CL3, CL1-CL4) (Fig. 4c, d, *p* value < 0.05 for *t* test of unequal variance). The Jaccard paired distances of biopsy and lavage samples within participants were relatively high and similar to the other comparisons (Fig. 4c). The Yue & Clayton paired distances of biopsy and lavage samples within participants were very varied with relatively low values for the 25th and 75th percentiles, ~0.5 and ~0.8 (Fig. 4d). This shows that some of the Bx and CL paired samples of certain participants had more similar communities than within other participants. Overall, these results indicate that intra-participant (within) OTU richness similarities are not much greater compared to that of intra-participant (between) samples. However, community structure similarities are much higher for the inter-participant samples than that of intra-participant samples.

### Correlation

**Fig. 5:**
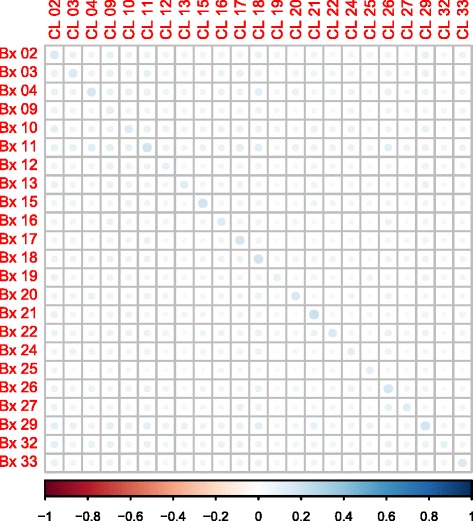
Spearman correlation plot of biopsy against lavage samples using OTU counts. Spearman correlations with non-significant *p* values (>0.05) were excluded

Spearman correlation was carried out to compare the different sample types. This was performed on sample OTU sets after removal of rare OTUs (defined as OTUs with two or less sequences across all the samples) (Fig. 5). Overall, most of the biopsy samples’ highest positive correlation was with its reciprocal lavage sample and vice versa. However, these correlations were small and there were significant, but small, correlations found between the majority of CL and Bx samples.

### Group significance test

**Fig. 6:**
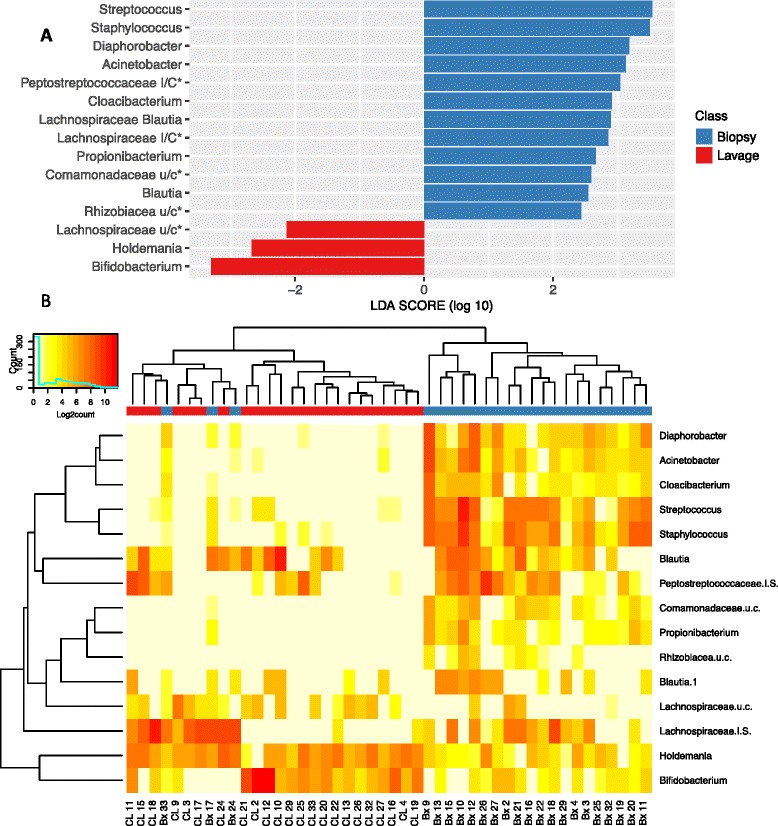
Differentially abundant OTUs between biopsy and lavage samples by LefSe. **a** LEfSe LDA scores. **b** Heat map of Log2count of OTUs. *Colours* on the column caps depict sample type (*red* = lavage (CL), *blue* = biopsy (Bx)). OTUs were labelled by their genera

Linear discriminant analysis effect size (LEfSe) analysis was carried out to determine which OTUs were driving the differences between sample types. These analyses showed that 12 OTUs were present within the biopsy samples that are either not present or in much lower amounts in the lavage samples (Fig. 6a; Additional file 7: Table S2). A further 3 OTUs were present in higher amounts in the lavage samples compared to biopsy samples. Collectively, these 15 OTUs constituted 1.36% of the sequences derived (Table 3). A number of these OTUs represent bacterial species that have been identified as contaminating sequences in previous PCR-based sequencing studies [26]. *Streptococcus, Propionibacterium, Acinetobacter* and *Comamonas* were all reported to be potentially contaminants with *Streptococcus* and *Propionibacterium* being common human skin-associated organisms and were in higher relative abundance in biopsy samples compared to that of lavage samples. Hierarchical clustering based on these 15 OTUs demonstrated robust separation by individual subjects in 20/23 cases (87%; Fig. 6b). Many of the clusters were highly supported by the data with 14 of the CL samples and 9 of the Bx samples within highly supported clusters (AU *p* value > 95) that separate the majority of Bx and CL samples and all other AU *p* values ranging from 68 to 95 (Additional file 6: Fig. S4b).

**Fig. 7:**
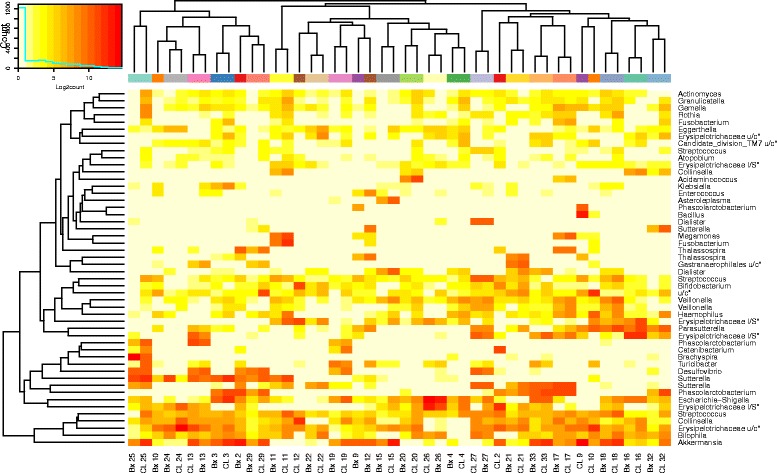
Heat map of Log2count of top 50 OTUs found to not be differentially abundant between biopsy and lavage samples by LEfSe. *Colours* on the column caps reflect individual subjects

Producing a heat map of the top 50 OTUs (which comprised 14.65% of total reads) and excluded OTUs found to be differentially abundant by sample type, showed strong separate clusters formed for most subjects (Table 4; Additional file 8: Table S3). The only subjects whose paired samples did not form separate clusters were subjects 2, 9, 10 and 12 (Fig. 7). Of the 19 paired subject samples that formed distinct clusters by hierarchical clustering, 17 clusters were highly supported (AU *p* value > 95) (Additional file 6: Fig. S4c).

**Table 3:** Information regarding the 15 OTUs detected as biomarkers by LEfSe. Table shows the average amount of reads, average relative abundance and percentage of sequences compared to the total amount of sequences

	Count average	SEM	Average relative abundance	SEM	% of total reads	SEM
All	790.76	121.89	0.0223	0.0057	1.3609	0.0046
Biopsy	803.04	158.75	0.0345	0.0107	0.6910	0.0059
Lavage	778.48	188.59	0.0101	0.0025	0.6699	0.0071

### Contaminant genera assessment

Laboratory contaminants have been shown to affect the results of 16S rRNA samples with low biomass [26]. Genera that were determined to be possible laboratory contaminants were searched for in our dataset. Thirty-three of these genera were detected in the biopsy samples whilst 11 were discovered in the lavage samples with an overlap of 10 genera. These genera were found in higher relative abundances within the biopsy samples compared to the lavage samples (Additional file 9: Fig. S5). A low percentage of the total QC sequences matched these genera with the biopsy and lavage sequences matching these making up 0.35 and 0.59% of the total QC reads.

### Assessment of biopsy and colonic lavage microbial community function

In order to understand the functional differences between communities within biopsies and colonic lavage samples and their relation to community composition, we used phylogenetic investigation of communities by reconstruction of unobserved states (PICRUSt) [27] to infer community metabolic potential (Additional file 5), then used LEfSe [28] to identify functions that differed significantly between sample types.

**Fig. 8:**
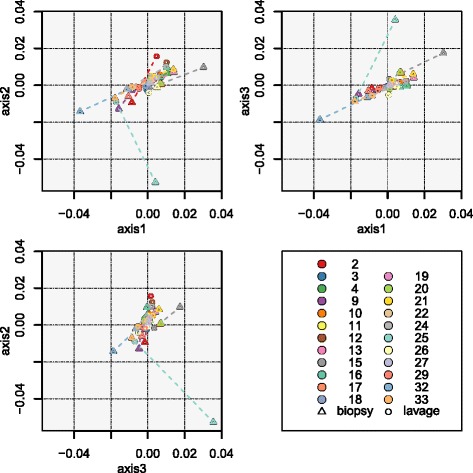
Clustering of samples’ PICRUSt predicted KEGG pathways according to sample type (colonic biopsy and lavage) by NMDS, based on Yue & Clayton similarity distance. The R-squared configuration of the three axes was equal to 0.94413 with a lowest stress of 0.188611. Colonic biopsy and lavage samples from the same individual are connected together. Each subject is depicted in a unique colour. Colonic biopsy points are depicted as *triangles*, lavage samples are *circles*

Very limited functional differences based on kyoto encyclopedia of genes and genomes (KEGG) pathways were seen between biopsy and colonic lavage samples (Additional file 10: Fig. S6a). The three KEGG pathways, pyruvate metabolism, translation proteins and valine, leucine and isoleucine biosynthesis, were found to be in higher relative abundance in colonic lavage samples (Additional file 10: Fig. S6c). One KEGG pathway, the two-component regulatory system, was found to be in higher relative abundance in biopsy samples. The two-component system was shown to have a larger effect size with which it differentiated between the two sample types, compared to the other differential KEGG pathways, with a larger log10 LDA score. Alpha and beta diversity analysis for the predicted KEGG pathways, produced by PICRUSt, were carried out. For alpha diversity the main difference was the higher amount of observed pathways and predicted pathways but difference by coverage was minimal (Additional file 10: Fig. S6b). For beta diversity there was limited evidence of difference between the paired colonic lavage and biopsy samples. Axes one and two did not show a pattern of shift between the biopsy and lavage paired samples, however, axis three showed some differences but with very small axes lengths indicating the differences observed were small (Fig. 8).

## Discussion

Understanding the role of the gut microbiota in disease development is a constantly moving field and as next-generation sequencing approaches become more available we embrace the potential of leveraging multiple omics datasets from a single sample type. The limitations of available biopsy sample size are becoming apparent [29]. Currently, there are no effective experimental approaches that allow retention of pristinely representative DNA, RNA, proteins and other metabolic fractions from limited biopsy samples, in sufficient yield, meaning that scientific aspirations are hampered by clinical sample availability, which is a role reversal from a decade ago when technological capabilities were the bottleneck.

This study is one of the first successful attempts to seek a suitable alternative sample type, namely, colonic lavage, as a reproducible replacement to colonic mucosal biopsies for the purposes of studying the gut microbiome. We comprehensively examined microbial composition and predicted function of patient matched colonic biopsies and lavage samples within 23 individuals. All samples were collected from subjects who had undergone bowel preparation, which may alter microbial alpha and beta diversity [30–32]. However, we reason that all patients were subjected to the same bowel cleansing regime therefore were subjected to the same introduced bias. Patients received bowel preparation the day before the procedure in the form of Picolax. This laxative has two ingredients: sodium picosulfate and magnesium citrate. The former is a stimulant laxative and the latter is an osmotic laxative. The preparation comes in powder form which is reconstituted in 150 ml of cold water before ingestion, and the usual dose is two sachets taken 6 h apart the day prior to the colonoscopy. The patients are encouraged to drink as much clear fluids as possible on the day prior to the procedure to flush the bowel out. Patients were not asked to collect this material as it is impractical, unpleasant and logistically challenging, although this has been done in previous studies, e.g. Jalanka et al. [33], using a different laxative (Moviprep). As the colon is a large and capacious tube, it can never be completely evacuated and inevitably some liquid remains in the bowel. The remaining liquid is therefore bathing the colonic mucosa and tends to shift naturally around the bowel with the aid of peristalsis and the constant changes in the movement and posture of the subject (e.g. supine vs recumbent or semi-recumbent positions). This *lavage* fluid is often encountered at the time of colonoscopy and is very easy to aspirate through a suction channel in the colonoscope and directly into a collecting tube. Both biopsies and colonic lavage samples were collected simultaneously, lavage followed by biopsy, therefore observed relationships are representative of accurate comparisons between sample types and most likely reflect the scope of the majority of such samples available for collection for research studies.

Previous studies which looked at the effect of bowel cleansing treatment have not demonstrated consistent findings. Jalanka et al. [33] demonstrated a decrease in the abundance of the members of Clostridium cluster IV and increased the members of Clostridium cluster XIVa and Proteobacteria. Other studies identifying changes caused by bowel cleansing include Harrell et al. [30], O’Brien et al. [32], Mai et al. [34], Drago et al. [35] and Jain et al. [36], whilst other studies have failed to demonstrate changes [37–39]. The findings of the current study are most relevant to the type of bowel preparation that we used, i.e. Picolax and it is unknown whether other types of bowel preparation would produce different results. It is likely that specific species/taxa will be affected by the bowel prep itself. These questions should be answered in a subsequent study designed to compare the effects of different types of bowel preparation on the gut microbiota. In the current study, our primary focus was on using colonic lavage as a proxy for endoscopic colonic biopsies and as such we sought to reduce potential variation by sticking with one type of bowel preparation.

Knowing that bowel prep may affect microbial diversity and introduce metabolic changes in the microbial community, consideration should be given to assessing the situation in unprepped colonic samples in future studies. The most complete study would need to sample faeces and the unprepped colon prior to bowel prep, followed by additional sampling during prepped colonoscopy and additional faecal sampling post procedure. This approach would provide unequivocal comparison of the effectiveness of the various sample types available. However, logistically such a study will be extremely challenging to conduct. A recent study looked at microbial changes caused by bowel prep in both healthy subjects and patients with IBD. Biopsy and faecal samples were collected pre and post bowel prep but longitudinal faecal follow up was not undertaken. The study demonstrated that bowel prep affected the composition and diversity of the microbiota in the short term differently between sample types with differences also noted between controls and IBD patients. Biopsies from healthy subjects showed greater changes due to bowel prep compared to IBD patients [40].

This study revealed surprising overlap between community composition and function in paired biopsy and colonic lavage samples. This is in contrast to previous studies that have assessed the suitability of faecal samples as an alternative to biopsies for defining microbial diversity at the mucosal surface and have demonstrated significant differences [21–25, 41, 42]. The fact that PICRUSt analysis did not demonstrate differences between (a) biopsy and colonic lavage samples or (b) between patients, potentially highlights the limitation of predicting metabolic functions rather than utilising the more costing metagenomic sequencing option. This lack of difference could be a limitation of PICRUSt due to information only being available on a relatively limited number of species or that sample types did have similar metabolic functions. Unfortunately, until metagenomics become more affordable for such studies, inferred metabolic function will remain a favourable analysis strategy.

One of the main issues to comparing faecal and biopsy microbial diversity is determining when to collect the faecal sample in relation to the colonoscopy sampling to ensure the most appropriate comparisons are made. The benefit of our study was that we were able to sample biopsy and colonic lavage simultaneously thus ensuring an effective representation of community structure devoid of sampling time bias.

The results presented here highlight the emerging concept of the importance of managing potential contaminating sequences—especially in low biomass samples, which are introduced through the analysis pipeline. Within our study, colonic mucosal biopsies were low biomass samples and therefore more likely to be influenced by contaminating bacteria from laboratory reagents [26]. In attempt to control this, we processed and sequenced negative controls as well as PCR processing samples in a random order to ensure avoiding creating false patterns. Biopsies and colonic lavage samples were also extracted using the same DNA extraction kit batches to reduce potential contamination. On this basis, although there is the possibility that these 14 differentially expressed OTUs could be experimentally introduced contaminants, consideration should also be given to biological plausibility of some sequences [43]. The various *Firmicutes* species that are routinely detected in gut microbiota studies including *Blautia* and *Lachnospraceae* sequences potentially demonstrate species that have a higher propensity for attachment to the colonic mucosa and therefore a biologically plausible justification is available. Other less common species including *Comomonadaceae, Staphylococcaceae* and *Propionibacteriaceae* are more challenging to explain and have actually been highlighted to be associated with Qiagen DNA extraction kits [26]. Thirty-four out of the 277 genera in our analysed dataset were classified as possible *contaminant genera*, consisting of 0.94% of the total analysed sequences. This small amount indicates that contamination is not a significant issue within biopsy or lavage samples. However, higher presence of these potential contaminants was detected within biopsy samples, this indicates lavage samples are less affected by contamination, most likely due to the larger biomass available.

## Conclusions

Although colonic biopsies will always give the best representation of the bacterial communities that reside and interact at the gut mucosa, the amount of microbial DNA that can be harnessed is limited and potentially insufficient for certain study types including metagenomic studies or multiple analysis modalities [29]. Our data demonstrates that at least for studies assessing the gut microbiome in prepped patient samples, colonic lavage provides a comparable sample type in terms of gut microbiota community structure but also yields significantly higher amounts of material for multiple analyses. It is appreciated that faecal sample collection still offers the benefit of participants not needing to have a colonoscopy to provide samples and therefore attracting large sample size is not an issue. However, there remains the issue of ensuring faecal sample collection approaches effectively retain microbial community structure through collection and processing which is always challenging in patient-led sample collection. Colonic lavage offers the benefit of sample size that faecal samples provide but from a controllable collection protocol.

## Methods

### Subject recruitment

Study participants were recruited from subjects who had presented for screening colonoscopy as part of the national colorectal cancer screening but were subsequently confirmed to have no macroscopic or microscopic colonic pathology after endoscopic and histological examination. An approach with study information was made by post in advance of admission. Patients were excluded if they received systemic antibiotics 3 months prior to their colonoscopy. All patients received bowel preparation prior to colonoscopy (Picolax) following a standardised protocol given to all colorectal cancer screening participants. Bowel preparation was good in all subjects in the study.

### Sample collection

Patients received bowel preparation the day before the procedure in the form of Picolax (Ferring Pharmaceuticals). This laxative has two active ingredients: sodium picosulfate and magnesium citrate. The former is a stimulant laxative and the latter is an osmotic laxative. The preparation comes in powder form which is reconstituted in 150 ml of cold water before ingestion, and the usual dose is two sachets taken 6 hours apart the day prior to the colonoscopy. The patients are encouraged to drink as much clear fluids as possible on the day prior to the procedure to flush the bowel out. Colonic lavage samples (5 ml per patient, from the sigmoid colon and without contamination by solid faeces) were aspirated from the bowel at the time of colonoscopy using a suction trap, transferred to a separate tube and immediately snap-frozen in liquid nitrogen before being transferred to −80 °C freezer. Biopsies were collected from the sigmoid colon during colonoscopy using standard endoscopic forceps (Boston Scientific Nanterre Cedex, France). Pinch biopsies were either fixed for histological assessment or placed directly into a 1.5-ml Eppendorf tube and snap-frozen in liquid nitrogen and transferred to a −80 °C freezer until further analysis.

### DNA extraction

Genomic DNA was extracted from the colonic mucosal biopsies, two per patient, and colonic lavage samples using the commercially available QIAamp DNA Mini Kit (Qiagen, Crawley, UK) using minor modifications of the manufacturer’s instructions, as previously described which included the addition of 10 μl of Proteinase K during the initial lysis period of 18 hours to ensure complete lysis of the biopsy material prior to DNA extraction [44].

### PCR amplification and sequencing

The V1-2 region of the 16S rRNA gene was amplified using 27 F and 338R primers. The primers were designed with the Illumina adapter overhang already included. Amplification was performed using the Q5 polymerase kit following the manufacturer’s instructions (New England Bio, Ipswich, MA, USA). Post-amplification, samples were purified using AMPure XP (Beckman Coulter, Brea, California, USA) according to manufacturer protocol. The samples were then indexed using the Nextera XT Index Kit V2 (Illumina, San Diego, CA, USA) and KAPA HiFi Hotstart ReadyMix (Kapa Biosystems, Cape Town, South Africa) with a short cycle PCR step followed by a clean-up with AMPure XP. The libraries were quantified using Quant-iT™ dsDNA Assay Kit HS (Thermo Fisher Scientific, Waltham, MA, USA) and analysed on a FLUOstar Omega plate reader (BMG LABTECH, Ortenberg, Germany). The library size was determined using the Agilent 2200 TapeStation (Agilent Technologies, Santa Clara, CA, USA). The libraries were pooled at equimolar concentrations in preparation for sequencing.

Sequencing was performed using an Illumina MiSeq sequencer (Illumina, San Diego, CA, USA) using Illumina V3 chemistry and paired-end 2 × 300 base pair reads. All sequencing was performed in a single MiSeq run. Initial sequence data processing was performed by the Illumina MiSeq Reporter to de-multiplex samples and remove adapter and primer sequences sequence data was exported in the FASTQ format. Sequencing was performed within the Centre for Genome Enabled Biology and Medicine, University of Aberdeen.

### Bioinformatics analysis

The 16S rRNA gene sequence data was further processed using mothur [45] following the MiSeq SOP, which was first accessed in February 2015 [46]. A full record of sequence QC and analysis code carried out with mothur is contained within Additional file 11. The total number of raw paired read sets was 5,396,032 with a mean number of sets of paired reads per sample of 117305.04. After quality control, the samples had a minimum of 11879 reads and mean of 60886, and a mean sequence length of 451 bases with a 2.5 and 95.5 percentile lengths of 440 and 465 bases, respectively.

Alignment and classification were done against the SILVA v119 reference set [47]. Diversity was assessed using the inverse Simpson index [48]. Alpha and beta diversity metrics, Observed OTUs, Chao, Shannon, inverse Simpson, Coverage and Jaccard and Yue & Clayton were calculated using the average score of 1000 random subsamples of the OTUs of each bacterial community to 11000 sequences per sample. These calculations (apart from Chao and Shannon) are the calculations used in the OTU-based analysis section of the Mothur MiSeq SOP [45]. Community structures were compared using PCoA plots generated using the Jaccard and Yue & Clayton distance metrics. PCoA plots were compared with AMOVA within mothur. LefSe was carried on an OTU table subsampled to 11000 sequences per community within mothur [28]. Subsequent statistical analysis was done in R 3.2.2 [49].

**Table 4:** Information regarding the top 50 OTUs not detected as biomarkers by LEfSe. Table shows the average amount of reads, average relative abundance and sequences compared to the total amount of sequences

	Count average	SEM	Average relative abundance	SEM	% of total reads	SEM
All	58108	5002	0.1558	0.0343	14.6615	0.0510
Biopsy	37359	4173	0.1631	0.0506	4.6222	0.0527
Lavage	78856	1875	0.1485	0.0161	10.0393	0.0471

Prior to PICRUSt analysis, mothur-produced OTUs were classified with GreenGenes (August 2013 release of gg_13_8_99) [50]. Metagenome functional content was predicted using PICRUSt [27]. The predicted KEGG orthologs were collapsed into KEGG pathways. Alpha and Beta diversity metrics, Observed KEGGs, Chao, Shannon, inverse Simpson, Coverage and Jaccard and Yue & Clayton were calculated using the average score of 1000 random subsamples of the OTUs of each bacterial community to 500000 KEGG orthologs per sample. LefSe was carried out on the KEGG pathway table normalised to a sum of 1 million orthologs per sample.

Figures were created using R and various R packages. Additional file 12 contains a full record of the figure production carried out in R, this record was created by the R package knitr [51]. Rarefaction plot, alpha diversity plots, taxa pie charts, taxa bar charts and LDA scores figures were created using the ggplot, geom_boxplot, geom_bar and other various functions from the ggplot2 package [52]. Heat maps were constructed using the heatmap.2 function from the gplots package [53]. Correlation plots were produced using the corrplot function from the corrplot package [54]. Colour palettes from the R package RColorBrewer were used within plots [55]. Assessment of hierarchical clustering was carried out using the pvclust and pvrect functions from the pyclust package [56]. Hierarchical clustering in the heat maps and hierarchical assessment were carried out using Euclidean distances with complete hierarchical clustering.

## Additional files

Additional file 1: Figure S1. Comparison of DNA yields between sample types. DNA yields between colonic lavage (CL) and biopsy (Bx) samples. (PDF 44 kb)
Additional file 2: Table S1. Sequencing depth of colonic lavage and biopsy samples from study cohort. (DOC 76 kb)
Additional file 3: Figure S2. Rarefaction curve of all 46 samples following the removal of rare OTUs, defined as OTUs with 2 or less sequences across all the samples, which reduced the initial 131669 OTUs down to 17524) demonstrating sufficient sequencing depth. (PDF 28 kb)
Additional file 4: Figure S3. Correlation between pyrosequencing and qPCR. (PDF 80 kb)
Additional file 5: Real-time PCR. (DOCX 16 kb)
Additional file 6: Figure S4. Assessment of the uncertainty in the hierarchical cluster analysis when clustering. (**A**) Log2 count of sequences within families. (**B**) Differentially abundant OTUs between biopsy and lavage samples by LefSe and (**C**) the top 50 OTUs found to not be differentially abundant between biopsy and lavage samples by LEfSe. The *red* values are the approximately unbiased (AU) *p* values, the *green* values are the bootstrap possibility (BP) values and the *grey* values are the node numbers. Clusters encased by *red squares* indicate clusters with AU *p* values > 95. One thousand bootstraps were utilised. (PDF 80 kb)
Additional file 7: Table S2. LEfSe results for OTUs with an LDA greater than two (effect size) and a significant *p* value (>0.05). Two classes were compared, biopsy samples and colonic lavage samples from the study cohort. Class refers to the sample type with the highest OTU abundance as detected by LEfSe. The LogMax mean is the log of the highest class average. (DOC 48 kb)
Additional file 8: Table S3. Top 50 OTUs found to be similarly abundant between biopsy and colonic lavage samples. (DOC 64 kb)
Additional file 9: Figure S5. Average relative abundance of genera within biopsy and lavage samples matching contaminant genera from Salter et al. 2014 [26]. (PDF 8 kb)
Additional file 10: Figure S6. Predicted KEGG pathway comparison between colonic biopsy and lavage sample. The extent of microbiota predicted functional differences between sample types were measured using (**A**) KEGG pathway abundance and (**B**) Alpha diversity scores. Alpha diversity scores calculated by subsampling samples to 500000 KEGG orthologs with 1000 iterations. Each point represents the diversity score for a patient sample. *Error bars* represent SEM. Between-group variations were measured using Mann-Whitney *U* test. *P* values of Mann-Whitney *U* test for each alpha diversity measure are below the relevant facet, (**C**) LEfSe LDA scores. (PDF 288 kb)
Additional file 11: Full account of QC and analysis carried out with mothur. (TXT 24 kb)
Additional file 12: Full account of figure production by R. (PDF 3.32 mb)
