# Supplementary figures and images for: Extending colonic mucosal microbiome analysis—assessment of colonic lavage as a proxy for endoscopic colonic biopsies
Source: Microbiome. 2016 Nov 25;4:61. doi: 10.1186/s40168-016-0207-9 (PMC5123352; doi:10.1186/s40168-016-0207-9)

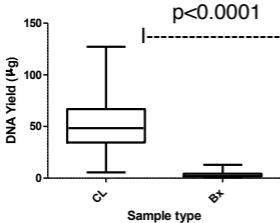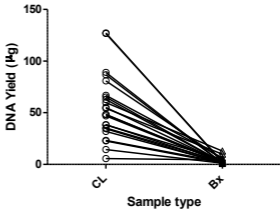

Supplement: Additional file 1: Figure S1. — Comparison of DNA yields between sample types. DNA yields between colonic lavage (CL) and biopsy (Bx) samples. (PDF 44 kb) [file 40168_2016_207_MOESM1_ESM.pdf]

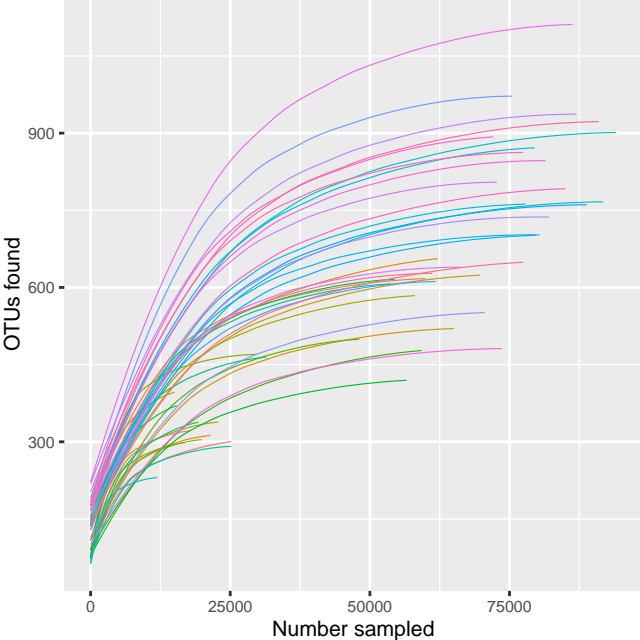

Supplement: Additional file 3: Figure S2. — Rarefaction curve of all 46 samples following the removal of rare OTUs, defined as OTUs with 2 or less sequences across all the samples, which reduced the initial 131669 OTUs down to 17524) demonstrating sufficient sequencing depth. (PDF 28 kb) [file 40168_2016_207_MOESM3_ESM.pdf]

***Bacteroidaceae***  $R^2 = 0.86$

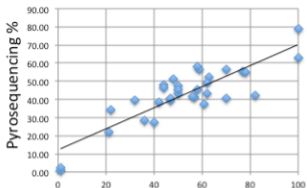

***Lachnospiraceae***  $R^2 = 0.88$

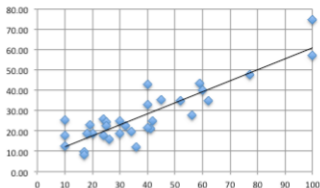

***Ruminococcaceae***  $R^2 = 0.81$

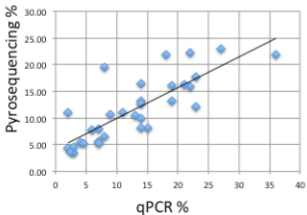

***Enterobacteriaceae***  $R^2 = 0.95$

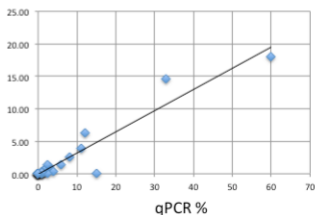

Supplement: Additional file 4: Figure S3. — Correlation between pyrosequencing and qPCR. (PDF 80 kb) [file 40168_2016_207_MOESM4_ESM.pdf]

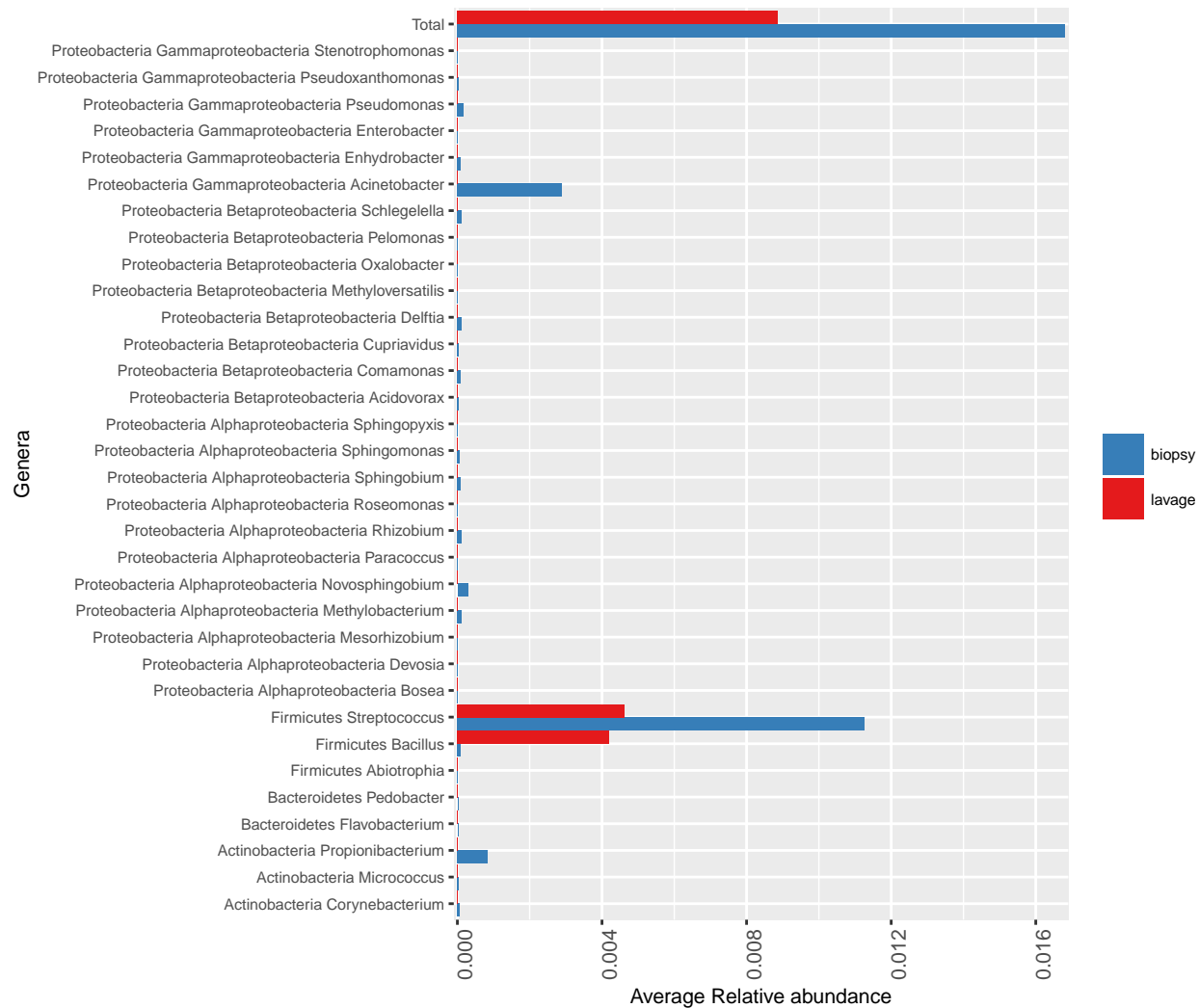

Supplement: Additional file 9: Figure S5. — Average relative abundance of genera within biopsy and lavage samples matching contaminant genera from Salter et al. 2014 [26]. (PDF 8 kb) [file 40168_2016_207_MOESM9_ESM.pdf]
