# Supplementary material for: Extending colonic mucosal microbiome analysis—assessment of colonic lavage as a proxy for endoscopic colonic biopsies
Source: Microbiome. 2016 Nov 25;4:61. doi: 10.1186/s40168-016-0207-9 (PMC5123352; doi:10.1186/s40168-016-0207-9)
Supplement: Additional file 2: Table S1. — Sequencing depth of colonic lavage and biopsy samples from study cohort. (DOC 76 kb) [file 40168_2016_207_MOESM2_ESM.doc]

**Table S1: Sequencing depth of colonic lavage and biopsy samples from study cohort.**

| **Subject code** | **Sample type** | **Paired-raw reads** | **QC contigs pre chimera and non bacterial removal** | **QC Contigs pre rare removal, post chimera removal and non bacterial removal** | **Contigs post rare removal** |
| --- | --- | --- | --- | --- | --- |
| 2 | Biopsy | 125845 | 65359 | 60295 | 58035 |
|  | Lavage | 119198 | 79992 | 72918 | 70558 |
| 3 | Biopsy | 82996 | 34057 | 32818 | 31503 |
|  | Lavage | 143138 | 101700 | 88972 | 85004 |
| 4 | Biopsy | 78435 | 26560 | 26112 | 25146 |
|  | Lavage | 116583 | 82871 | 64728 | 61206 |
| 9 | Biopsy | 74851 | 13259 | 12326 | 11879 |
|  | Lavage | 132918 | 91703 | 80405 | 77459 |
| 10 | Biopsy | 62229 | 19718 | 18373 | 17703 |
|  | Lavage | 160100 | 114841 | 98714 | 94103 |
| 11 | Biopsy | 88604 | 30809 | 26314 | 25202 |
|  | Lavage | 167705 | 124429 | 96041 | 91763 |
| 12 | Biopsy | 127321 | 64957 | 62088 | 59942 |
|  | Lavage | 129602 | 90537 | 81119 | 77817 |
| 13 | Biopsy | 121464 | 68380 | 64647 | 62157 |
|  | Lavage | 134995 | 98321 | 84355 | 79512 |
| 15 | Biopsy | 72771 | 22482 | 22184 | 21407 |
|  | Lavage | 134006 | 95084 | 84117 | 80346 |
| 16 | Biopsy | 141092 | 72037 | 67125 | 65040 |
|  | Lavage | 130150 | 94901 | 83449 | 79634 |
| 17 | Biopsy | 127637 | 80706 | 72821 | 69780 |
|  | Lavage | 150781 | 108105 | 93267 | 88811 |
| 18 | Biopsy | 76280 | 24889 | 23813 | 22911 |
|  | Lavage | 108381 | 72934 | 64878 | 61798 |
| 19 | Biopsy | 74150 | 17508 | 15880 | 15028 |
|  | Lavage | 135875 | 98337 | 80241 | 75533 |
| 20 | Biopsy | 71225 | 21449 | 20840 | 19899 |
|  | Lavage | 138127 | 99066 | 86177 | 82122 |
| 21 | Biopsy | 67693 | 31495 | 31072 | 29811 |
|  | Lavage | 144478 | 102956 | 91592 | 87000 |
| 22 | Biopsy | 76871 | 17948 | 17699 | 16924 |
|  | Lavage | 127855 | 94118 | 77258 | 72739 |
| 24 | Biopsy | 99180 | 59220 | 50381 | 48109 |
|  | Lavage | 123616 | 80019 | 69980 | 66393 |
| 25 | Biopsy | 117606 | 65578 | 61156 | 59260 |
|  | Lavage | 153563 | 110382 | 91516 | 86265 |
| 26 | Biopsy | 144690 | 62992 | 58539 | 56564 |
|  | Lavage | 123911 | 86526 | 76219 | 73647 |
| 27 | Biopsy | 131330 | 64169 | 57151 | 54438 |
|  | Lavage | 147104 | 103500 | 86366 | 81442 |
| 29 | Biopsy | 69051 | 17102 | 16256 | 15403 |
|  | Lavage | 124283 | 89178 | 76226 | 72087 |
| 32 | Biopsy | 94501 | 21394 | 20342 | 19302 |
|  | Lavage | 137775 | 99824 | 81924 | 77436 |
| 33 | Biopsy | 129914 | 62004 | 56256 | 53825 |
|  | Lavage | 156152 | 113035 | 95807 | 91011 |
