# Supplementary material for: Extending colonic mucosal microbiome analysis—assessment of colonic lavage as a proxy for endoscopic colonic biopsies
Source: Microbiome. 2016 Nov 25;4:61. doi: 10.1186/s40168-016-0207-9 (PMC5123352; doi:10.1186/s40168-016-0207-9)
Supplement: Additional file 5: — Real-time PCR. (DOCX 16 kb) [file 40168_2016_207_MOESM5_ESM.docx]

**Additional file 4**

**Extending colonic mucosal microbiome analysis - Assessment of colonic lavage as a proxy for endoscopic colonic biopsies**

Euan Watt^a,1^, Matthew R. Gemmell ^b,1,^, Susan Berry^a^, Mark Glaire^a^, Petra Louis^c^, Graeme I. Murray^d^, Emad El-Omar^a,2^, Georgina L. Hold^a,3^

**Additional file 4 –** Real-time PCR

Quantitative real-time PCR was performed as described previously [1,2]. Briefly, standard curves consisted of ten-fold dilution series of amplified bacterial 16S rRNA genes from reference strains. Samples were amplified with universal primers against total bacteria and specific primers against *Bacteroidetes*, Firmicutes clostridial cluster IV, Firmicutes clostridial cluster XIVa and *Enterobacteriaceae*. The abundance of 16S rRNA gene copies was determined from standard curves and specific bacterial groups were expressed as a percentage of total bacteria determined by universal primers. Due to the relatively low abundance of bacterial DNA in biopsy samples, 25ng of DNA was used per reaction whilst colonic lavage samples used 5ng of DNA per reaction. Visual inspection of sample amplification curves compared to standard curves indicated that this only lead to minor inhibition for a few of the samples. Furthermore, the same DNA concentration was used for all runs, including universal primer runs which were used to normalise specific bacterial groups against total bacteria, to minimize errors due to any inhibitory substances in the samples. The detection limit was determined with negative controls containing only herring sperm DNA.

**Supplementary References**

1. Ramirez-Farias C*,* et al. Effect of inulin on the human gut microbiota: Stimulation of *Bifidobacterium adolescentis* and *Faecalibacterium prausnitzii.* Br J Nutr. 2009;101:541-50.

2. Hansen R*,* et al. Microbiota of de-novo pediatric IBD: Increased *Faecalibacterium prausnitzii* and reduced bacterial diversity in Crohn's but not in ulcerative colitis*.* Am J Gastroenterol. 2012;107:1913-22.
