# Supplementary material for: Extending colonic mucosal microbiome analysis—assessment of colonic lavage as a proxy for endoscopic colonic biopsies
Source: Microbiome. 2016 Nov 25;4:61. doi: 10.1186/s40168-016-0207-9 (PMC5123352; doi:10.1186/s40168-016-0207-9)
Supplement: Additional file 7: Table S2. — LEfSe results for OTUs with an LDA greater than two (effect size) and a significant p value (>0.05). Two classes were compared, biopsy samples and colonic lavage samples from the study cohort. Class refers to the sample type with the highest OTU abundance as detected by LEfSe. The LogMax mean is the log of the highest class average. (DOC 48 kb) [file 40168_2016_207_MOESM7_ESM.doc]

**Table S2: LEfSe results for OTUs with a LDA greater than 2 (effect size) and a significant p-value (> 0.05).** Two classes were compared, biopsy samples and colonic lavage samples from the study cohort. Class refers to the sample type with the highest OTU abundance as detected by LEfSe. The LogMaxMean is the Log of the highest class average.

| OTU | Tax Classification | LogMaxMean | Class | LDA | pValue |
| --- | --- | --- | --- | --- | --- |
| OTU20 | Bacteria Firmicutes Bacilli Bacillales Staphylococcaceae Staphylococcus | 3.81 | Biopsy | 3.50 | 1.43E-08 |
| OTU21 | Bacteria Actinobacteria Actinobacteria Bifidobacteriales Bifidobacteriaceae Bifidobacterium | 3.65 | Lavage | 3.30 | 0.0207228 |
| OTU22 | Bacteria Firmicutes Erysipelotrichia Erysipelotrichales Erysipelotrichaceae Holdemania | 3.11 | Lavage | 2.68 | 0.0208542 |
| OTU27 | Bacteria Firmicutes Bacilli Lactobacillales Streptococcaceae Streptococcus | 3.85 | Biopsy | 3.54 | 3.10E-06 |
| OTU35 | Bacteria Proteobacteria Betaproteobacteria Burkholderiales Comamonadaceae Diaphorobacter | 3.46 | Biopsy | 3.18 | 7.62E-08 |
| OTU46 | Bacteria Proteobacteria Gammaproteobacteria Pseudomonadales Moraxellaceae Acinetobacter | 3.41 | Biopsy | 3.13 | 2.36E-08 |
| OTU58 | Bacteria Bacteroidetes Flavobacteriia Flavobacteriales Flavobacteriaceae Cloacibacterium | 3.18 | Biopsy | 2.91 | 7.57E-08 |
| OTU66 | Bacteria Actinobacteria Actinobacteria Propionibacteriales Propionibacteriaceae Propionibacterium | 2.88 | Biopsy | 2.66 | 5.35E-06 |
| OTU73 | Bacteria Proteobacteria Betaproteobacteria Burkholderiales Comamonadaceae U/C | 2.76 | Biopsy | 2.60 | 1.40E-05 |
| OTU114 | Bacteria Proteobacteria Alphaproteobacteria Rhizobiales Rhizobiaceae U/C | 2.25 | Biopsy | 2.43 | 8.38E-05 |
| OTU117 | Bacteria Firmicutes Clostridia Clostridiales Lachnospiraceae Blautia | 3.40 | Biopsy | 2.89 | 0.0359655 |
| OTU130 | Bacteria Firmicutes Clostridia Clostridiales Peptostreptococcaceae IS | 3.46 | Biopsy | 3.04 | 0.0165814 |
| OTU150 | Bacteria Firmicutes Clostridia Clostridiales Lachnospiraceae Incertae_Sedis | 3.47 | Biopsy | 2.86 | 0.040143 |
| OTU305 | Bacteria Firmicutes Clostridia Clostridiales Lachnospiraceae Blautia | 2.84 | Biopsy | 2.55 | 0.0262619 |
| OTU307 | Bacteria Firmicutes Clostridia Clostridiales Lachnospiraceae unclassified | 2.40 | Lavage | 2.13 | 0.0164062 |
