# Supplementary material for: Extending colonic mucosal microbiome analysis—assessment of colonic lavage as a proxy for endoscopic colonic biopsies
Source: Microbiome. 2016 Nov 25;4:61. doi: 10.1186/s40168-016-0207-9 (PMC5123352; doi:10.1186/s40168-016-0207-9)
Supplement: Additional file 8: Table S3. — Top 50 OTUs found to be similarly abundant between biopsy and colonic lavage samples. (DOC 64 kb) [file 40168_2016_207_MOESM8_ESM.doc]

**Table S3: Top 50 OTUs found to be similarly abundant between biopsy and colonic lavage samples.**

| OTU | Tax Classification |
| --- | --- |
| Otu000001 | Bacteria Verrucomicrobia Verrucomicrobiae Verrucomicrobiales Verrucomicrobiaceae Akkermansia |
| Otu000002 | Bacteria Proteobacteria Gammaproteobacteria Enterobacteriales Enterobacteriaceae Escherichia-Shigella |
| Otu000003 | Bacteria Firmicutes Erysipelotrichia Erysipelotrichales Erysipelotrichaceae unclassified |
| Otu000004 | Bacteria Proteobacteria Betaproteobacteria Burkholderiales Alcaligenaceae Sutterella |
| Otu000005 | Bacteria Proteobacteria Betaproteobacteria Burkholderiales Alcaligenaceae Sutterella |
| Otu000006 | Bacteria Firmicutes Erysipelotrichia Erysipelotrichales Erysipelotrichaceae Incertae_Sedis |
| Otu000007 | Bacteria Firmicutes Erysipelotrichia Erysipelotrichales Erysipelotrichaceae Incertae_Sedis |
| Otu000008 | Bacteria Firmicutes Negativicutes Selenomonadales Acidaminococcaceae Phascolarctobacterium |
| Otu000009 | Bacteria Firmicutes Bacilli Lactobacillales Streptococcaceae Streptococcus |
| Otu000010 | Bacteria Proteobacteria Deltaproteobacteria Desulfovibrionales Desulfovibrionaceae Desulfovibrio |
| Otu000011 | Bacteria Spirochaetae Spirochaetes Spirochaetales Brachyspiraceae Brachyspira |
| Otu000012 | Bacteria Actinobacteria Coriobacteriia Coriobacteriales Coriobacteriaceae Collinsella |
| Otu000013 | Bacteria Proteobacteria Betaproteobacteria Burkholderiales Alcaligenaceae Parasutterella |
| Otu000014 | Bacteria Proteobacteria Deltaproteobacteria Desulfovibrionales Desulfovibrionaceae Bilophila |
| Otu000015 | Bacteria Firmicutes Erysipelotrichia Erysipelotrichales Erysipelotrichaceae Incertae_Sedis |
| Otu000016 | Bacteria Firmicutes Negativicutes Selenomonadales Veillonellaceae Veillonella |
| Otu000017 | Bacteria Proteobacteria Gammaproteobacteria Pasteurellales Pasteurellaceae Haemophilus |
| Otu000018 | Bacteria Firmicutes Bacilli Bacillales Bacillaceae Bacillus |
| Otu000019 | Bacteria unclassified unclassified unclassified unclassified unclassified |
| Otu000023 | Bacteria Firmicutes Bacilli Lactobacillales Streptococcaceae Streptococcus |
| Otu000024 | Bacteria Firmicutes Negativicutes Selenomonadales Acidaminococcaceae Phascolarctobacterium |
| Otu000025 | Bacteria Actinobacteria Actinobacteria Bifidobacteriales Bifidobacteriaceae Bifidobacterium |
| Otu000026 | Bacteria Actinobacteria Actinobacteria Actinomycetales Actinomycetaceae Actinomyces |
| Otu000028 | Bacteria Firmicutes Erysipelotrichia Erysipelotrichales Erysipelotrichaceae Catenibacterium |
| Otu000029 | Bacteria Fusobacteria Fusobacteriia Fusobacteriales Fusobacteriaceae Fusobacterium |
| Otu000030 | Bacteria Firmicutes Negativicutes Selenomonadales Veillonellaceae Megamonas |
| Otu000031 | Bacteria Firmicutes Erysipelotrichia Erysipelotrichales Erysipelotrichaceae Turicibacter |
| Otu000032 | Bacteria Firmicutes Bacilli Bacillales Family_XI Gemella |
| Otu000033 | Bacteria Firmicutes Negativicutes Selenomonadales Veillonellaceae Dialister |
| Otu000034 | Bacteria Firmicutes Negativicutes Selenomonadales Veillonellaceae Veillonella |
| Otu000036 | Bacteria Proteobacteria Betaproteobacteria Burkholderiales Alcaligenaceae Sutterella |
| Otu000037 | Bacteria Firmicutes Negativicutes Selenomonadales Veillonellaceae Dialister |
| Otu000038 | Bacteria Firmicutes Negativicutes Selenomonadales Acidaminococcaceae Acidaminococcus |
| Otu000039 | Bacteria Proteobacteria Alphaproteobacteria Rhodospirillales Rhodospirillaceae Thalassospira |
| Otu000040 | Bacteria Actinobacteria Actinobacteria Micrococcales Micrococcaceae Rothia |
| Otu000041 | Bacteria Firmicutes Bacilli Lactobacillales Carnobacteriaceae Granulicatella |
| Otu000042 | Bacteria Actinobacteria Coriobacteriia Coriobacteriales Coriobacteriaceae Eggerthella |
| Otu000043 | Bacteria Firmicutes Erysipelotrichia Erysipelotrichales Erysipelotrichaceae unclassified |
| Otu000044 | Bacteria Cyanobacteria Melainabacteria Gastranaerophilales unclassified unclassified |
| Otu000045 | Bacteria Fusobacteria Fusobacteriia Fusobacteriales Fusobacteriaceae Fusobacterium |
| Otu000047 | Bacteria Firmicutes Erysipelotrichia Erysipelotrichales Erysipelotrichaceae Asteroleplasma |
| Otu000048 | Bacteria Proteobacteria Gammaproteobacteria Enterobacteriales Enterobacteriaceae Klebsiella |
| Otu000049 | Bacteria Candidate_division_TM7 unclassified unclassified unclassified unclassified |
| Otu000050 | Bacteria Actinobacteria Coriobacteriia Coriobacteriales Coriobacteriaceae Collinsella |
| Otu000051 | Bacteria Proteobacteria Alphaproteobacteria Rhodospirillales Rhodospirillaceae Thalassospira |
| Otu000052 | Bacteria Firmicutes Erysipelotrichia Erysipelotrichales Erysipelotrichaceae Incertae_Sedis |
| Otu000053 | Bacteria Actinobacteria Coriobacteriia Coriobacteriales Coriobacteriaceae Atopobium |
| Otu000054 | Bacteria Firmicutes Bacilli Lactobacillales Streptococcaceae Streptococcus |
| Otu000055 | Bacteria Firmicutes Negativicutes Selenomonadales Acidaminococcaceae Phascolarctobacterium |
| Otu000056 | Bacteria Firmicutes Bacilli Lactobacillales Enterococcaceae Enterococcus |
