# Supplementary material for: Extending colonic mucosal microbiome analysis—assessment of colonic lavage as a proxy for endoscopic colonic biopsies
Source: Microbiome. 2016 Nov 25;4:61. doi: 10.1186/s40168-016-0207-9 (PMC5123352; doi:10.1186/s40168-016-0207-9)

Extending colonic mucosal microbiome analysis - Assessment of colonic lavage as a proxy for endoscopic colonic biopsies

Euan Watt, MBChB, BSc(Hons); Matthew R Gemmell, BSc(Hons), MSc; Susan H Berry, BSc; Mark Glaire, MBChB, BSc(Hons); Freda Farquharson, BSc, MSc; Petra Louis, BSc; PhD; Graeme Murray, MBChB, PhD; Emad M El-Omar, MBChB, BSc(Hons), MD; Georgina Louise Hold, PhD

This document contains all figure production carried out in R for the manuscript.

All data to reproduce analysis can be found here: https://github.com/m-gemmell/Watt_etal_16sBxCL (https://github.com/m-gemmell/Watt_etal_16sBxCL)

Figure 1: Species diversity comparison between colonic biopsy and lavage sample

Wilcox test to compare the Alpha diversity values of Biopsy and Lavagae samples

```
data <- read.csv("colonoscopy.makecontigsfile.trim.contigs.good.unique.good.filter
.unique.precluster.pick.pick.an.unique_list.0.03.pick.groups.ave.txt", sep="\t", r
ow.names = 1)
Bx <- data[1:23,]
FA <- data[24:46,]
#Mannwhitney u test
data$type<-c(rep("Bx",23), rep("FA",23))
data$type <- as.factor(data$type)
wilcox.test(sobs~type, data=data)
```

```
##
##  Wilcoxon rank sum test
##
## data:  sobs by type
## W = 68, p-value = 4.213e-06
## alternative hypothesis: true location shift is not equal to 0
```

```
wilcox.test(chao~type, data=data)
```

```
##
##  Wilcoxon rank sum test
##
## data:  chao by type
## W = 20, p-value = 6.593e-10
## alternative hypothesis: true location shift is not equal to 0
```

```
wilcox.test(shannon~type, data=data)
```

```
##
##  Wilcoxon rank sum test
##
## data:  shannon by type
## W = 249, p-value = 0.7441
## alternative hypothesis: true location shift is not equal to 0
```

```
wilcox.test(invsimpson~type, data=data)
```

```
##
##  Wilcoxon rank sum test
##
## data:  invsimpson by type
## W = 254, p-value = 0.8278
## alternative hypothesis: true location shift is not equal to 0
```

```
wilcox.test(coverage~type, data=data)
```

```
##
##  Wilcoxon rank sum test
##
## data:  coverage by type
## W = 513, p-value = 2.223e-10
## alternative hypothesis: true location shift is not equal to 0
```

Box plots for alpha diversity value comparisons

```r
### Load packages or install if not present
if (!require("RColorBrewer")) {
  install.packages("RColorBrewer")
  library(RColorBrewer)}
```

```
## Loading required package: RColorBrewer
```

```r
if (!require("ggplot2")) {install.packages("ggplot2")
  library(ggplot2)}
```

```
## Loading required package: ggplot2
```

```r
if (!require("tidyr")) {install.packages("tidyr")
  library(tidyr)}
```

```
## Loading required package: tidyr
```

```
#Files
alpha_file="colonoscopy.makecontigsfile.trim.contigs.good.unique.good.filter.uniqu
e.precluster.pick.pick.an.unique_list.0.03.pick.groups.ave.txt"
metadata_file="16sBxCL_Metadata.txt"
#Read in files
alpha_data <- read.csv(alpha_file,sep="\t", row.names = 1)
metadata <- read.csv(metadata_file,sep="\t", row.names = 1)
#Order alpha_data and metadata so they are the same order
alpha_data <- alpha_data[order(row.names(alpha_data)),]
metadata <- metadata[order(row.names(metadata)),]
#Check if row names match
stopifnot(identical(row.names(alpha_data), row.names(metadata)))
#Merge data frames
plot_data_metadata <- merge(x=alpha_data, y=metadata, by="row.names")
#Fix row names after merge
row.names(plot_data_metadata) <- plot_data_metadata[,1]
plot_data_metadata <- plot_data_metadata[,-1]
#Remove unwanted columns
plot_data_metadata <- plot_data_metadata[,c(-3,-4,-6,-7,-9,-10)]
#Change name of alpha diversity values
colnames(plot_data_metadata)[1:5] <- c("Observed OTUs", "Chao","Shannon-Weiner","I
nverse Simpson", "Coverage")
#Convert to long list format
alpha_long <- gather(plot_data_metadata, Alpha_diversity_measure, value, 1:5)
#Reorder measures
alpha_long$Alpha_diversity_measure_f <- factor(alpha_long$Alpha_diversity_measure,
levels=c("Observed OTUs", "Chao","Shannon-Weiner","Inverse Simpson", "Coverage"))
#Set colours
fa_b_colour <- brewer.pal(9, "Set1")
#Box Plot for alpha diversity comparing lavage against biopsy
g <- ggplot(alpha_long, aes(x=new.Sample.type, y=value, fill=new.Sample.type)) + g
eom_boxplot(outlier.colour = NA) + theme_set(theme_gray(base_size = 8)) +facet_wra
p(~ Alpha_diversity_measure_f, nrow=1, scales="free") + geom_point(position = posi
tion_jitter(width = 0.2)) + scale_x_discrete(breaks=NULL, name="") + scale_fill_ma
nual(values=c(fa_b_colour[2], fa_b_colour[1]), name="Sample type") + scale_y_conti
nuous(name="") + theme(legend.position="bottom", legend.margin=unit(0,"cm"), plot.
margin =unit(c(0,0,0,0),"mm"))
ggsave("Fig1.pdf", g, units="mm", height=120, width=170, dpi=300)
```

```
## [1] TRUE
```

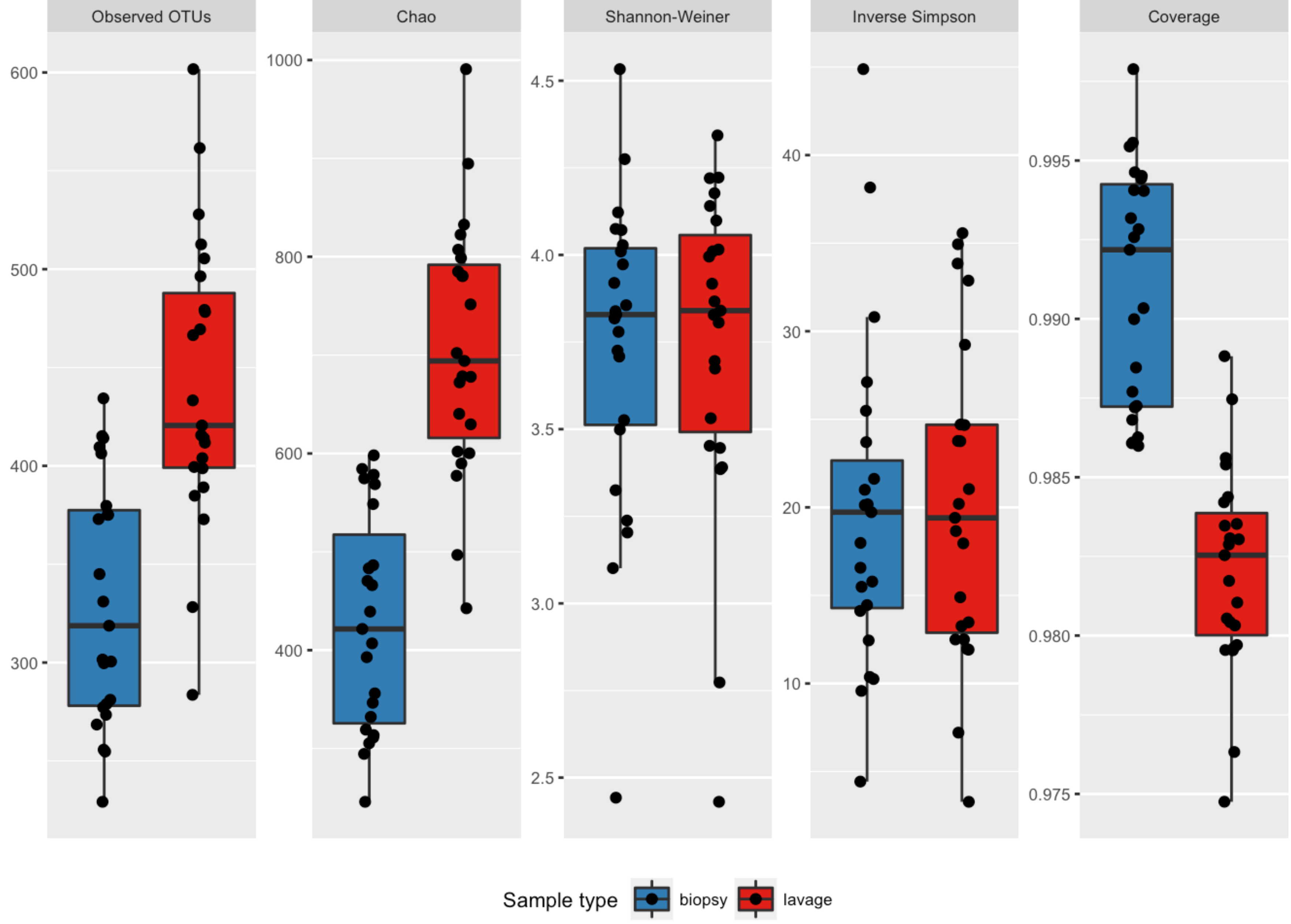

Figure 2: Relative abundance at phylum level for colonic biopsy and lavage samples.

```
### Load packages or install if not present
if (!require("RColorBrewer")) {install.packages("RColorBrewer")
  library(RColorBrewer)}
if (!require("ggplot2")) {install.packages("ggplot2")
  library(ggplot2)}
if (!require("tidyr")) {install.packages("tidyr")
  library(tidyr)}
#Read in files
data <- read.csv("16sBxCL_phylum_phylotype_summary.txt", sep = "\t", row.names = 1
)
metadata <- read.csv("16sBxCL_Metadata.txt", sep = "\t", row.names = 1)
#Order data and metadata so they are the same order
data <- data[order(row.names(data)),]
metadata <- metadata[order(row.names(metadata)),]
#Check if row names match
stopifnot(identical(row.names(metadata), row.names(data)))
#Merge data
plot_data_metadata <- merge(x=data, y=metadata, by="row.names")
row.names(plot_data_metadata) <- plot_data_metadata[,1]
plot_data_metadata <- plot_data_metadata[,-1]
#Change BD1.5 to BD1-5
colnames(plot_data_metadata)[14] <- "Candidate_division_BD1-5"
#Change Deinococcus.Thermus to Deinococcus Thermus
colnames(plot_data_metadata)[20] <- "Deinococcus Thermus"
#Change data to long list format
plot_data_metadata_long <- gather(plot_data_metadata, Phylum, relabund, Firmicutes
:Chloroflexi)
#Colours for phyla
colset <- c(brewer.pal(8, "Set1"), brewer.pal(8, "Set2"), brewer.pal(12, "Set3"),
brewer.pal(9, "Pastel1"))
#Change Participant numbers into strings
plot_data_metadata_long$Individual <- as.character(plot_data_metadata_long$Individ
ual)
#Change order of phylum factor and individual factor
plot_data_metadata_long$Phylum <- factor(plot_data_metadata_long$Phylum,levels = u
nique(plot_data_metadata_long$Phylum))
plot_data_metadata_long$Individual <- factor(plot_data_metadata_long$Individual,le
vels = c(2,3,4,9:13,15:22,24:29,32,33))
#Bar chart
g_bar <- ggplot(plot_data_metadata_long, aes(x = Individual, y=relabund, fill=Phyl
um)) +geom_bar(stat = 'identity', position = 'stack', width=0.9) + facet_wrap( ~ n
ew.Sample.type, nrow=1) + scale_fill_manual(values = colset) + theme_set(theme_gra
y(base_size = 8)) + theme(axis.text.x = element_text(angle = 90, hjust = 1)) + xla
b("Subject code") + ylab("Relative abundance") + scale_y_continuous(expand = c(0,0
)) + geom_text(aes(x=1, y=1.00, label="Stretch it"), vjust=-1) + theme(legend.posi
tion="bottom", legend.key.size = unit(4, "mm"), legend.margin=unit(0,"cm"),plot.ma
rgin =unit(c(0,0,0,0),"mm")) + labs(fill='')
ggsave("Fig2.pdf", g_bar, units="cm", height=14, width=17, dpi=300)
```

```
## [1] TRUE
```

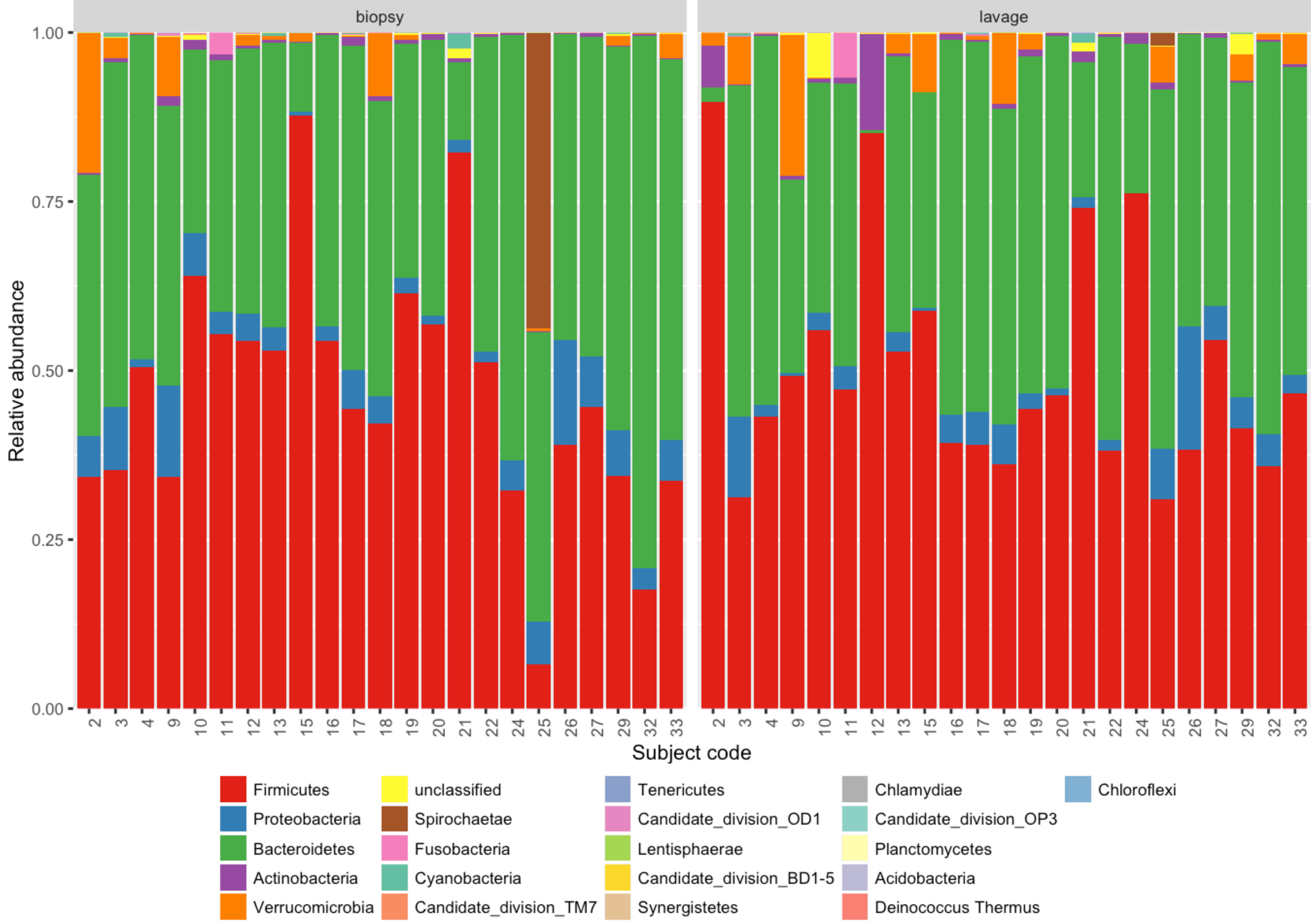

Figure 3: The distribution of bacteria in colonic biopsy and lavage samples at Family Level

2 heatmaps produced to give the colours for sample type and particpant for each column The two heatmaps were combined using powerpoint

```
### Load packages or install if not present
if (!require("arules")) {install.packages("arules")
  library(arules)}
```

```
## Loading required package: arules
```

```
## Loading required package: Matrix
```

```
##
## Attaching package: 'Matrix'
```

```
## The following object is masked from 'package:tidyr':
##
##     expand
```

```
##
## Attaching package: 'arules'
```

```
## The following objects are masked from 'package:base':
##
##     abbreviate, write
```

```
if (!require("ade4")) {install.packages("ade4")
  library(ade4)}
```

```
## Loading required package: ade4
```

```
if (!require("vegan")) {install.packages("vegan")
  library(vegan)}
```

```
## Loading required package: vegan
```

```
## Loading required package: permute
```

```
## Loading required package: lattice
```

```
## This is vegan 2.4-1
```

```
##
## Attaching package: 'vegan'
```

```
## The following object is masked from 'package:ade4':
##
##     cca
```

```
if (!require("gdata")) {install.packages("gdata")
  library(gdata)}
```

```
## Loading required package: gdata
```

```
## gdata: read.xls support for 'XLS' (Excel 97-2004) files ENABLED.
```

```
##
```

```
## gdata: read.xls support for 'XLSX' (Excel 2007+) files ENABLED.
```

```
##
## Attaching package: 'gdata'
```

```
## The following object is masked from 'package:stats':
##
##     nobs
```

```
## The following object is masked from 'package:utils':
##
##     object.size
```

```
## The following object is masked from 'package:base':
##
##     startsWith
```

```
if (!require("gplots")) {install.packages("gplots")
  library(gplots)}
```

```
## Loading required package: gplots
```

```
##
## Attaching package: 'gplots'
```

```
## The following object is masked from 'package:stats':
##
##     lowess
```

```
if (!require("RColorBrewer")) {install.packages("RColorBrewer")
  library(RColorBrewer)}
#Load in data
data <- read.csv("16sBxCL_family_phylotype_log2counts_summary.txt", sep="\t", row.
names=1)
metadata_info <- read.csv("16sBxCL_Metadata.txt", sep="\t", row.names=1)
#Remove unwanted column
heatmap_info <- data[,-1]
sample_info <- metadata_info$Sample.type
#Selecting a colour palette to be used in figure
brewer_colours <- brewer.pal(9, "Set1")
#Order heatmap_info and metadata so they are the same order
heatmap_info <- heatmap_info[order(row.names(heatmap_info)),]
metadata_info <- metadata_info[order(row.names(metadata_info)),]
#Check if row names match
stopifnot(identical(row.names(metadata_info), row.names(heatmap_info)))
#Setting colours to sample_types
sample_type_colours <- gsub("aspirate",brewer_colours[1], sample_info)
sample_type_colours <- gsub("biopsy", brewer_colours[2], sample_type_colours)
#Transposing heatmap_info
heatmap.info.t <- t(heatmap_info)
#producing heatmaps
pdf("Fig3A.pdf", 170/25.4,height=182.4/25.4)
heatmap.2(as.matrix(heatmap.info.t), ColSideColors=sample_type_colours,margins = c
(2,8),key.xlab="Log2count", key.title=NA,trace="none", col=function(x)rev(heat.col
ors(x)), labRow = row.names(heatmap.info.t), cexRow = 0.55, cexCol = 0.55, offsetR
ow=0.0000001, offsetCol = 0.0000001, labCol = metadata_info$new.Sample.ID, key.par
=list(mar=c(1,3,1,1), cex=0.3), lwid=c(1,4), lhei=c(1,9))
dev.off()
```

```
## quartz_off_screen
##                  2
```

```
pdf("Fig3B.pdf",170/25.4, height=182.4/25.4)
heatmap.2(as.matrix(heatmap.info.t), ColSideColors=as.character(metadata_info$Colo
ur), margins = c(2,8), key.xlab="Log2count", key.title=NA, trace="none", col=funct
ion(x)rev(heat.colors(x)), labRow = row.names(heatmap.info.t), cexRow = 0.55, cexC
ol = 0.55, offsetRow=0.0000001, offsetCol = 0.0000001, labCol = metadata_info$new.
Sample.ID, key.par=list(mar=c(1,3,1,1), cex=0.3), lwid=c(1,4), lhei=c(1,9))
dev.off()
```

```
## quartz_off_screen
##                  2
```

```
## [1] TRUE
```

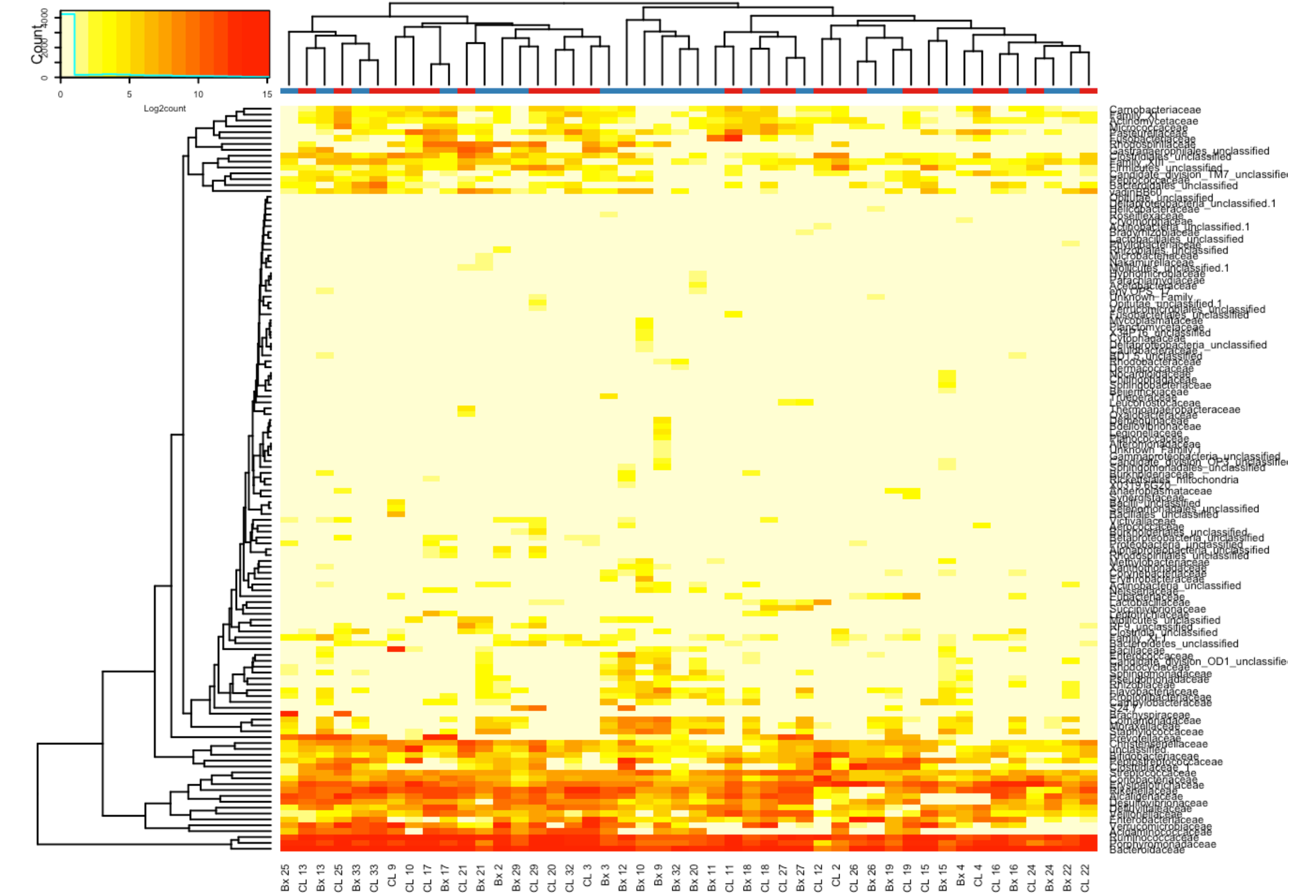
Count
0
2000
4000
0
5
10
15
Log2count
Carnobacteriaceae
Bacteroidaceae
Bx 25
CL 13
Bx 13
CL 25
Bx 33
CL 33
CL 9
CL 10
CL 17
Bx 17
CL 21
Bx 21
Bx 2
Bx 29
CL 29
CL 20
CL 32
CL 3
Bx 3
Bx 12
Bx 10
Bx 9
Bx 32
Bx 20
Bx 11
CL 11
Bx 18
CL 18
CL 27
Bx 27
CL 12
CL 2
CL 26
Bx 26
Bx 19
CL 19
CL 15
Bx 15
Bx 4
CL 4
CL 16
Bx 16
CL 24
Bx 24
Bx 22
CL 22

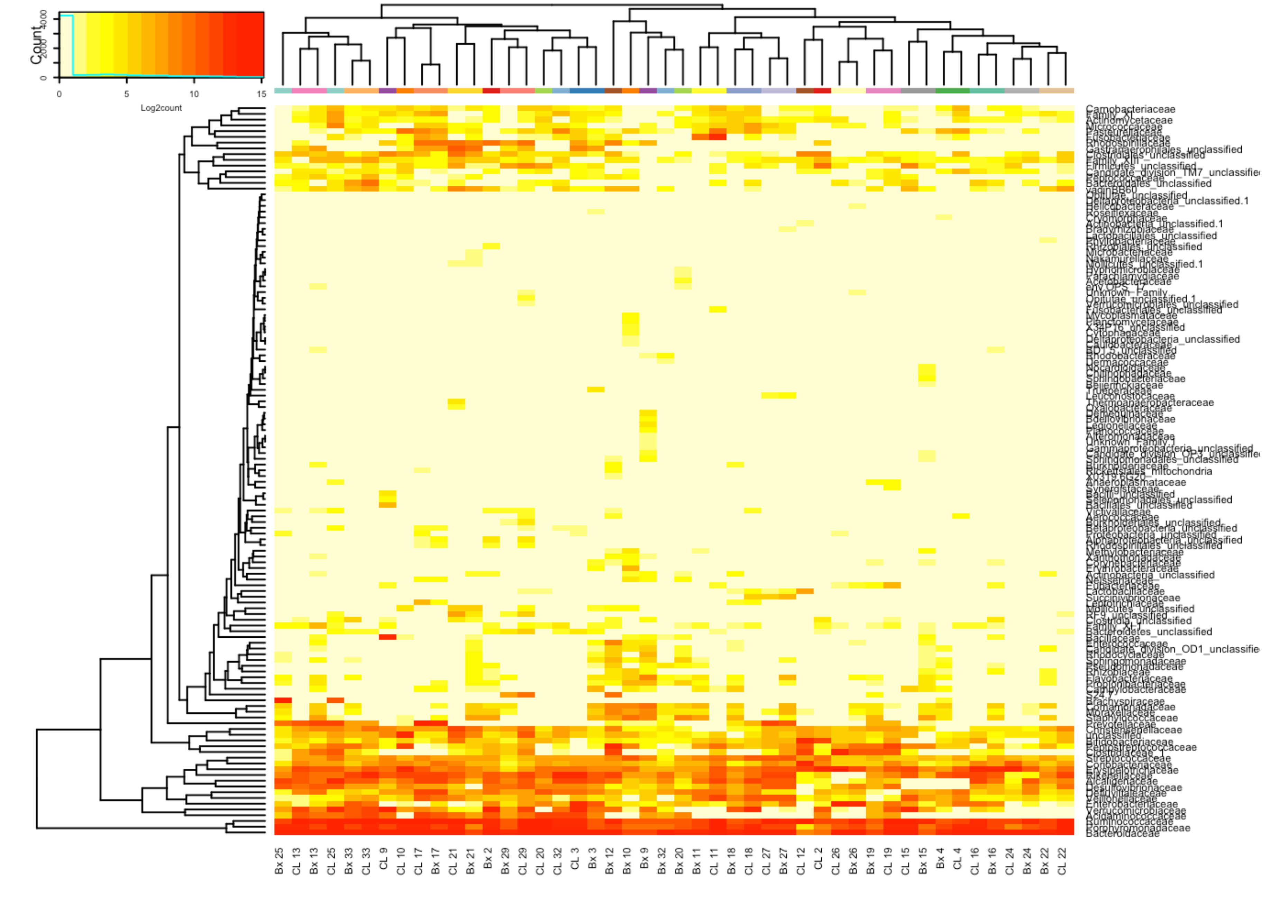
Count
0
2000
4000
0
5
10
15
Log2count
Carnobacteriaceae
Bacteroidaceae
Bx 25
CL 13
Bx 13
CL 25
Bx 33
CL 33
CL 9
CL 10
CL 17
Bx 17
CL 21
Bx 21
Bx 2
Bx 29
CL 29
CL 20
CL 32
CL 3
Bx 3
Bx 12
Bx 10
Bx 9
Bx 32
Bx 20
Bx 11
CL 11
Bx 18
CL 18
CL 27
Bx 27
CL 12
CL 2
CL 26
Bx 26
Bx 19
CL 19
CL 15
Bx 15
Bx 4
CL 4
CL 16
Bx 16
CL 24
Bx 24
Bx 22
CL 22

Figure 4: Beta diveristy measures

A & B) Clustering of samples according to sample type (colonic biopsy and lavage) by PCoA based on (A) Jaccard and (B) Yue & Clayton similarity distance

```
#Function to produce plot and PDF of plot
produce_plot <- function(axes_file, metadata_file, loadings_file, PDF_file, SAMPLE
, PAIRED){
  ### Load package or install if not present
  if (!require("RColorBrewer")) {
    install.packages("RColorBrewer")
    library(RColorBrewer)
  }
#Reads in data
plot_data <- read.csv(axes_file,sep="\t", row.names = 1)
metadata <- read.csv(metadata_file,sep="\t", row.names = 1)
loadings_info <- read.csv(loadings_file,sep="\t", row.names = 1)
#Order plot_data and metadata so they are the same order
plot_data <- plot_data[order(row.names(plot_data)),]
metadata <- metadata[order(row.names(metadata)),]
#Check if row names match and stop if not
stopifnot(identical(row.names(plot_data), row.names(metadata)))
#Merge data frames
plot_data_metadata <- merge(x=plot_data, y=metadata, by="row.names")
#Fix rownames after merge
row.names(plot_data_metadata) <- plot_data_metadata[,1]
plot_data_metadata <- plot_data_metadata[,-1]
#Sets columns to variables
axis1 <- plot_data_metadata[,1]
axis2 <- plot_data_metadata[,2]
axis3 <- plot_data_metadata[,3]
#Set the minimum and maximum x so each plot is the same size as each other for com
parison
maxx <- max(c(max(axis1),max(axis2),max(axis3)))
minx <- min(c(min(axis1),min(axis2),min(axis3)))
#Grouping to colour by
group_samples <- as.factor(plot_data_metadata[,SAMPLE])
#Creates plot points for first and second samples
#This data will be used to connect the points together
first_level <- levels(plot_data_metadata[,PAIRED])[1]
second_level <- levels(plot_data_metadata[,PAIRED])[2]
first_rows <- plot_data_metadata[plot_data_metadata[,PAIRED]==first_level,]
second_rows <- plot_data_metadata[plot_data_metadata[,PAIRED]==second_level,]
#Order data to get colouring correct
first_rows <- first_rows[order(first_rows[,SAMPLE]),]
second_rows <- second_rows[order(second_rows[,SAMPLE]),]
first_group_samples <- as.factor(first_rows[,SAMPLE])
```

```
second_group_samples <- as.factor(second_rows[,SAMPLE])
#Select colour palette for plot
col.brew <- c(brewer.pal(9, "Set1"),brewer.pal(8,"Set2"),brewer.pal(12,"Set3"))
palette(col.brew)
#Assignment of loading values
axis_1_lab <- paste("axis1, ", round(loadings_info[1,1], digits=2), "%")
axis_2_lab <- paste("axis2, ", round(loadings_info[2,1], digits=2), "%")
axis_3_lab <- paste("axis3, ", round(loadings_info[3,1], digits=2), "%")
#Function to produce single 2d plot
plot_production <- function(nx, ny, xlabel, ylabel){
ps <- 1
  #Make an empty plotting area with axis labels
  plot(0,0, xlab = xlabel, ylab = ylabel, xlim=c(minx-0.05, maxx+0.05), ylim=c(min
x-0.05, maxx+0.05), pch=19,col=NA, type='b')
  #Sets colour of background of plot
  rect(par("usr")[1], par("usr")[3], par("usr")[2], par("usr")[4], col = "#f6f6f6"
)
  #Sets gridlines that match the ticks of the axes
  grid (NULL,NULL, lty = 6, col = "black", lwd=0.3)
  #Add black borders to points
  points(first_rows[,nx], first_rows[,ny], type='p', pch=2, col="black", cex=ps+0.
1)
  points(second_rows[,nx], second_rows[,ny], type='p', pch=1, col="black", cex=ps+
0.1)
  points(first_rows[,nx], first_rows[,ny], type='p', pch=2, col="black", cex=ps-0.
1)
  points(second_rows[,nx], second_rows[,ny], type='p', pch=1, col="black", cex=ps-
0.1)
  #This part will plot pair by pair
  #This is so pairs only connect to each other by the dashed lines
  for (i in 1:num_samples){
    n <- i
    points(c(first_rows[n,nx],second_rows[n,nx]), c(first_rows[n,ny],second_rows[n
,ny]), type='b', pch=c(2,1), lwd=ps, col=i, cex=ps, lty=2)
  }
}
#Save plot to pdf file
pdf(PDF_file, width = 170/25.4, height = 50/25.4)
#Creates diagram to put PCoA plots into
par(mfrow=c(1,3), oma = c(0.8,0.8,0,0),
    mgp= c(2,1,0), mar=c(3,3,0.5,0.5), cex=0.7)
num_samples <- nrow(first_rows)
# creating the 3 2d plots
plot_production(1,2,axis_1_lab,axis_2_lab)
plot_production(1,3,axis_1_lab,axis_3_lab)
plot_production(3,2,axis_3_lab,axis_2_lab)
dev.off()
}
#Jaccard PCoA plots
#File names of input data
axes_file_name <- "colonoscopy.makecontigsfile.trim.contigs.good.unique.good.filte
r.unique.precluster.pick.pick.an.unique_list.0.03.pick.jclass.0.03.lt.ave.pcoa.axe
s"
metadata_file_name <- "16sBxCL_Metadata.txt"
```

```
loadings_file_name <- "colonoscopy.makecontigsfile.trim.contigs.good.unique.good.f
ilter.unique.precluster.pick.pick.an.unique_list.0.03.pick.jclass.0.03.lt.ave.pcoa
.loadings"
#Name of PDF output file
pdf_file_name <- "Fig4A.pdf"
#Relevant metadata info
paired <- "Sample.type"
sample <- "Individual"
#Produce plot
produce_plot(axes_file_name, metadata_file_name, loadings_file_name, pdf_file_name
, sample, paired)
```

```
## quartz_off_screen
##                 2
```

```
#yue & Clayton PCoA plots
#File names of input data
axes_file_name <- "colonoscopy.makecontigsfile.trim.contigs.good.unique.good.filte
r.unique.precluster.pick.pick.an.unique_list.0.03.pick.thetayc.0.03.lt.ave.pcoa.ax
es"
metadata_file_name <- "16sBxCL_Metadata.txt"
loadings_file_name <- "colonoscopy.makecontigsfile.trim.contigs.good.unique.good.f
ilter.unique.precluster.pick.pick.an.unique_list.0.03.pick.thetayc.0.03.lt.ave.pco
a.loadings"
#Name of PDF output file
pdf_file_name <- "Fig4B.pdf"
#Relevant metadata info
paired <- "Sample.type"
sample <- "Individual"
#Produce plot
produce_plot(axes_file_name, metadata_file_name, loadings_file_name, pdf_file_name
, sample, paired)
```

```
## quartz_off_screen
##                 2
```

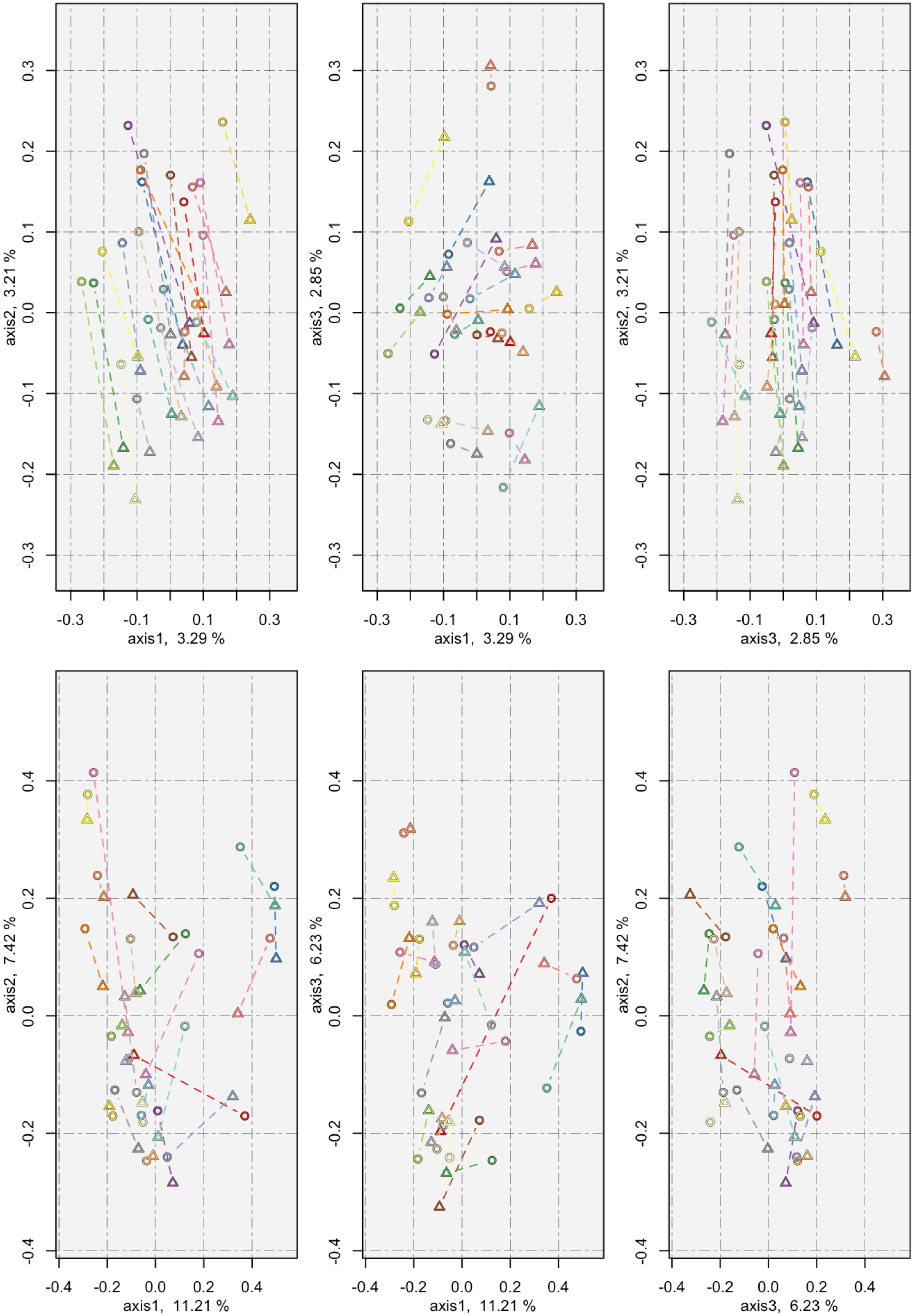

axis1, 3.29 %
axis2, 3.21 %
axis3, 2.85 %
axis1, 3.29 %
axis3, 2.85 %
axis2, 3.21 %
-0.3
-0.1
0.1
0.3
-0.3
-0.2
-0.1
0.0
0.1
0.2
0.3
axis1, 11.21 %
axis2, 7.42 %
axis1, 11.21 %
axis3, 6.23 %
axis3, 6.23 %
axis2, 7.42 %
-0.4
-0.2
0.0
0.2
0.4

C & D) Intra and inter-subject beta diversity related distance measures.

```
### Load the package or install if not present
if (!require("RColorBrewer")) {install.packages("RColorBrewer")
  library(RColorBrewer)}
if (!require("ggplot2")) {install.packages("ggplot2")
  library(ggplot2)}
if (!require("tidyr")) {install.packages("tidyr")
  library(tidyr)}
#Read in Jaccard  & thetayc info
tri_jaccard <- read.csv("16sBxCL_avg_jaccard.dist", sep = "\t")
tri_thetayc <- read.csv("16sBxCL_avg_yue_clayton.dist", sep = "\t")
#Convert to long
tri_jaccard_long <- gather(tri_jaccard, SAMPLE_2, paired_dist, 2:(ncol(tri_jaccard
)))
tri_thetayc_long <- gather(tri_thetayc, SAMPLE_2, paired_dist, 2:(ncol(tri_thetayc
)))
#Change name of column 1
colnames(tri_jaccard_long)[1] <- "SAMPLE_1"
colnames(tri_thetayc_long)[1] <- "SAMPLE_1"
#Remove rows with NA
tri_jaccard_long <- tri_jaccard_long[complete.cases(tri_jaccard_long),]
tri_thetayc_long <- tri_thetayc_long[complete.cases(tri_thetayc_long),]
#Add a column to indicate distance type
tri_jaccard_long$calc <- "Jaccard"
tri_thetayc_long$calc <- "Yue & Clayton"
#Row bind the dataframes to make one with all the info
long_data <- rbind(tri_jaccard_long, tri_thetayc_long)
#Change FA to CL
long_data$SAMPLE_1 <- gsub("FA", "CL", long_data$SAMPLE_1)
long_data$SAMPLE_2 <- gsub("FA", "CL", long_data$SAMPLE_2)
#Make columns with only Participant info
long_data$SAMPLE_1_PARTICIPANT <- gsub("_S.*", "", long_data$SAMPLE_1)
long_data$SAMPLE_1_PARTICIPANT <- gsub("Test[A-z]{2}", "", long_data$SAMPLE_1_PART
ICIPANT)
long_data$SAMPLE_2_PARTICIPANT <- gsub("_S.*", "", long_data$SAMPLE_2)
long_data$SAMPLE_2_PARTICIPANT <- gsub("Test[A-z]{2}", "", long_data$SAMPLE_2_PART
ICIPANT)
#Make columns with only sample type info
long_data$SAMPLE_1_TYPE <- gsub("Test","",long_data$SAMPLE_1)
long_data$SAMPLE_1_TYPE <- gsub("[0-9].*","",long_data$SAMPLE_1_TYPE)
long_data$SAMPLE_2_TYPE <- gsub("Test","",long_data$SAMPLE_2)
long_data$SAMPLE_2_TYPE <- gsub("[0-9].*","",long_data$SAMPLE_2_TYPE)
#Now a column to determine if the paired dist is intra or inter participant
long_data$intra_participant <- long_data$SAMPLE_1_PARTICIPANT == long_data$SAMPLE_
2_PARTICIPANT
#Give an informative name
long_data$intra_participant <- gsub("TRUE", "Intra Participant", long_data$intra_p
articipant)
long_data$intra_participant <- gsub("FALSE", "Inter Participant", long_data$intra_
participant)
```

```
#Column to indicate if it is Bx-Bx, CL-CL or Bx-CL
long_data$SAMPLE_TYPES <- paste0(long_data$SAMPLE_1_TYPE, "-", long_data$SAMPLE_2_
TYPE)
long_data$SAMPLE_TYPES <- gsub("CL-Bx", "Bx-CL", long_data$SAMPLE_TYPES)
#create a long list format with
#Column for paired dist
#Calc
#Descriptiotn including Inter, Intra, BX-BX BX-CL and CL-CL
#First creat a long list for Inter & intra info and rename intra column
Inter_Intra_long <- long_data[,c("paired_dist", "calc", "intra_participant")]
colnames(Inter_Intra_long)[3] <- "description"
#Next the same for the Sample types info
Sample_types_long <- long_data[,c("paired_dist", "calc", "SAMPLE_TYPES")]
colnames(Sample_types_long)[3] <- "description"
#now row bind the two long lists
description_long <- rbind(Inter_Intra_long, Sample_types_long)
#Set colours
col.brew <- c(brewer.pal(9, "Set1"),brewer.pal(8,"Set2"),brewer.pal(12,"Set3"))
#now produce the box plots facetted by calc
g_box <- ggplot(description_long, aes(x=description, y=paired_dist, fill=descripti
on)) +
  #boxplot
  geom_boxplot(outlier.colour = "NA", weight=0.1, size = 0.2) +
  #facte by calc
  facet_wrap(~calc, nrow = 2) +
  labs(x = "", y = "") +
  scale_fill_manual(values=col.brew) +
  #Changes size of text to equivalent of word point size
  theme_grey(base_size = 8) +
  #remove legend
  theme(legend.position="none") +
  #Set so y scale goes from 0 to 1
  scale_y_continuous(limits = c(0, 1))
#This command will save the above g_bar object as a pdf
#Can change the size of the PDF
ggsave("Fig4CD.pdf", g_box, units="mm", height=100, width=170, dpi=300)
```

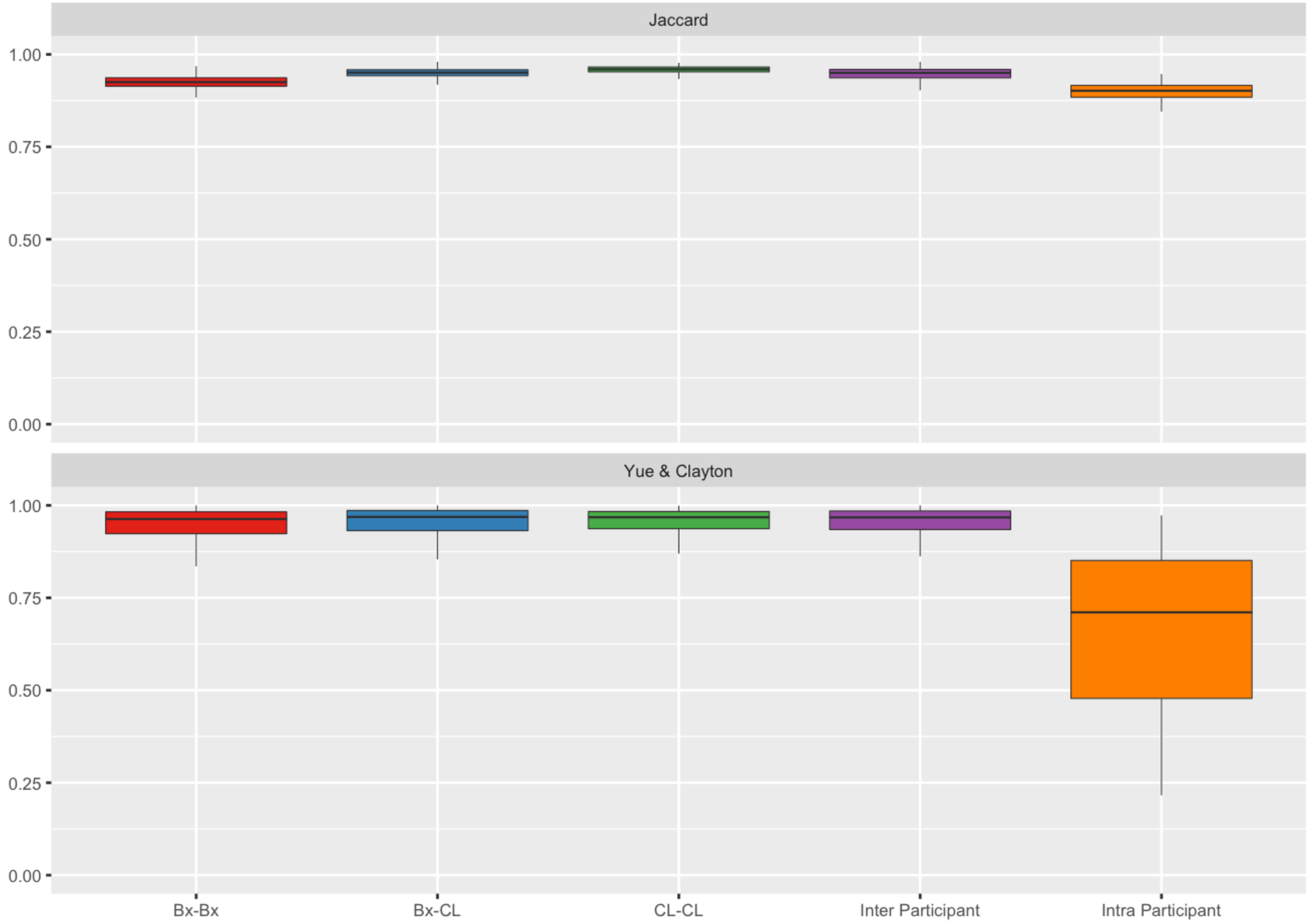

Figure 5: Spearman correlation plot of biopsy against lavage samples using OTU counts

```
### Load packages or install if not present
if (!require("ggplot2")) {install.packages("ggplot2")
  library(ggplot2)}
if (!require("Hmisc")) {install.packages("Hmisc")
  library(Hmisc)}
```

```
## Loading required package: Hmisc
```

```
## Loading required package: survival
```

```
## Loading required package: Formula
```

```
##
## Attaching package: 'Hmisc'
```

```
## The following object is masked from 'package:gdata':
##
##     combine
```

```
## The following objects are masked from 'package:base':
##
##     format.pval, round.POSIXt, trunc.POSIXt, units
```

```
if (!require("corrplot")) {install.packages("corrplot")
  library(corrplot)}
```

```
## Loading required package: corrplot
```

```
if (!require("ggplot2")) {install.packages("ggplot2")
  library(ggplot2)}
#Load in data
otu_info <- read.csv("colonoscopy.makecontigsfile.trim.contigs.good.unique.good.fi
lter.unique.precluster.pick.pick.an.unique_list.0.03.pick.shared", sep="\t", row.n
ames = 2, header=TRUE)
metadata_info <- read.csv("16sBxCL_Metadata.txt", sep="\t", row.names=1)
#remove unwanted columns
otu_info <- otu_info[,c(-1,-2)]
otu_info <- otu_info[,-17525]
#Order otu_info and metadata_info so they are the same order
otu_info <- otu_info[order(row.names(otu_info)),]
metadata_info <- metadata_info[order(row.names(metadata_info)),]
#Check if row names match
stopifnot(identical(row.names(metadata_info), row.names(otu_info)))
#Change row names
row.names(otu_info) <- metadata_info$new.Sample.ID
#Order by new row names
otu_info <- otu_info[order(row.names(otu_info)),]
#Transpose otu_info
trans_otu_info <- t(otu_info)
#Change Total OTU amounts into relative abundance
relabund_otu_info <- prop.table(trans_otu_info, margin=2)
#Calculate Spearman values
data_rcorr <- rcorr(as.matrix(trans_otu_info), type = "spearman")
spearman_cor <- data_rcorr$r
spearman_p <- data_rcorr$P
#Keep info interested in
subset_spearman_cor <- spearman_cor[1:23,24:46]
subset_spearman_p <- spearman_p[1:23,24:46]
#Correlation plot of FL samples against Bx samples
pdf("Fig5.pdf",height = 87/25.4, width=87/25.4)
par(mfrow=c(1,1), mar=c(0,0,0,0), cex=0.6)
corrplot((subset_spearman_cor),type="full", mar=c(0,0,0,0), cl.pos="b",
         p.mat=subset_spearman_p, sig.level = 0.05, insig="blank")
dev.off()
```

```
## quartz_off_screen
##                 2
```

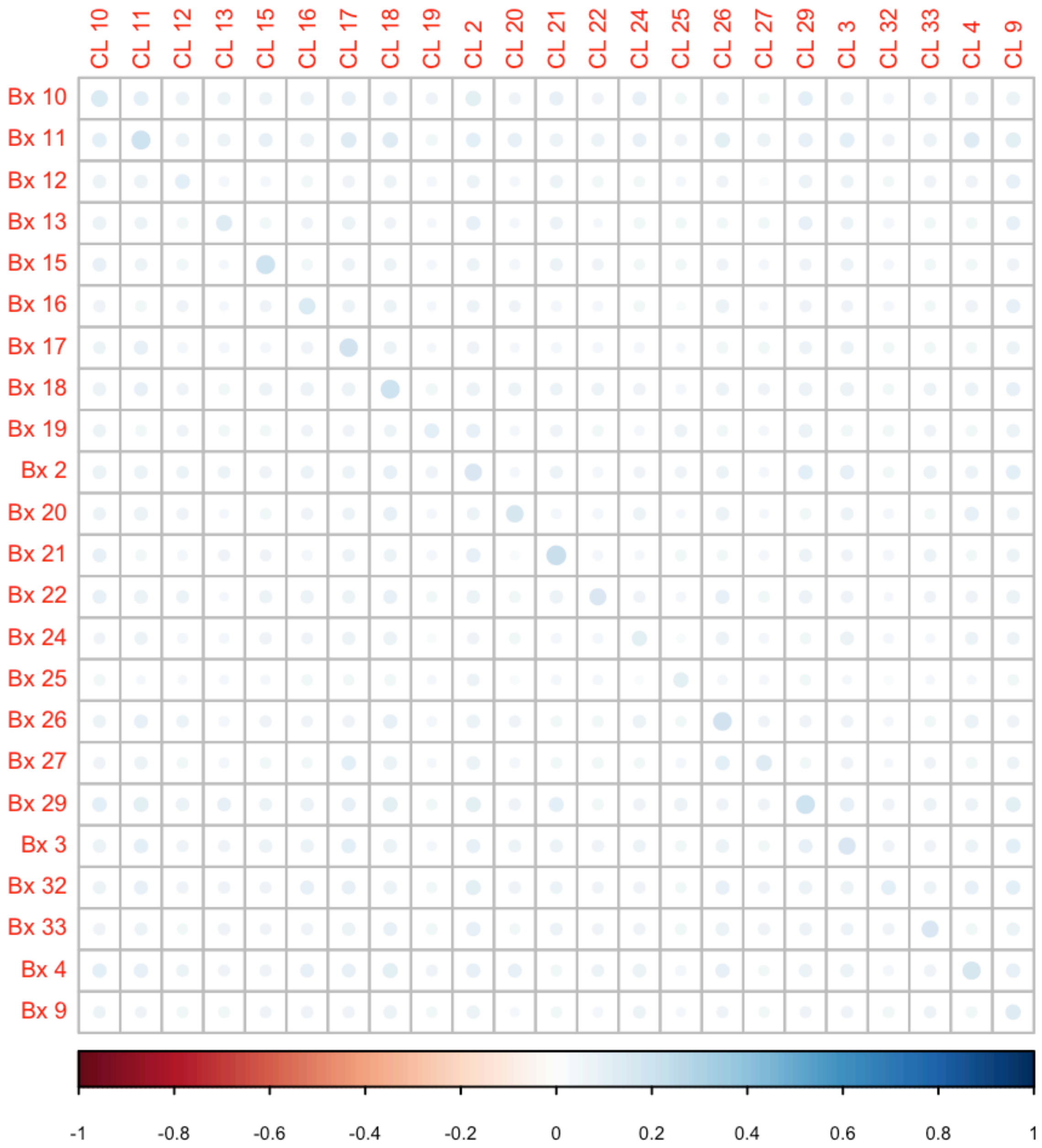

Figure 6: Differentially abundant genera between biopsy and lavage samples by LefSe

A) LEfSe LDA scores

```r
### Load packages or install if not present
if (!require("gplots")) {install.packages("gplots")
  library(gplots)}
if (!require("RColorBrewer")) {install.packages("RColorBrewer")
  library(RColorBrewer)}
#read in data
plot_data <- read.csv("16sBxCL_LEfSe_summary_subsample.txt", sep="\t")
plot_data$Species <- factor(plot_data$Species, levels=plot_data$Species[order(plot
_data$LDA)])
#Produce Plot
g <- ggplot(plot_data, aes(x=Species, y=LDA, fill=new.Class)) + geom_bar(stat="ide
ntity", position="identity") + coord_flip() + ylab("LDA SCORE (log 10)") + xlab(""
) + theme_set(theme_gray(base_size = 8)) + scale_fill_manual(name="Class", values
= c("#377EB8","#E41A1C"), breaks=c("Biopsy", "Lavage"), labels=c("Biopsy", "Lavage
")) + theme(text= element_text(size=8), axis.text.y  = element_text(size=8), axis.
text.x  = element_text(size=8), legend.text= element_text(size = 8), legend.key.si
ze = unit(4, "mm"), plot.margin=unit(c(1,1,1,1),"mm"))
ggsave("Fig6A.pdf", g, units="mm", height=70, width=170, dpi=300)
```

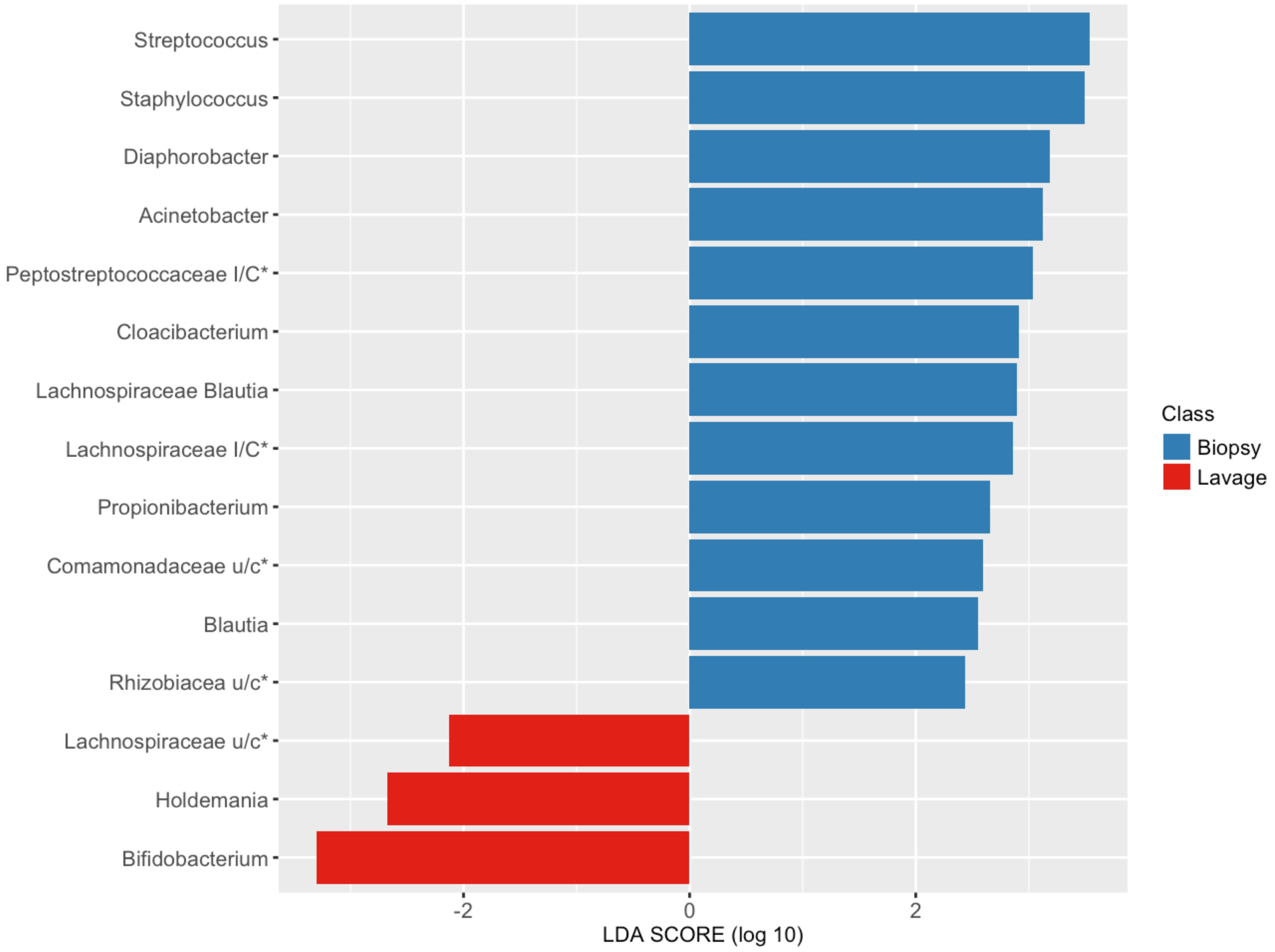

B) Heat map of Log2count of OTUs

```
### Load packages or install if not present
if (!require("arules")) {install.packages("arules")
  library(arules)}
if (!require("ade4")) {install.packages("ade4")
  library(ade4)}
if (!require("vegan")) {install.packages("vegan")
  library(vegan)}
if (!require("gdata")) {install.packages("gdata")
  library(gdata)}
if (!require("gplots")) {install.packages("gplots")
  library(gplots)}
if (!require("RColorBrewer")) {install.packages("RColorBrewer")
  library(RColorBrewer)}
#Load in data and edit
data <- read.csv("16sBxCL_LEfSe_subsample_otu_log2count_info_with_taxa.txt", sep="
\t", row.names=1)
metadata_info <- read.csv("16sBxCL_Metadata.txt", sep="\t", row.names=1)
heatmap_info <- data[,-1]
sample_info <- data$Sample_type
taxa_info <- read.csv("16sBxCL_LEfSe_subsample_otu_log2count_info_with_taxa.txt",
header=FALSE, sep="\t", row.names=1)
taxa_info <- taxa_info[1,-1]
#Selecting a colour palette to be used in plot
brewer_colours <- brewer.pal(9, "Set1")
#Order heatmap_info and metadata_info so they are the same order
heatmap_info <- heatmap_info[order(row.names(heatmap_info)),]
metadata_info <- metadata_info[order(row.names(metadata_info)),]
#Check if row names match
stopifnot(identical(row.names(metadata_info), row.names(heatmap_info)))
##Setting colours to sample_types
sample_type_colours <- gsub("FA",brewer_colours[1], sample_info)
sample_type_colours <- gsub("Bx", brewer_colours[2], sample_type_colours)
#Transposing heatmap_info
heatmap.info.t <- t(heatmap_info)
#produce heatmap
pdf("Fig6B.pdf",
    170/25.4, height=110/25.4)
heatmap.2(as.matrix(heatmap.info.t), ColSideColors=sample_type_colours, margins =
c(2.7,8.5), key.xlab="Log2count", key.title=NA, trace="none", col=function(x)rev(h
eat.colors(x)), labRow = row.names(heatmap.info.t), cexRow = 0.7, cexCol = 0.7, of
fsetRow=0.0000001, offsetCol = 0.0000001, labCol = metadata_info$new.Sample.ID, ke
y.par=list(mar=c(2,4,1,1), cex=0.4), lwid=c(1,5), lhei=c(1,5))
dev.off()
```

```
## quartz_off_screen
##                 2
```

```
## [1] TRUE
```

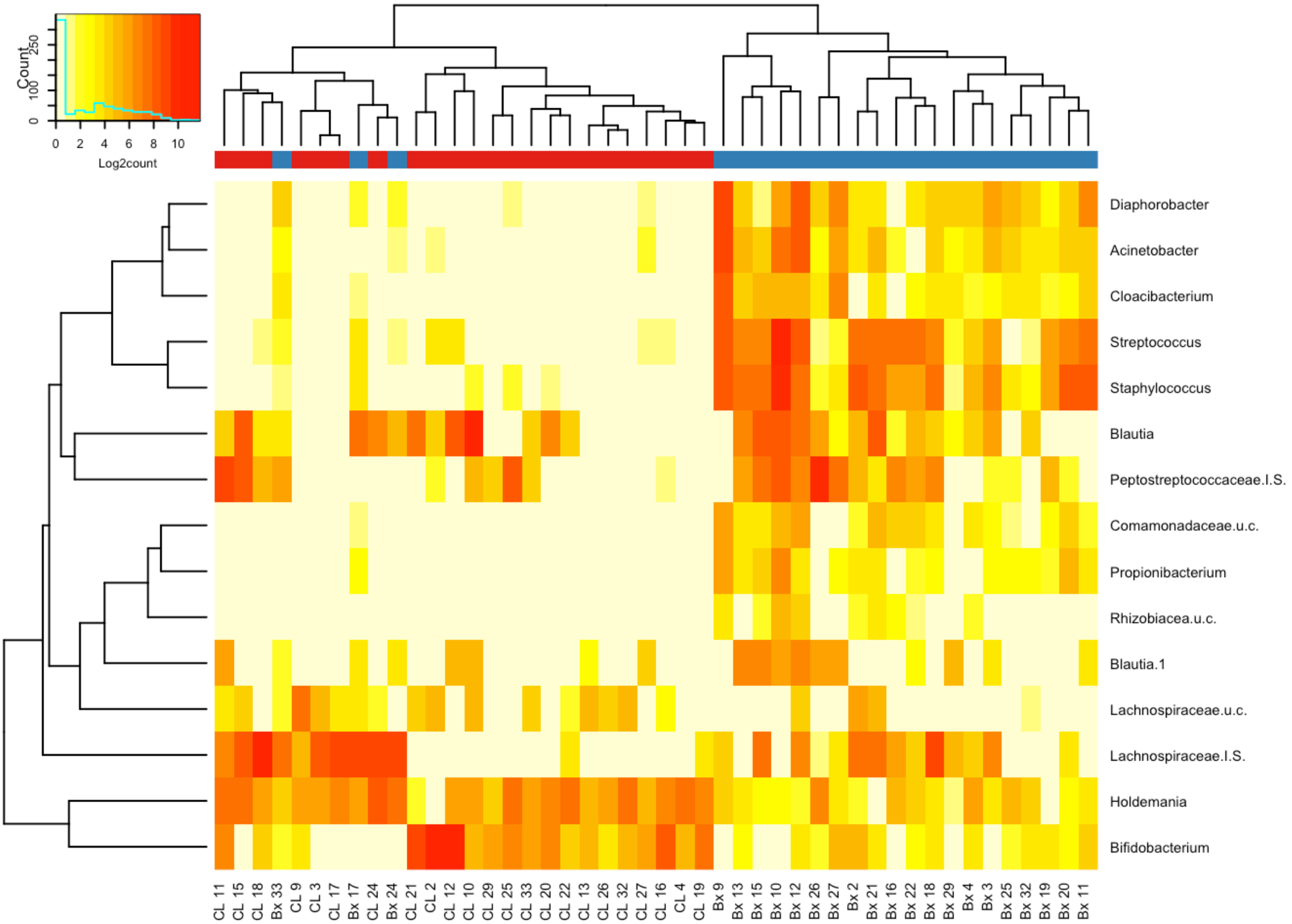

Figure 7: Heat map of Log2count of top 50 OTUs found to not be differentially abundant between biopsy and lavage samples by LefSe

```
### Load packages or install if not present
if (!require("arules")) {install.packages("arules")
  library(arules)}
if (!require("ade4")) {install.packages("ade4")
  library(ade4)}
if (!require("vegan")) {install.packages("vegan")
  library(vegan)}
if (!require("gdata")) {install.packages("gdata")
  library(gdata)}
if (!require("gplots")) {install.packages("gplots")
  library(gplots)}
if (!require("RColorBrewer")) {install.packages("RColorBrewer")
  library(RColorBrewer)}
#Read in data
data <- read.csv("16sBxCL_Top_50_OTU_log2count_info_minus_differentially_abundant_
otus_lefse_subsample.txt", sep="\t", row.names=1)
metadata_info <- read.csv("16sBxCL_Metadata.txt", sep="\t", row.names=1)
heatmap_info <- data[,-1]
sample_info <- data$Sample_type
taxa_info <- read.csv("16sBxCL_Top_50_OTU_taxa_info_minus_differentially_abundant_
otus_lefse_subsample.txt", sep="\t", row.names=1)
#Selecting colour palette for plot
brewer_colours <- brewer.pal(9, "Set1")
#Order heatmap_info and metadata_info so they are the same order
heatmap_info <- heatmap_info[order(row.names(heatmap_info)),]
metadata_info <- metadata_info[order(row.names(metadata_info)),]
#Check if row names match
stopifnot(identical(row.names(metadata_info), row.names(heatmap_info)))
##Set colours to sample_types
sample_type_colours <- gsub("FA",brewer_colours[1], sample_info)
sample_type_colours <- gsub("Bx", brewer_colours[2], sample_type_colours)
#Transposing heatmap_info
heatmap.info.t <- t(heatmap_info)
#Produce heatmap
pdf("Fig7.pdf", 180/25.4, height=110/25.4)
heatmap.2(as.matrix(heatmap.info.t), ColSideColors=as.character(metadata_info$Colo
ur), labRow = taxa_info$Taxa.ID, labCol = metadata_info$new.Sample.ID, margins = c
(2,7.5), key.xlab="Log2count", key.title=NA, trace="none", col=function(x)rev(heat
.colors(x)), cexRow = 0.65, cexCol = 0.6, offsetRow=0.0000001, offsetCol = 0.00000
01, key.par=list(mar=c(1,3,1,1), cex=0.3), lwid=c(1,5), lhei=c(1,5))
dev.off()
```

```
## quartz_off_screen
##                 2
```

```
## [1] TRUE
```

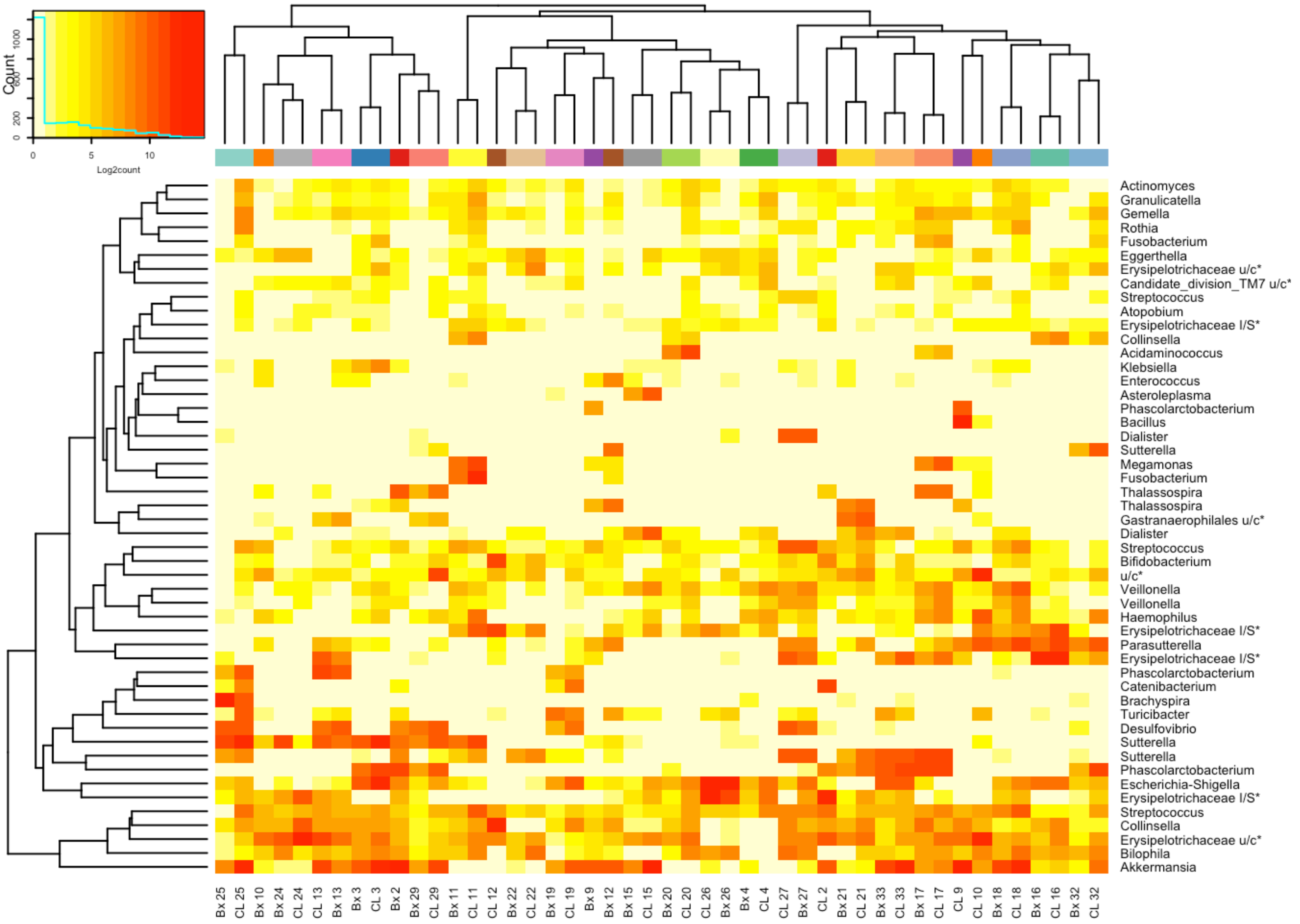

Figure 8: Clustering of samples' PICRUSt predicted KEGG pathways according to sample type (colonic biopsy and lavage) by NMDS, based on Yue & Clayton similarity distance.

```
#Input file names
axes_file_name <- "16sBxCL.KEGG_pathways.shared.thetayc.unique.lt.ave.nmds.axes"
metadata_file_name <- "16sBxCL_Metadata.txt"
#PDF output file name
PDF_file_name <- "Fig8.pdf"
##Relevant metadata columns
PAIRED <- "new.Sample.type"
SAMPLE <- "Individual"
#Read in data
plot_data <- read.csv(axes_file_name,sep="\t", row.names = 1)
metadata <- read.csv(metadata_file_name,sep="\t", row.names = 1)
#Order plot_data and metadata so they are the same order
plot_data <- plot_data[order(row.names(plot_data)),]
metadata <- metadata[order(row.names(metadata)),]
#Check if row names match and stop if not
stopifnot(identical(row.names(plot_data), row.names(metadata)))
#Merge data frames
plot_data_metadata <- merge(x=plot_data, y=metadata, by="row.names")
```

```
#Fix rownames after merge
row.names(plot_data_metadata) <- plot_data_metadata[,1]
plot_data_metadata <- plot_data_metadata[,-1]
#Sets column to variables
axis1 <- plot_data_metadata[,1]
axis2 <- plot_data_metadata[,2]
axis3 <- plot_data_metadata[,3]
#Set the minimum and maximum x so each plot is the same size as each other for com
parison
maxx <- max(c(max(axis1),max(axis2),max(axis3)))
minx <- min(c(min(axis1),min(axis2),min(axis3)))
#Grouping to colour by
group_samples <- as.factor(plot_data_metadata[,SAMPLE])
#Creates plot points for first and second samples
#This data will be used to connect the points together
first_level <- levels(plot_data_metadata[,PAIRED])[1]
second_level <- levels(plot_data_metadata[,PAIRED])[2]
first_rows <- plot_data_metadata[plot_data_metadata[,PAIRED]==first_level,]
second_rows <- plot_data_metadata[plot_data_metadata[,PAIRED]==second_level,]
#Order data to get colouring correct
first_rows <- first_rows[order(first_rows[,SAMPLE]),]
second_rows <- second_rows[order(second_rows[,SAMPLE]),]
first_group_samples <- as.factor(first_rows[,SAMPLE])
second_group_samples <- as.factor(second_rows[,SAMPLE])
#Selecting colour palette for plot
col.brew <- c(brewer.pal(9, "Set1"),brewer.pal(8,"Set2"),brewer.pal(12,"Set3"))
palette(col.brew)
#Assignment of axes labels
axis_1_lab <- paste("axis1")
axis_2_lab <- paste("axis2")
axis_3_lab <- paste("axis3")
#Function to produce single 2d plot
plot_production <- function(nx, ny, xlabel, ylabel){
  #Make an empty plotting area with axis labels
  plot(0,0, xlab = xlabel, ylab = ylabel, xlim=c(minx-0.001, maxx+0.001), ylim=c(m
inx-0.001, maxx+0.001), pch=19,col=NA, type='b')
  #Sets colour of background of plot
  rect(par("usr")[1], par("usr")[3], par("usr")[2], par("usr")[4], col = "#f6f6f6"
)
  #Sets gridlines that match the ticks of the axes
  grid (NULL,NULL, lty = 6, col = "black", lwd=0.3)
  #Add black borders to points
  points(first_rows[,nx], first_rows[,ny], type='p', pch=2, col="black", cex=0.8)
  points(second_rows[,nx], second_rows[,ny], type='p', pch=1, col="black", cex=0.8
)
  points(first_rows[,nx], first_rows[,ny], type='p', pch=2, col="black", cex=0.6)
  points(second_rows[,nx], second_rows[,ny], type='p', pch=1, col="black", cex=0.6
)
  #This part will plot pair by pair
  #This is so pairs only connect to each other by the dashed lines
  for (i in 1:num_samples){
    n <- i
    points(c(first_rows[n,nx],second_rows[n,nx]), c(first_rows[n,ny],second_rows[n
,ny]), type='b', pch=c(2,1), lwd=0.7, col=i, cex=0.7, lty=2)
```

```
  }
}
#Save plot to pdf file
pdf(PDF_file_name, width = 85/25.4, height = 85/25.4)
#Creates diagram to put NMDs plots into
par(mfrow=c(2,2), oma = c(0.8,0.8,0.8,0.8), mgp= c(2,1,0), mar=c(3,3,1,1), cex=0.5
, lwd=0.7)
#Variable used within function
num_samples <- nrow(first_rows)
# creating the 3 2d plots
plot_production(1,2,axis_1_lab,axis_2_lab)
plot_production(1,3,axis_1_lab,axis_3_lab)
plot_production(3,2,axis_3_lab,axis_2_lab)
#number for plotting legend
#n is the number of samples there are
n <- nlevels(group_samples)
#Determine if there is an even or odd amount of paired samples
if (n %% 2 == 0) { n1 <- n/2
n2 <- n/2 } else { n1 <- (n+1)/2
n2 <- (n-1)/2
}
x_leg <- c(rep(1, n1),rep(3, n2))
y_leg <- c((n1+1):2,(n2+1):2)
#Plot used as a legend for the overall diagram
plot(x_leg, y_leg, xlab="", ylab="", xlim=c(0,5), ylim=c(0,(n/2)+2), pch=19, col="
black",cex=1.3, xaxt='n', yaxt='n')
points(x_leg, y_leg, col=col.brew, pch=19)
text(x_leg+1,y_leg,levels(group_samples))
points(1,1, lwd=0.5, pch=2)
text(2,1, levels(plot_data_metadata[,PAIRED])[1])
points(3,1, lwd=0.5, pch=1)
text(4,1, levels(plot_data_metadata[,PAIRED])[2])
dev.off()
```

```
## quartz_off_screen
##                 2
```

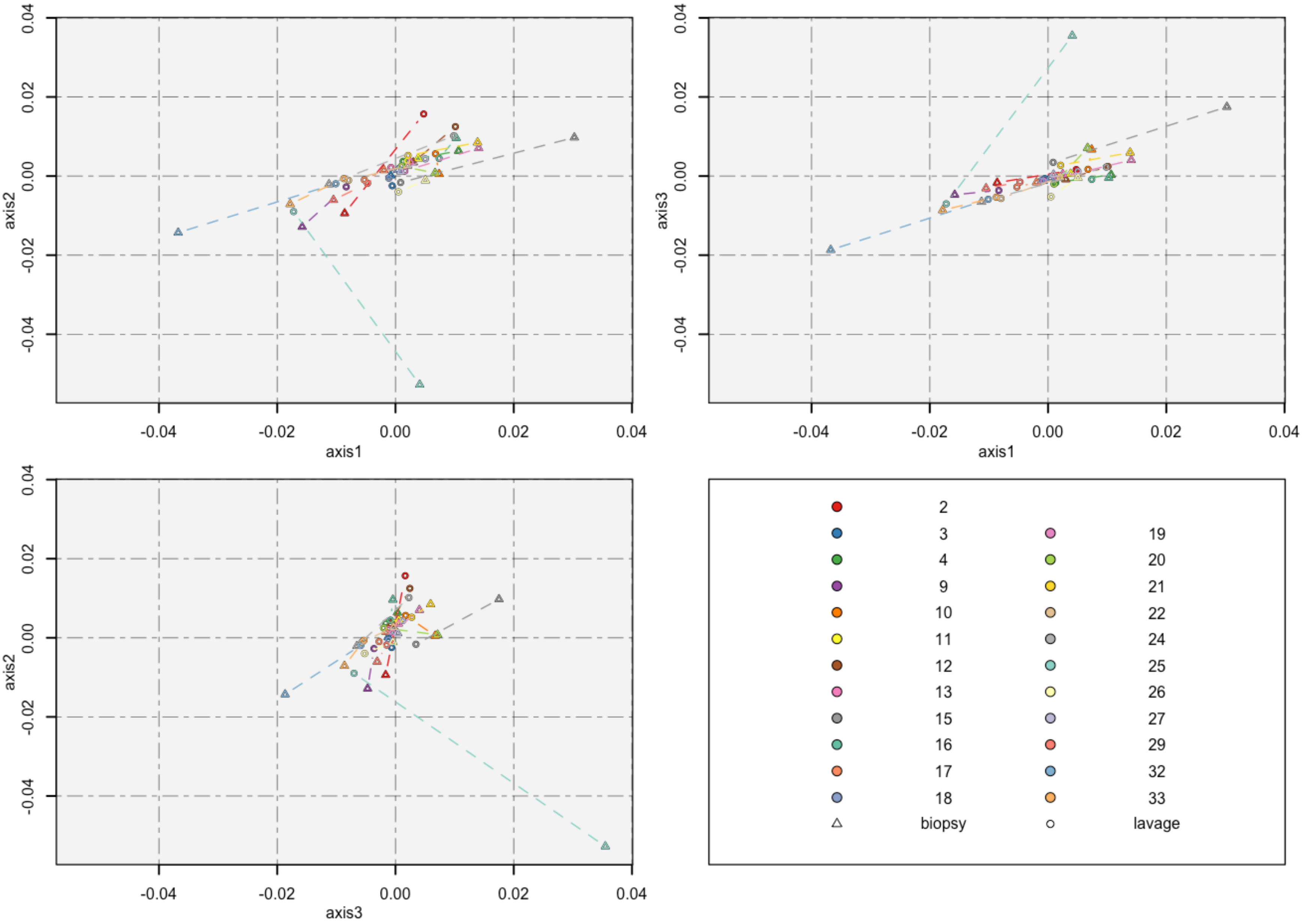

Supplementary figure 2: Rarefaction curve of all 46 samples following removal of rare OTUs

```r
### Load packages or install if not present
if (!require("RColorBrewer")) {install.packages("RColorBrewer")
  library(RColorBrewer)}
if (!require("ggplot2")) {install.packages("ggplot2")
  library(ggplot2)}
if (!require("tidyr")) {install.packages("tidyr")
  library(tidyr)}
#Set name of input file
rarefaction_file="colonoscopy.makecontigsfile.trim.contigs.good.unique.good.filter
.unique.precluster.pick.pick.an.unique_list.0.03.pick.groups.rarefaction"
#Name of output PDF file
pdf_file_name="SuppFig2.pdf"
#Read in data
rarefaction_data <- read.csv(file = rarefaction_file, sep="\t")
#Remove confidence interval information
rarefaction_data <- rarefaction_data[,c(1,seq(from=2, to= ncol(rarefaction_data),
by=3))]
#Convert to long list format
rarefaction_data_long <- gather(rarefaction_data, sample, Measure, 2:ncol(rarefact
ion_data))
#Change name of numsampled to match ggplot
colnames(rarefaction_data_long)[1] <- "numsampled"
#Select colour palette to be used in plot
colset <- c(brewer.pal(8, "Set1"), brewer.pal(8, "Set2"), brewer.pal(12, "Set3"),
brewer.pal(9, "Pastel1"))
#ggplot for rarefaction curve
g <- ggplot(data = rarefaction_data_long, aes(x=numsampled, y=Measure, group=sampl
e, colour=sample)) + geom_smooth(se = FALSE, size=0.2) + theme_set(theme_gray(base
_size = 8)) + theme(text=element_text(size=8), legend.position="none", plot.margin
=unit(c(0,0,0,0),"mm")) + scale_y_continuous(name="OTUs found") + scale_x_continuo
us(name="Number sampled")
ggsave(pdf_file_name, g, units="mm", width=85, height=85)
```

```
## Warning: Removed 18554 rows containing non-finite values (stat_smooth).
```

```
## Warning: Removed 18554 rows containing non-finite values (stat_smooth).
```

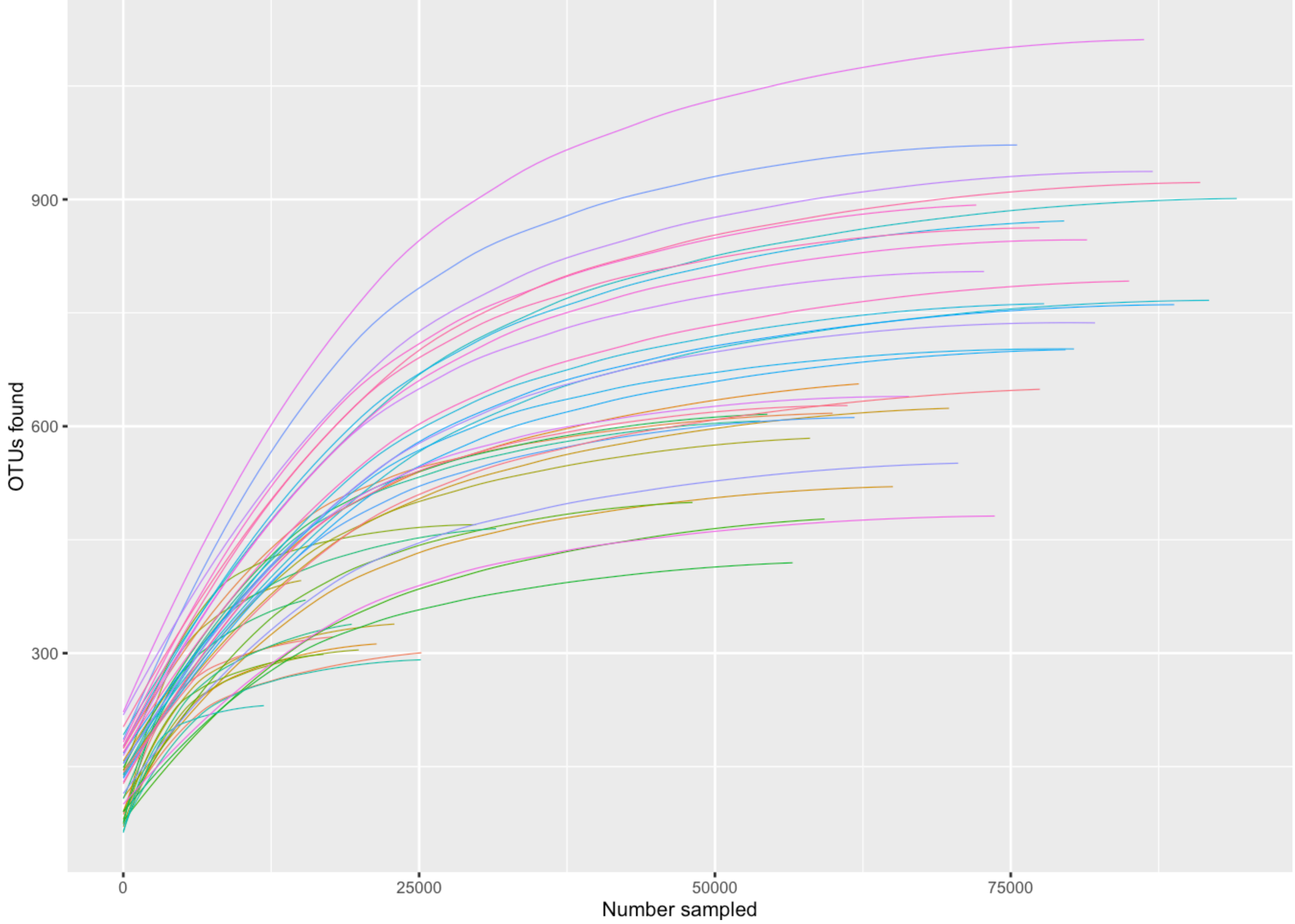

Supplementary figure 4: Assessment of the uncertainty in the hierarchical cluster analysis

```
##(A) log2 count of sequences within families
### Load packages or install if not present
if (!require("pvclust")) {install.packages("pvclust")
  library(pvclust)}
```

```
## Loading required package: pvclust
```

```
#Load in data
data <- read.csv("16sBxCL_family_phylotype_log2counts_summary.txt", sep="\t", row.
names=1)
metadata_info <- read.csv("16sBxCL_Metadata.txt", sep="\t", row.names=1)
#Remove unwanted column
heatmap_info <- data[,-1]
sample_info <- metadata_info$Sample.type
#Order heatmap_info and metadata so they are the same order
heatmap_info <- heatmap_info[order(row.names(heatmap_info)),]
metadata_info <- metadata_info[order(row.names(metadata_info)),]
#Check if row names match
stopifnot(identical(row.names(metadata_info), row.names(heatmap_info)))
#Transposing heatmap_info
heatmap.info.t <- t(heatmap_info)
#PVclust to test reliability of clustering by Sample
heatmap.info.t.new_names <- heatmap.info.t
colnames(heatmap.info.t.new_names) <- metadata_info$new.Sample.ID
#pvclust analysis
pvclust_samples <- pvclust(heatmap.info.t.new_names, method.dist="euclidean",metho
d.hclust = "complete", nboot=1000)
```

```
## Bootstrap (r = 0.5)... Done.
## Bootstrap (r = 0.6)... Done.
## Bootstrap (r = 0.69)... Done.
## Bootstrap (r = 0.8)... Done.
## Bootstrap (r = 0.9)... Done.
## Bootstrap (r = 1.0)... Done.
## Bootstrap (r = 1.09)... Done.
## Bootstrap (r = 1.2)... Done.
## Bootstrap (r = 1.3)... Done.
## Bootstrap (r = 1.39)... Done.
```

```
#produce PDF of pvclust hierarchical assessment
pdf("SuppFig4A.pdf", width = 85/25.4, height = 80/25.4)
par(mar=c(0.2,3,2,0), cex = 0.6)
#Plot pvclust data
plot(pvclust_samples,
     #Change text size of labels and p-values
     cex = 0.7, cex.pv = 0.7)
#Add boxs to signify significant clusters
pvrect(pvclust_samples, alpha = 0.95)
dev.off()
```

```
## quartz_off_screen
##                 2
```

```
###
##(B) Differentially abundant OTUs between biopsy and lavage samples by LefSe
###
#Load in data and edit
data <- read.csv("16sBxCL_LEfSe_subsample_otu_log2count_info_with_taxa.txt", sep="
\t", row.names=1)
metadata_info <- read.csv("16sBxCL_Metadata.txt", sep="\t", row.names=1)
heatmap_info <- data[,-1]
sample_info <- data$Sample_type
taxa_info <- read.csv("16sBxCL_LEfSe_subsample_otu_log2count_info_with_taxa.txt",
header=FALSE, sep="\t", row.names=1)
taxa_info <- taxa_info[1,-1]
#Order heatmap_info and metadata_info so they are the same order
heatmap_info <- heatmap_info[order(row.names(heatmap_info)),]
metadata_info <- metadata_info[order(row.names(metadata_info)),]
#Check if row names match
stopifnot(identical(row.names(metadata_info), row.names(heatmap_info)))
#Transposing heatmap_info
heatmap.info.t <- t(heatmap_info)
#PVclust to test reliability of clustering by Sample
heatmap.info.t.new_names <- heatmap.info.t
colnames(heatmap.info.t.new_names) <- metadata_info$new.Sample.ID
#pvclust analysis
pvclust_samples <- pvclust(heatmap.info.t.new_names, method.dist="euclidean",metho
d.hclust = "complete", nboot=1000)
```

```
## Bootstrap (r = 0.47)... Done.
## Bootstrap (r = 0.6)... Done.
## Bootstrap (r = 0.67)... Done.
## Bootstrap (r = 0.8)... Done.
## Bootstrap (r = 0.87)... Done.
## Bootstrap (r = 1.0)... Done.
## Bootstrap (r = 1.07)... Done.
## Bootstrap (r = 1.2)... Done.
## Bootstrap (r = 1.27)... Done.
## Bootstrap (r = 1.4)... Done.
```

```
#produce PDF of pvclust hierarchical assessment
pdf("SuppFig4B.pdf", width = 85/25.4, height = 80/25.4)
par(mar=c(0.2,3,2,0), cex = 0.6)
plot(pvclust_samples,
     #Change text size of labels and p-values
     cex = 0.7, cex.pv = 0.7)
#Add boxs to signify significant clusters
pvrect(pvclust_samples, alpha = 0.95)
dev.off()
```

```
## quartz_off_screen
##                 2
```

```
###
##(C) The top 50 OTUs found to not be differentially abundant between biopsy and l
avage samples by LEfSe
###
#Read in data
data <- read.csv("16sBxCL_Top_50_OTU_log2count_info_minus_differentially_abundant_
otus_lefse_subsample.txt", sep="\t", row.names=1)
metadata_info <- read.csv("16sBxCL_Metadata.txt", sep="\t", row.names=1)
heatmap_info <- data[,-1]
sample_info <- data$Sample_type
taxa_info <- read.csv("16sBxCL_Top_50_OTU_taxa_info_minus_differentially_abundant_
otus_lefse_subsample.txt", sep="\t", row.names=1)
#Order heatmap_info and metadata_info so they are the same order
heatmap_info <- heatmap_info[order(row.names(heatmap_info)),]
metadata_info <- metadata_info[order(row.names(metadata_info)),]
#Check if row names match
stopifnot(identical(row.names(metadata_info), row.names(heatmap_info)))
#Transposing heatmap_info
heatmap.info.t <- t(heatmap_info)
#PVclust to test reliability of clustering by Sample
heatmap.info.t.new_names <- heatmap.info.t
colnames(heatmap.info.t.new_names) <- metadata_info$new.Sample.ID
#pvclust analysis
pvclust_samples <- pvclust(heatmap.info.t.new_names, method.dist="euclidean",metho
d.hclust = "complete", nboot=1000)
```

```
## Bootstrap (r = 0.5)... Done.
## Bootstrap (r = 0.6)... Done.
## Bootstrap (r = 0.7)... Done.
## Bootstrap (r = 0.8)... Done.
## Bootstrap (r = 0.9)... Done.
## Bootstrap (r = 1.0)... Done.
## Bootstrap (r = 1.1)... Done.
## Bootstrap (r = 1.2)... Done.
## Bootstrap (r = 1.3)... Done.
## Bootstrap (r = 1.4)... Done.
```

```
#produce PDF of pvclust hierarchical assessment
pdf("SuppFig4C.pdf", width = 85/25.4, height = 80/25.4)
par(mar=c(0.2,3,2,0), cex = 0.6)
plot(pvclust_samples,
     #Change text size of labels and p-values
     cex = 0.7, cex.pv = 0.7)
#Add boxs to signify significant clusters
pvrect(pvclust_samples, alpha = 0.95)
dev.off()
```

```
## quartz_off_screen
##                 2
```

```
## Bootstrap (r = 0.5)... Done.
## Bootstrap (r = 0.6)... Done.
## Bootstrap (r = 0.69)... Done.
## Bootstrap (r = 0.8)... Done.
## Bootstrap (r = 0.9)... Done.
## Bootstrap (r = 1.0)... Done.
## Bootstrap (r = 1.09)... Done.
## Bootstrap (r = 1.2)... Done.
## Bootstrap (r = 1.3)... Done.
## Bootstrap (r = 1.39)... Done.
```

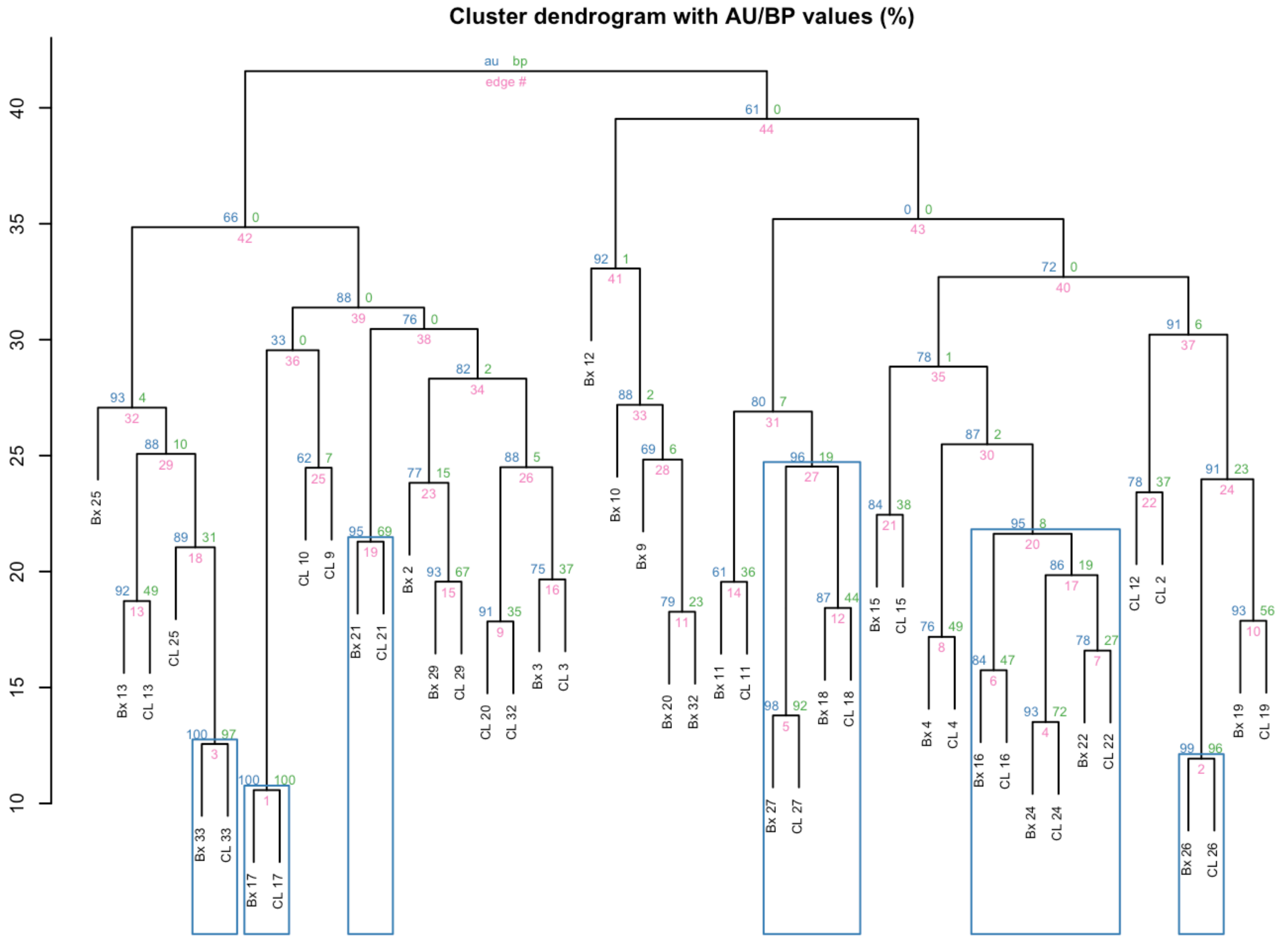


```
## Bootstrap (r = 0.47)... Done.
## Bootstrap (r = 0.6)... Done.
## Bootstrap (r = 0.67)... Done.
## Bootstrap (r = 0.8)... Done.
## Bootstrap (r = 0.87)... Done.
## Bootstrap (r = 1.0)... Done.
## Bootstrap (r = 1.07)... Done.
## Bootstrap (r = 1.2)... Done.
## Bootstrap (r = 1.27)... Done.
## Bootstrap (r = 1.4)... Done.
```

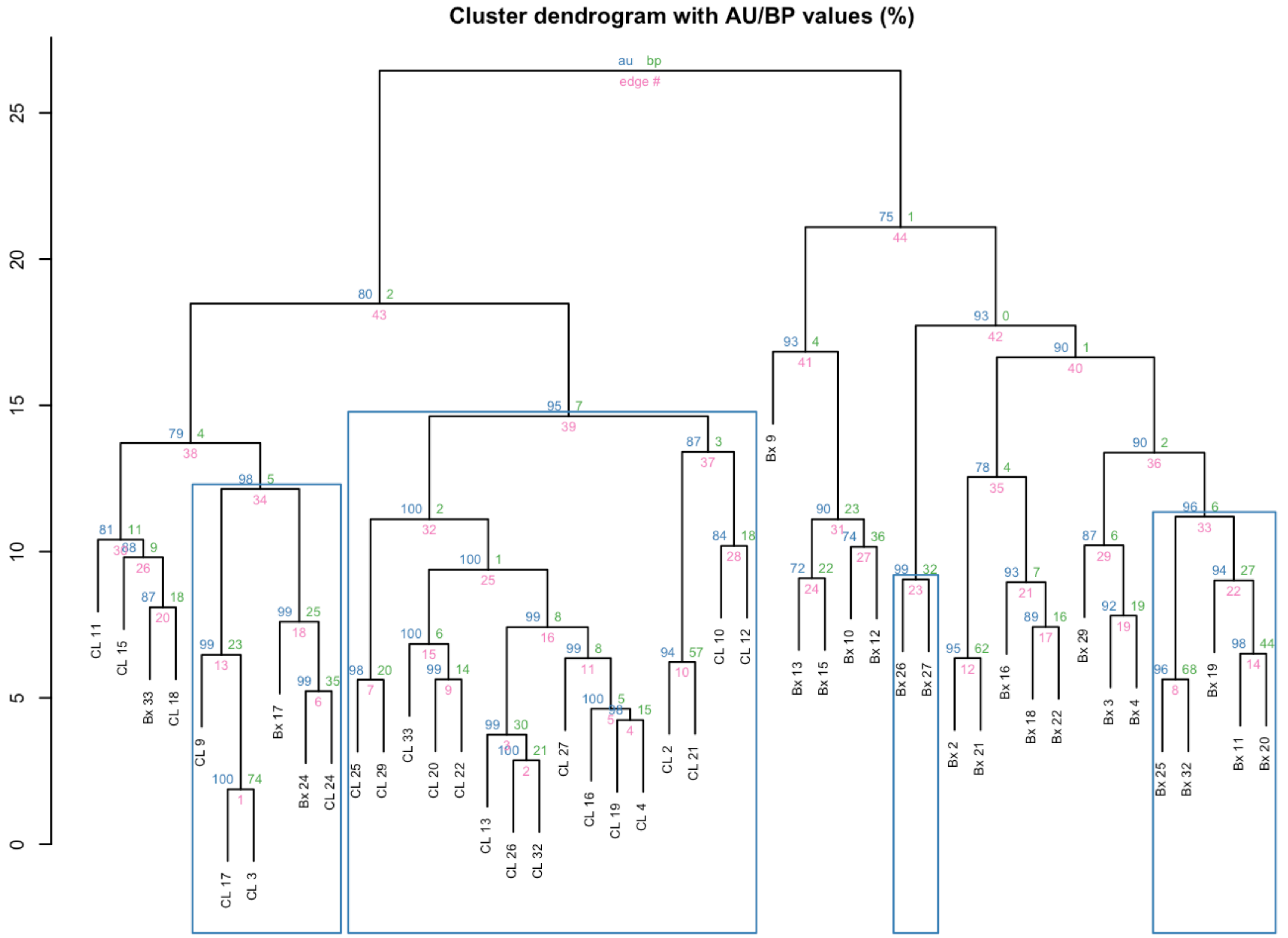


```
## Bootstrap (r = 0.5)... Done.
## Bootstrap (r = 0.6)... Done.
## Bootstrap (r = 0.7)... Done.
## Bootstrap (r = 0.8)... Done.
## Bootstrap (r = 0.9)... Done.
## Bootstrap (r = 1.0)... Done.
## Bootstrap (r = 1.1)... Done.
## Bootstrap (r = 1.2)... Done.
## Bootstrap (r = 1.3)... Done.
## Bootstrap (r = 1.4)... Done.
```

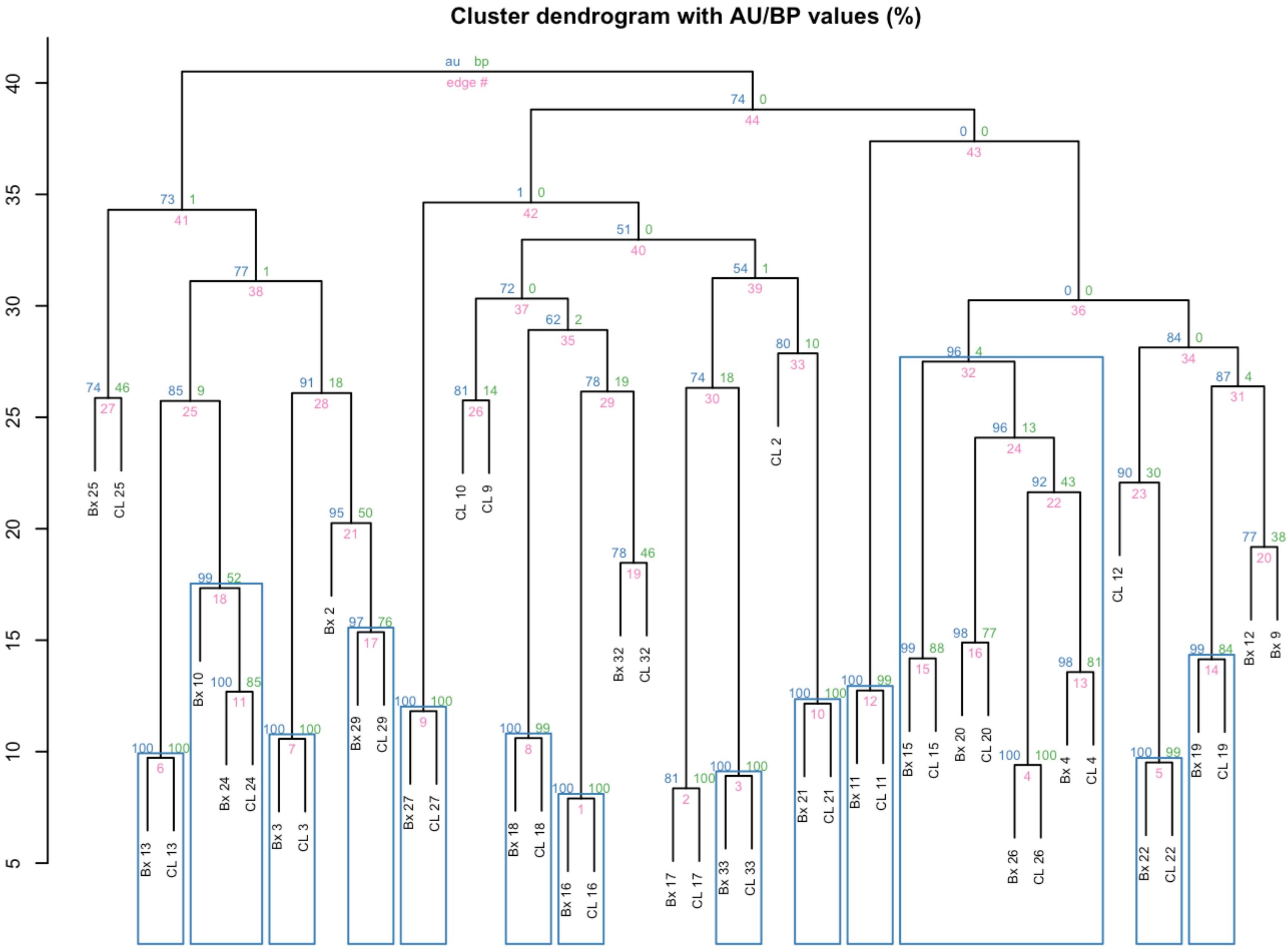

Supplementary figure 5: Predicted KEGG pathway comparison between colonic biopsy and lavage sample

A) KEGG pathway abundance

```
### Load packages or install if not present
if (!require("RColorBrewer")) {install.packages("RColorBrewer")
  library(RColorBrewer)}
if (!require("ggplot2")) {install.packages("ggplot2")
  library(ggplot2)}
if (!require("tidyr")) {install.packages("tidyr")
  library(tidyr)}
#File names
taxa_table_file <- "16sBxCL_picrust_predicted_metagenome.KEGG_pathways_L3.relabund
.txt"
metadata_file <- "16sBxCL_Metadata.txt"
PDF_file <- "SuppFig5A.pdf"
#Read in files
taxa_data <- read.csv(taxa_table_file, sep = "\t", row.names = 1)
metadata <- read.csv(metadata_file, sep = "\t", row.names = 1)
#Remove empty rows
taxa.no0 <- taxa_data[ rowSums(taxa_data)!=0,]
#Transpose taxa data
taxa.no0.t <- t(taxa.no0)
#Check if heatmap_info and metadata_info are in the same order
taxa.no0.t <- taxa.no0.t[order(row.names(taxa.no0.t)),]
metadata <- metadata[order(row.names(metadata)),]
#Check if row names match
stopifnot(identical(row.names(taxa.no0.t), row.names(metadata)))
#Merge data
plot_data_metadata <- merge(x=taxa.no0.t, y=metadata, by="row.names")
#Change data to long list format
plot_data_metadata_long <- gather(plot_data_metadata, Taxa, Measure, (2:281))
#Select colour palette for plot
colset <- rep (c(brewer.pal(8, "Set1"), brewer.pal(8, "Set2"), brewer.pal(12, "Set
3"), brewer.pal(9, "Pastel1")), 10)
#Plot the data
g_bar <- ggplot(plot_data_metadata_long, aes(x =new.Sample.type, y=Measure, fill=T
axa)) + geom_bar(stat = 'identity', position = 'fill', width=0.95) + facet_wrap( ~
Individual, nrow=1) + theme_set(theme_grey(base_size = 8)) + theme(legend.position
="none", panel.margin = unit(0.1, "lines"), axis.text.x = element_text(angle = 90,
hjust = 1)) + scale_fill_manual(values = colset) + xlab("Subject type") + ylab("Re
lative abundance") + scale_y_continuous(expand = c(0,0)) + geom_text(aes(x=1, y=1.
00, label="Stretch it"), vjust=-1)
ggsave(PDF_file, g_bar, units="mm", height=105, width=170, dpi=300)
```

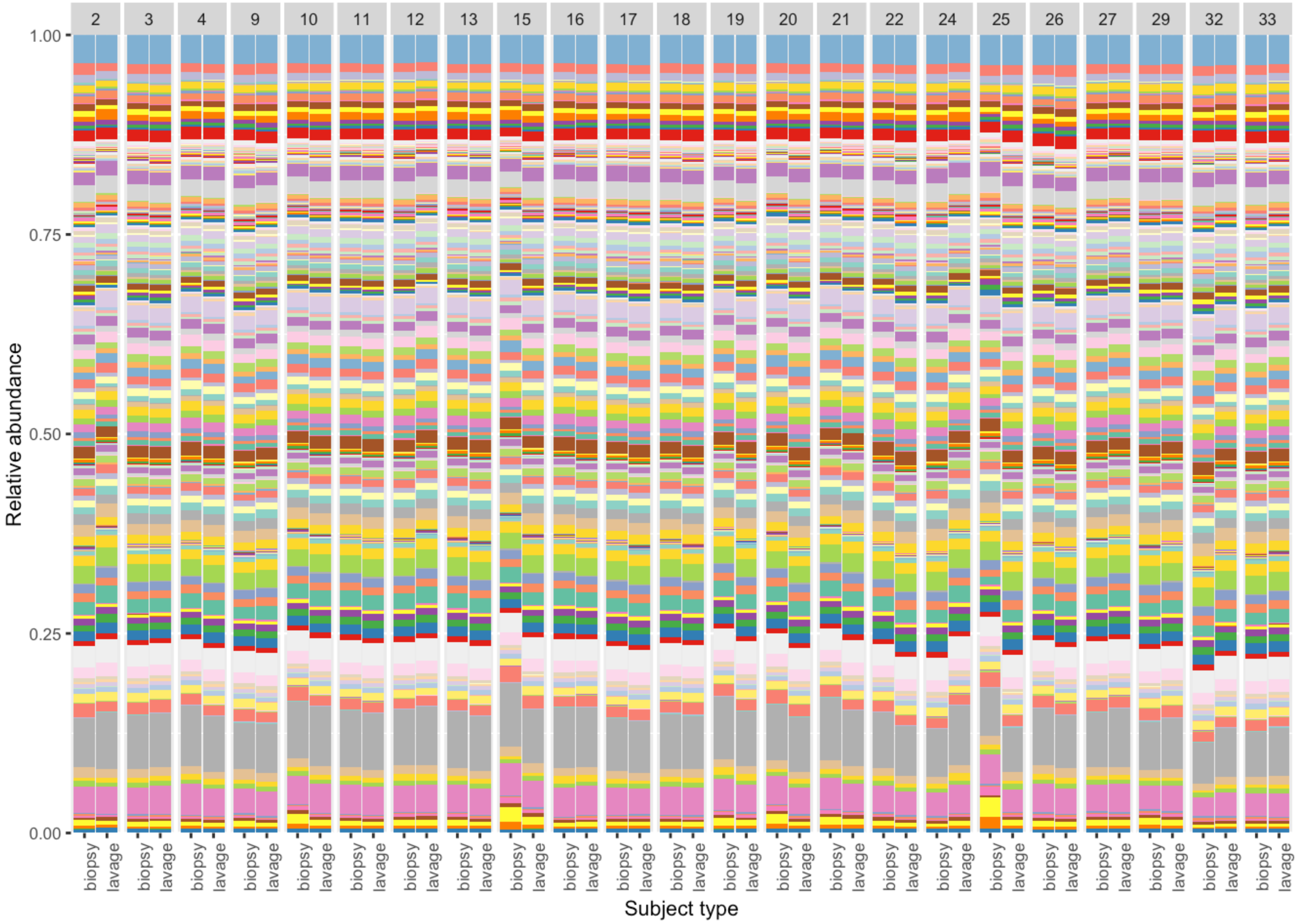

B) Alpha diversity scores

```
### Load packages or install if not present
if (!require("RColorBrewer")) {
  install.packages("RColorBrewer")
  library(RColorBrewer)}
if (!require("ggplot2")) {install.packages("ggplot2")
  library(ggplot2)}
if (!require("tidyr")) {install.packages("tidyr")
  library(tidyr)}
#Name of input files
alpha_file="16sBxCL.KEGG_pathways.shared.groups.ave-std.summary"
metadata_file="16sBxCL_Metadata.txt"
#name of output PDF file
pdf_file_box_plot="SuppFig5B.pdf"
#Read in data and manipulate
alpha_data <- read.csv(alpha_file,sep="\t")
alpha_data <- alpha_data[alpha_data$method == "ave",]
rownames(alpha_data) <- alpha_data$group
#Keep relevant columns
alpha_data <- alpha_data[,c("sobs", "chao", "shannon", "invsimpson", "coverage")]
#Rename columns
colnames(alpha_data) <- c("KEGG Pathways", "Chao","Shannon-Weiner", "Inverse Simps
on", "Coverage")
#To determine order of alpha diversity for plot
alpha_order <- c("KEGG Pathways", "Chao","Shannon-Weiner", "Inverse Simpson", "Cov
```

```
erage")
#Function to manipulate data
produce_plot <- function(ALPHA_DATA, METADATA_FILE, ORDER_ALPHA){
### Load package or install if not present
if (!require("tidyr")) {install.packages("tidyr")
    library(tidyr)}
#Read in Metadata file
metadata <- read.csv(METADATA_FILE,sep="\t", row.names = 1)
#Order plot_data and metadata so they are the same order
alpha_data_t <- ALPHA_DATA[order(row.names(ALPHA_DATA)),]
metadata <- metadata[order(row.names(metadata)),]
#Check if row names match and stop if not
stopifnot(identical(row.names(alpha_data_t), row.names(metadata)))
#Merge data frames
plot_data_metadata <- merge(x=alpha_data_t, y=metadata, by="row.names")
#Fix rownames after merge
row.names(plot_data_metadata) <- plot_data_metadata[,1]
colnames(plot_data_metadata)[1] <- "Sample_ID"
#Convert to long
alpha_long <- gather(plot_data_metadata, Alpha_diversity_measure, value, 2:(ncol(A
LPHA_DATA)+1))
#Reorder measures
alpha_long$Alpha_diversity_measure_f <- factor(alpha_long$Alpha_diversity_measure,
levels=ORDER_ALPHA)
return(alpha_long)
}
#Function to manipulate data to make it ready for ggplot
alpha_long <- produce_plot(alpha_data, metadata_file, alpha_order)

#Mann whitney u test
alpha_long_t <- alpha_long[,-11]
mann_whitney_data <- spread(alpha_long_t, Alpha_diversity_measure_f, value)
colnames(mann_whitney_data)[13:16] <- c("KEGG", "Chao", "ShannonWeiner", "InvSimps
on")
wilcox.test(KEGG ~ new.Sample.type, data=mann_whitney_data)
```

```
##
##  Wilcoxon rank sum test
##
## data:  KEGG by new.Sample.type
## W = 405, p-value = 0.001632
## alternative hypothesis: true location shift is not equal to 0
```

```
wilcox.test(Chao ~ new.Sample.type, data=mann_whitney_data)
```

```
##
##  Wilcoxon rank sum test
##
## data:  Chao by new.Sample.type
## W = 441, p-value = 4.988e-05
## alternative hypothesis: true location shift is not equal to 0
```

```
wilcox.test(ShannonWeiner ~ new.Sample.type, data=mann_whitney_data)
```

```
##
##  Wilcoxon rank sum test
##
## data:  ShannonWeiner by new.Sample.type
## W = 256, p-value = 0.8618
## alternative hypothesis: true location shift is not equal to 0
```

```
wilcox.test(InvSimpson ~ new.Sample.type, data=mann_whitney_data)
```

```
##
##  Wilcoxon rank sum test
##
## data:  InvSimpson by new.Sample.type
## W = 253, p-value = 0.8109
## alternative hypothesis: true location shift is not equal to 0
```

```
wilcox.test(Coverage ~ new.Sample.type, data=mann_whitney_data)
```

```
## Warning in wilcox.test.default(x = c(0.999991, 0.99999, 0.999991,
## 0.999987, : cannot compute exact p-value with ties
```

```
##
##  Wilcoxon rank sum test with continuity correction
##
## data:  Coverage by new.Sample.type
## W = 87, p-value = 9.222e-05
## alternative hypothesis: true location shift is not equal to 0
```

```
#Select colour palette for plot
col.brew <- c(brewer.pal(9, "Set1"),brewer.pal(8,"Set2"),brewer.pal(12,"Set3"))
#Box Plot for alpha diversity comparing aspirate and biopsy
g_box <- ggplot(alpha_long, aes(x=Sample.type, y=value, fill=new.Sample.type)) +
geom_boxplot(outlier.colour = NA) + theme_set(theme_gray(base_size = 8)) + facet_w
rap(~ Alpha_diversity_measure_f, nrow=1, scales="free") + geom_point(position = po
sition_jitter(width = 0.2)) + scale_x_discrete(breaks=NULL, name="") + scale_fill_
manual(values=c(col.brew[2], col.brew[1]), name="Sample type") + scale_y_continuou
s(name="") + theme_grey(base_size = 8) + theme(legend.position="bottom", legend.ma
rgin=unit(0,"cm"))
ggsave(pdf_file_box_plot, g_box, units="mm", height=75, width=170, dpi=300)
```

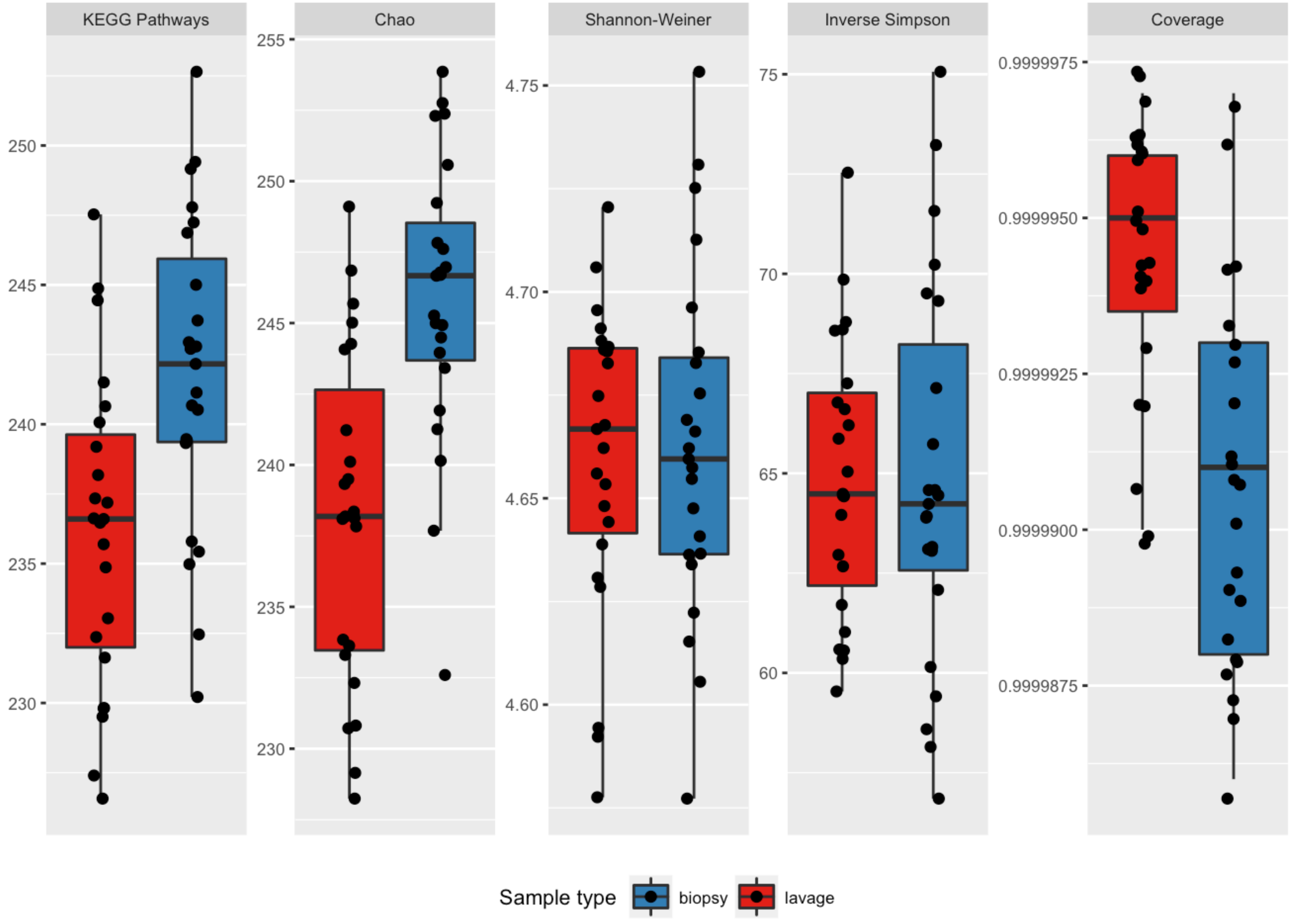

C) LEfSe LDA scores

```
### Load packages or install if not present
if (!require("gplots")) {install.packages("gplots")
  library(gplots)}
if (!require("RColorBrewer")) {install.packages("RColorBrewer")
  library(RColorBrewer)}
if (!require("ggplot2")) {install.packages("ggplot2")
  library(ggplot2)}
#read in data and manipulate
plot_data <- read.csv("16sBxCL_LEfSe_kegg_pathway_discovered_biomarkers.txt", sep=
"\t", header=FALSE)
```

```
## Warning in read.table(file = file, header = header, sep = sep,
## quote = quote, : incomplete final line found by readTableHeader on
## '16sBxCL_LEfSe_kegg_pathway_discovered_biomarkers.txt'
```

```
colnames(plot_data) <- c("KEGG", "LogMaxMean", "Class", "LDA", "pvalue", "new.Clas
s")
plot_data$KEGG <- factor(plot_data$KEGG, levels=plot_data$KEGG[order(plot_data$LDA
)])
#Produce plot
g <- ggplot(plot_data, aes(x=KEGG, y=LDA, fill=new.Class)) + geom_bar(stat="identi
ty", position="identity") + theme_set(theme_gray(base_size = 8)) + coord_flip() +
ylab("LDA SCORE (log 10)") + xlab("") + scale_fill_manual(name="Class", values = c
("#377EB8","#E41A1C"), breaks=c("Biopsy", "Lavage"), labels=c("Biopsy", "Lavage"))
+ theme(text= element_text(size=8), axis.text.y  = element_text(size=8), axis.text
.x  = element_text(size=8), legend.text= element_text(size = 8), legend.key.size =
unit(4, "mm"), plot.margin=unit(c(1,1,1,1),"mm"))
ggsave("SuppFig5C.pdf", g, units="mm", height=50, width=170, dpi=300)
```

```
## Warning in read.table(file = file, header = header, sep = sep,
## quote = quote, : incomplete final line found by readTableHeader on
## '16sBxCL_LEfSe_kegg_pathway_discovered_biomarkers.txt'
```

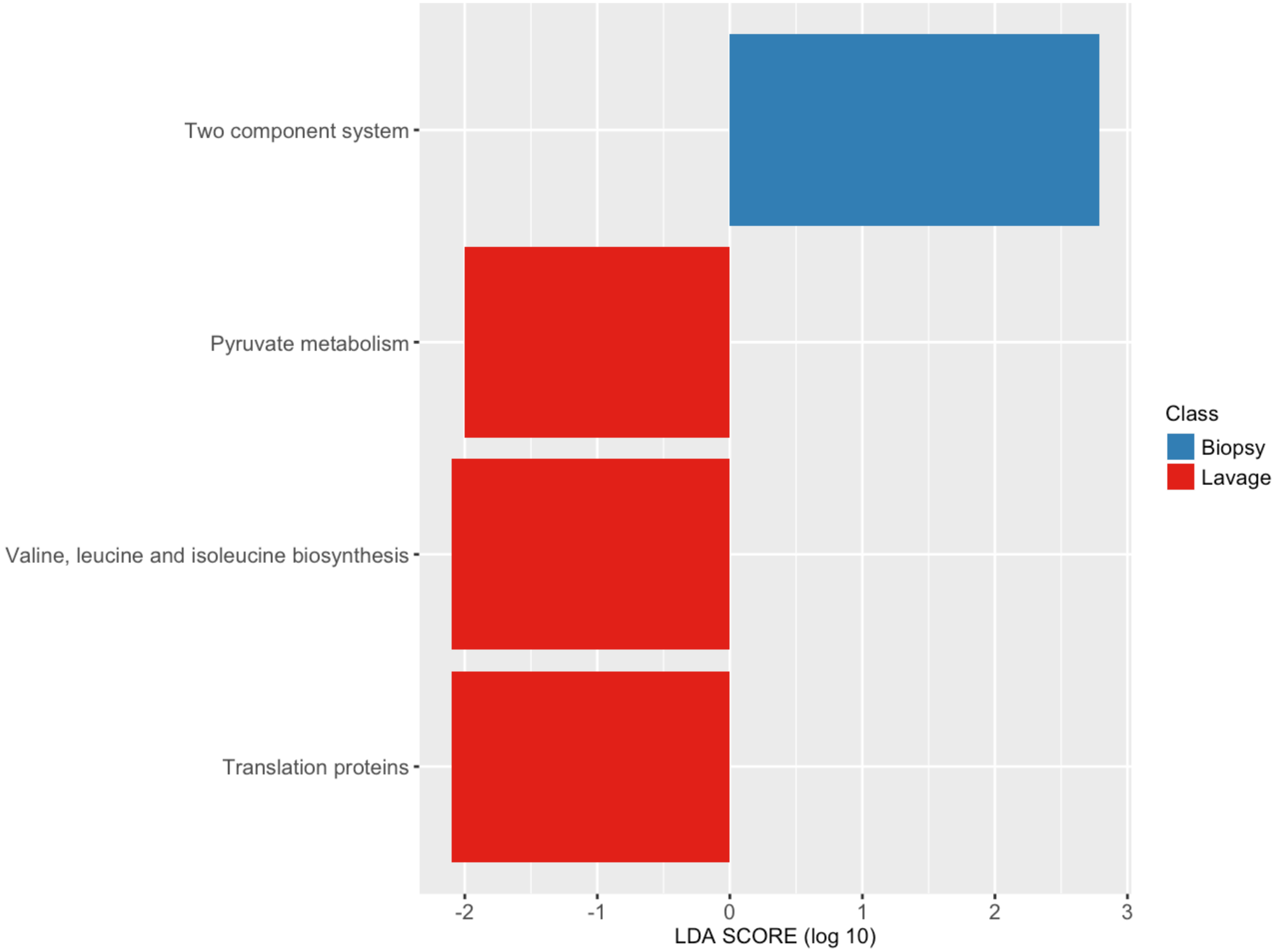

Supplementary figure 6: Average relative abundance of genera within biopsy and lavage samples matching contaminant genera from

Salter et al 2014

```
### Load packages or install if not present
if (!require("RColorBrewer")) {install.packages("RColorBrewer")
  library(RColorBrewer)}
if (!require("ggplot2")) {install.packages("ggplot2")
  library(ggplot2)}
if (!require("tidyr")) {install.packages("tidyr")
  library(tidyr)}
#Input file names
taxa_table_file <- "16sBxCL_contamination_genera_bar_chart.txt"
#Set name of PDF output file
PDF_file <- "SuppFig6.pdf"
#Read in files
taxa_data <- read.csv(taxa_table_file, sep = "\t")
```

```
## Warning in read.table(file = file, header = header, sep = sep,
## quote = quote, : incomplete final line found by readTableHeader on
## '16sBxCL_contamination_genera_bar_chart.txt'
```

```
#Get ID info
ID.info <- taxa_data[3,]
#Only relabund info
relabund.info <- taxa_data[1:2,]
#Change data to long list format
relabund.info_long <- gather(relabund.info, Genera, relabund, (2:(ncol(relabund.in
fo))))
```

```
## Warning: attributes are not identical across measure variables; they will
## be dropped
```

```
#Change . in genera names to space
relabund.info_long$Genera <- gsub('([[:punct:]])|\\s+', ' ', relabund.info_long$Ge
nera)
#Select colour palette for plot
colset <- c(brewer.pal(8, "Set1"), brewer.pal(8, "Set2"), brewer.pal(12, "Set3"),
brewer.pal(9, "Pastel1"))
#Plot the data
g_bar <- ggplot(relabund.info_long, aes(x=Genera, y=(as.numeric(relabund)), fill=S
ample.type)) + geom_bar(stat = 'identity', position = 'dodge', width=0.95) + scale
_fill_manual(values = c(colset[2], colset[1])) + theme_set(theme_gray(base_size =
8)) + theme(axis.text.x = element_text(angle = 90, hjust = 1, size=8)) + xlab("Gen
era") + ylab("Average Relative abundance") + scale_y_continuous(expand = c(0,0.000
1)) + labs(fill='') + coord_flip()
ggsave(PDF_file, g_bar, units="mm", height=140, width=170, dpi=300)
```

```
## Warning in read.table(file = file, header = header, sep = sep,
## quote = quote, : incomplete final line found by readTableHeader on
## '16sBxCL_contamination_genera_bar_chart.txt'
```

```
## Warning: attributes are not identical across measure variables; they will
## be dropped
```

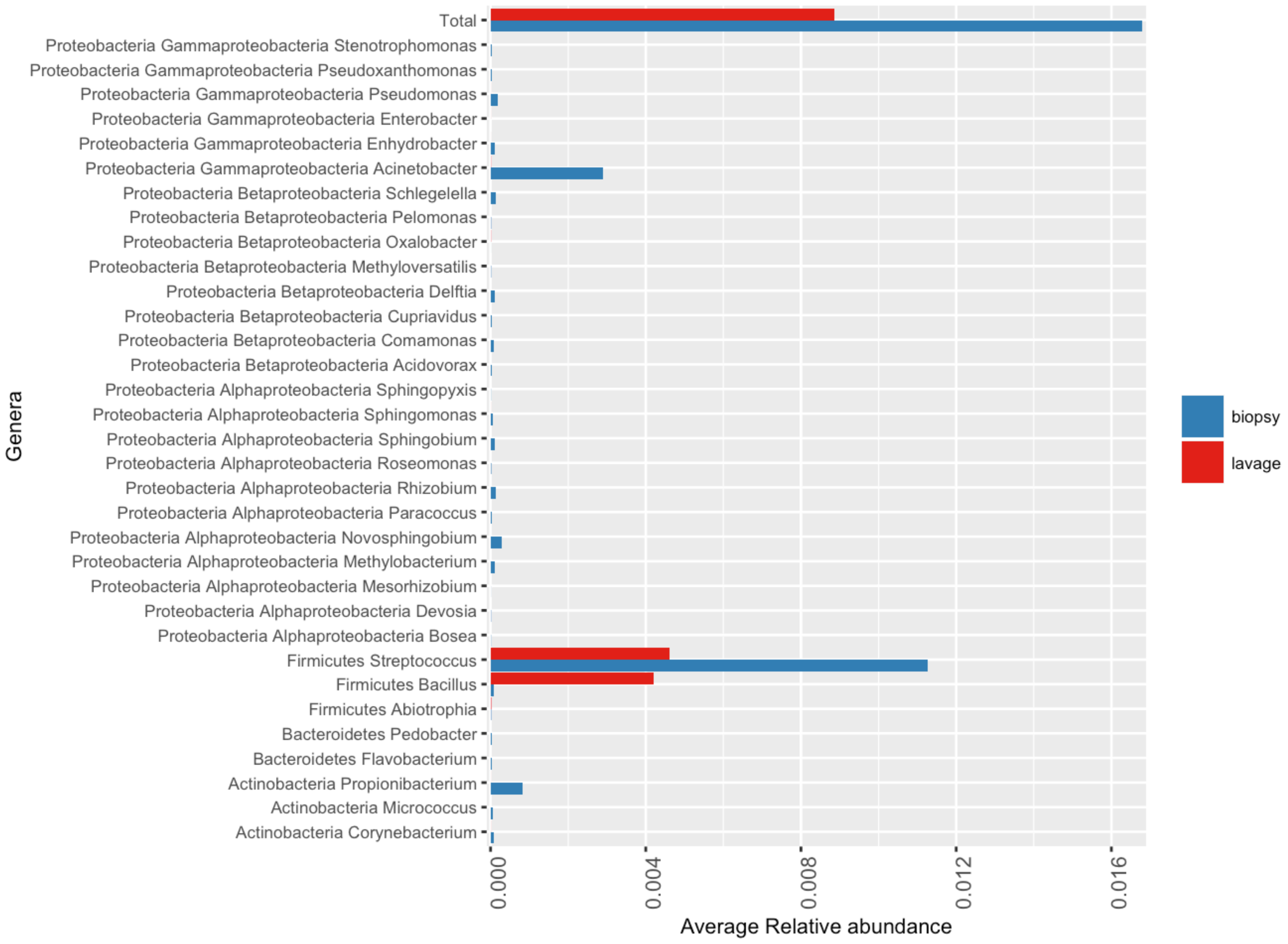

Supplement: Additional file 12: — Full account of figure production by R. (PDF 3.32 mb) [file 40168_2016_207_MOESM12_ESM.pdf]
