# Supplementary material for: Extending colonic mucosal microbiome analysis—assessment of colonic lavage as a proxy for endoscopic colonic biopsies
Source: Microbiome. 2016 Nov 25;4:61. doi: 10.1186/s40168-016-0207-9 (PMC5123352; doi:10.1186/s40168-016-0207-9)
Supplement: Additional file 6: Figure S4. — Assessment of the uncertainty in the hierarchical cluster analysis when clustering. (A) Log2 count of sequences within families. (B) Differentially abundant OTUs between biopsy and lavage samples by LefSe and (C) the top 50 OTUs found to not be differentially abundant between biopsy and lavage samples by LEfSe. The red values are the approximately unbiased (AU) p values, the green values are the bootstrap possibility (BP) values and the grey values are the node numbers. Clusters encased by red squares indicate clusters with AU p values > 95. One thousand bootstraps were utilised. (PDF 80 kb) [file 40168_2016_207_MOESM6_ESM.pdf]

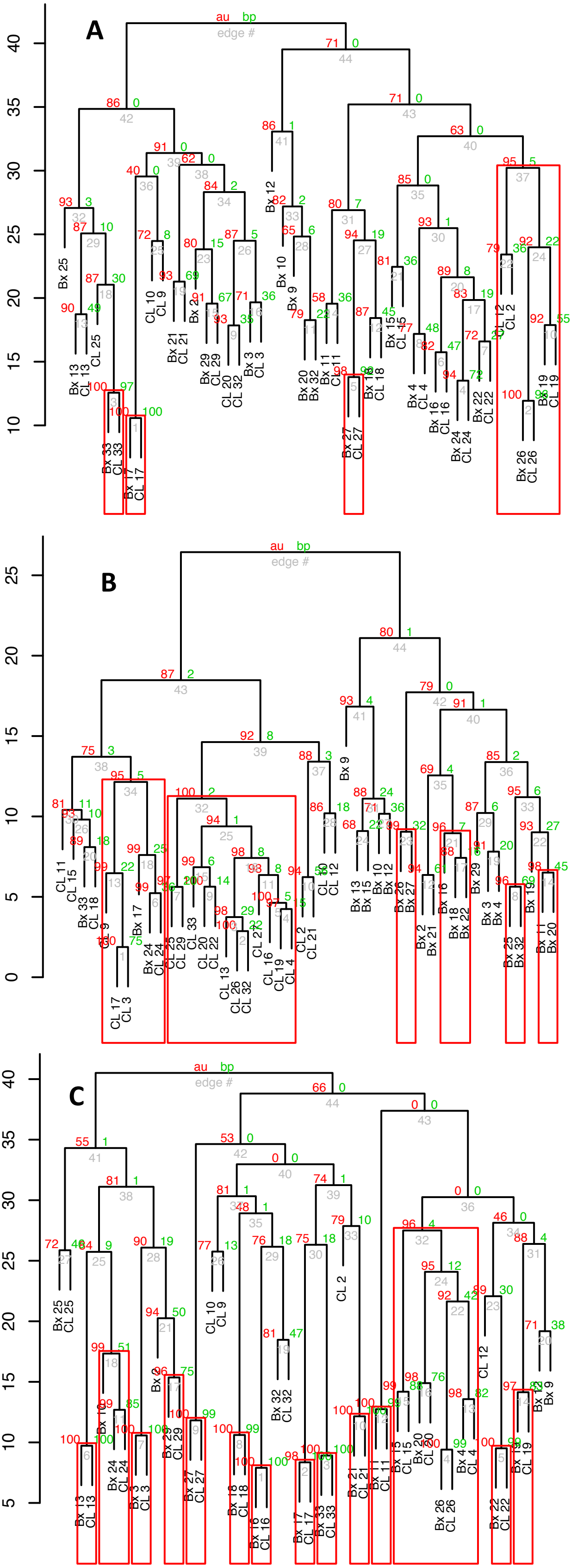
A
au
bp
edge #
B
C
