# Supplementary material for: Extending colonic mucosal microbiome analysis—assessment of colonic lavage as a proxy for endoscopic colonic biopsies
Source: Microbiome. 2016 Nov 25;4:61. doi: 10.1186/s40168-016-0207-9 (PMC5123352; doi:10.1186/s40168-016-0207-9)
Supplement: Additional file 10: Figure S6. — Predicted KEGG pathway comparison between colonic biopsy and lavage sample. The extent of microbiota predicted functional differences between sample types were measured using (A) KEGG pathway abundance and (B) Alpha diversity scores. Alpha diversity scores calculated by subsampling samples to 500000 KEGG orthologs with 1000 iterations. Each point represents the diversity score for a patient sample. Error bars represent SEM. Between-group variations were measured using Mann-Whitney U test. P values of Mann-Whitney U test for each alpha diversity measure are below the relevant facet, (C) LEfSe LDA scores. (PDF 288 kb) [file 40168_2016_207_MOESM10_ESM.pdf]

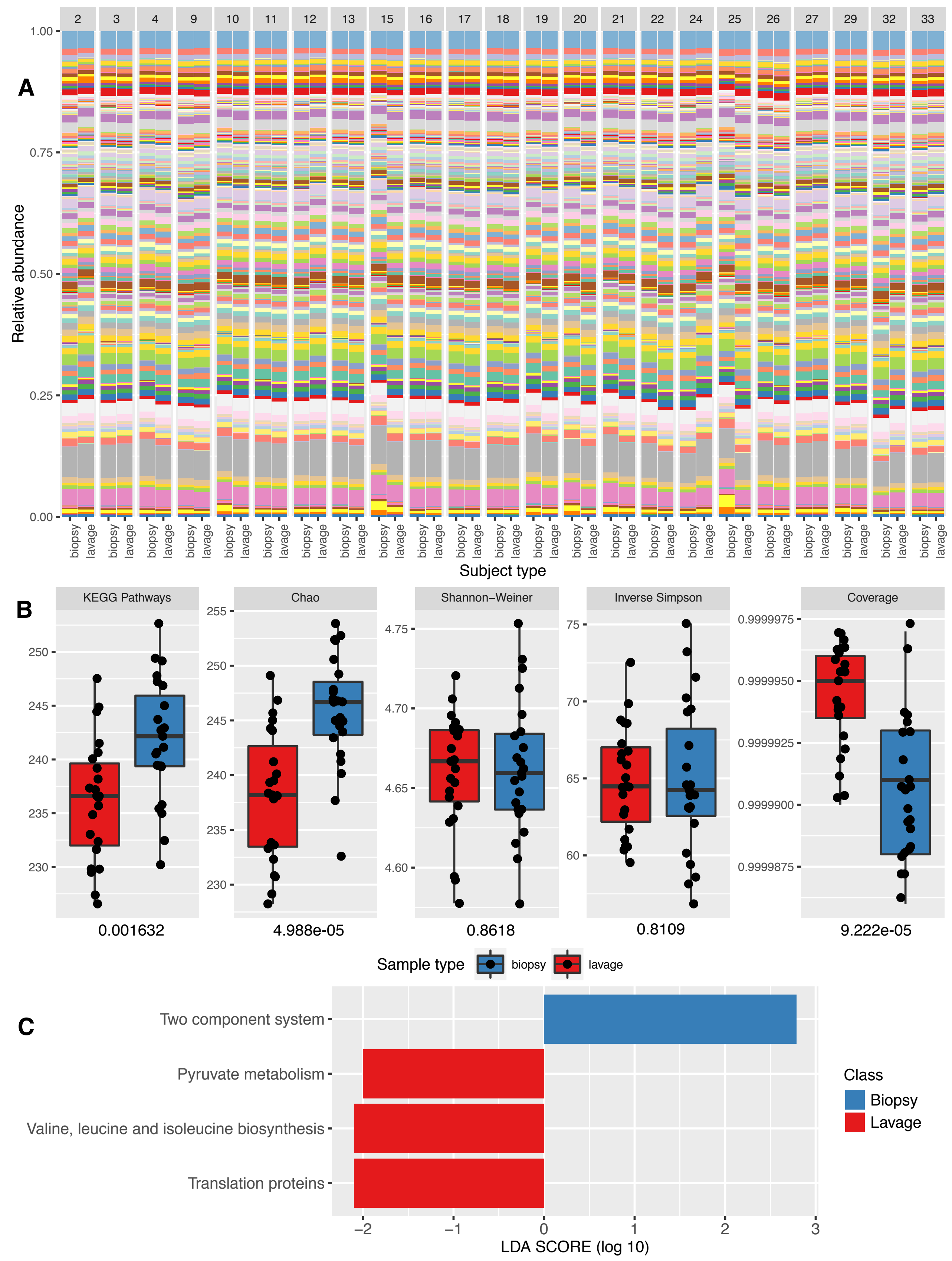
A
B
C
Relative abundance
Subject type
2 3 4 9 10 11 12 13 15 16 17 18 19 20 21 22 24 25 26 27 29 32 33
biopsy
lavage
KEGG Pathways
Chao
Shannon–Weiner
Inverse Simpson
Coverage
0.001632
4.988e-05
0.8618
0.8109
9.222e-05
Sample type
biopsy
lavage
Two component system
Pyruvate metabolism
Valine, leucine and isoleucine biosynthesis
Translation proteins
Class
Biopsy
Lavage
LDA SCORE (log 10)
